# The Effectiveness of Parent-Targeted Digital Health Interventions on Breastfeeding Practices: Systematic Review and Meta-Analysis of Randomized Controlled Trials

**DOI:** 10.2196/89214

**Published:** 2026-07-02

**Authors:** Jacklyn Jackson, Sienna Kavalec, Alison L Brown, Tessa Delaney, Nayerra Hudson, Anna Rayward, Ben Singh, Lisa Sahlin Torp, Rebecca Liackman, Kayla Pennicott, Kristen Saunders, Sonya Stanley, Luke Wolfenden, Melanie Kingsland, Rachel Sutherland

**Affiliations:** 1 School of Medicine and Public Health, University of Newcastle Australia, University Drive, Newcastle, New South Wales, 2308, Australia, 61 2 4921 6664; 2 Hunter New England Local Health District, Newcastle, New South Wales, Australia; 3 Hunter Medical Research Institute, Newcastle, New South Wales, Australia; 4 National Centre of Implementation Science, Newcastle, New South Wales, Australia; 5 NSW Ministry of Health, Sydney, NSW, Australia; 6 Alliance for Research in Exercise Nutrition and Activity, University of South Australia, Adelaide, South Australia, Australia; 7 Department of Public Health and Social Sciences, Uppsala University, Uppsala, Uppland, Sweden

**Keywords:** breastfeeding, digital health, mHealth, mobile health, eHealth, systematic review, meta-analysis, self-efficacy

## Abstract

**Background:**

Global breastfeeding rates remain below recommended levels. Parent-targeted digital health interventions (DHIs), including mobile health (mHealth) and eHealth strategies, offer a scalable way to support breastfeeding, but their effectiveness remains uncertain.

**Objective:**

The aim of this study is to explore the effectiveness of parent-targeted DHIs for improving breastfeeding outcomes.

**Methods:**

In total, 7 databases were searched on December 9, 2025, for randomized controlled trials (RCTs) involving parents of children younger than 5 years of age. Eligible interventions aimed to promote breastfeeding and were primarily delivered via digital mediums. Primary outcomes of interest included exclusive breastfeeding (EBF), any breastfeeding, and breastfeeding duration. Secondary outcomes included breastfeeding self-efficacy, cost-effectiveness, and adverse events. Random effects meta-analyses were conducted in accordance with Cochrane methods, and results were reported following PRISMA (Preferred Reporting Items for Systematic Reviews and Meta-Analyses) guidelines. Risk of bias was assessed using the Cochrane Risk of Bias 2 tool, and certainty of evidence was graded using the Grading of Recommendations Assessment, Development, and Evaluation approach.

**Results:**

In total, 46 (39 RCTs and 7 cluster RCTs) studies, including 33,785 participants from 18 diverse countries, were included. A total of 25 of the interventions focused on mHealth strategies, 8 were delivered via computer-based eHealth, 4 by telehealth, and 9 were delivered by eHealth or mHealth combined with telehealth. Risk of bias was indicated with “some concerns” or “high risk” for 41 (89%) studies. Pooled results indicated that DHIs can significantly improve the odds of EBF (odds ratio 2.58, 95% CI 1.91-3.50; *I*^2^=83%; 39 trials, 11,601 participants); however, considerable heterogeneity was present, and certainty of evidence was very low. Pooled results indicated with moderate certainty that DHIs may improve breastfeeding duration (standardized mean difference 0.48, 95% CI 0.29-0.67; *I*^2^=0%; 7 trials, 716 participants). Results suggest that DHIs have no effect on the odds of any breastfeeding (odds ratio 1.09, 95% CI 0.90-1.31; *I*^2^=19%; 21 trials, 8991 participants), but the certainty of evidence is very low. Cost or cost-effectiveness and adverse events were scarcely reported.

**Conclusions:**

This review provides a comprehensive synthesis of global evidence exploring the impacts of parent-targeted DHIs on breastfeeding outcomes, spanning diverse cultural and health system contexts. Our results suggest that parent-targeted DHIs represent a promising strategy for improving key breastfeeding indicators, such as EBF and breastfeeding duration with very low to moderate certainty, as current evidence is limited by variable risk of bias, potential publication bias, and substantial heterogeneity. DHIs could have a complementary role as part of the health care and support provided to parents during the first 2000 days. Future trials should seek to minimize possible biases as well as capture key scale-up outcomes to justify embedding such innovations within existing health systems and structures.

## Introduction

Breastfeeding represents an equitable and environmentally sustainable practice that supports the short-term and long-term health of children and parents [1]. For example, longer breastfeeding duration has been associated with lower risk of type 2 diabetes, reduced risk of childhood and adulthood obesity [2], reduced risk of infections [3,4], increased fine and gross motor development, enhanced cognitive development, and higher IQ scores in children [5,6]. Further, breastfeeding parents experience a 26% lower risk of breast cancer, a 37% lower risk of ovarian cancer, and a 32% lower risk of developing type 2 diabetes [7], with evidence also indicating a protective association between breastfeeding and maternal mental well-being, including lower rates of self-reported depression and anxiety [8]. Breastfeeding can support key educational and societal goals and is associated with better learning skills and educational outcomes in school-aged children [9,10]. Due to reduced risk of infant or childhood illnesses and hospitalization, breastfeeding also contributes to significant cost savings in health care [11]. Hence, there is substantial public health and societal value in supporting caregivers and children to initiate and sustain breastfeeding well beyond the first few months of life.

Due to the many benefits offered by breastfeeding, international health organizations including the World Health Organization recommend exclusive breastfeeding (EBF) for the first 6 months of life, with the continuation of breastfeeding to 2 years and beyond (as mutually desired by both the breastfeeding parent and child) [12-14]. However, despite these recommendations, global breastfeeding rates are suboptimal [15,16]. A 2021 systematic review by Vaz et al [17] found that current breastfeeding practices in most high-income countries fall short of recommendations, with a median of only 18% of infants sustaining EBF to 6 months and only 29% sustaining any breastfeeding to 12 months. While breastfeeding rates to 12 months in middle- and low-income countries are much higher (80%‐90%), EBF rates to 6 months also remain low (40%) [18]. Additionally, despite international data suggesting that breastfeeding initiation rates are relatively high (approximately 91%), breastfeeding rates markedly decline during the early postnatal period (ie, the first 6‐8 weeks after childbirth) [18,19].

A narrative review by Jackson et al [20] highlights that a parent’s ability to continue breastfeeding is shaped by a complex range of individual, interpersonal, organizational, community, and policy-based determinants. For example, a major determinant of breastfeeding establishment and sustainment includes an individual’s ability to overcome common breastfeeding challenges, such as difficulties with attachment, engorged breasts, sore nipples, milk stasis, and mastitis. A key determinant of breastfeeding duration also includes parental breastfeeding intentions, experiences, attitudes, beliefs, and self-efficacy. While health system-level support is vital for driving improved breastfeeding initiation rates [21], ongoing lactation support beyond initial hospital care is often fragmented and limited due to inadequate staff availability and training [22,23]. Despite the complexity of breastfeeding determinants, a Cochrane systematic review including 116 trials concluded that “breastfeeding only” support can improve the duration of EBF and reduce the number of women ceasing breastfeeding at 3‐4 months [24]. While the review found that effective breastfeeding support could be offered by a range of modalities (eg, face-to-face, telephone or digital technologies, or in combination), it was evident that further work to identify the components of effective and scalable interventions is needed to maximize the impact of benefits at the population level [24].

Digital health interventions (DHIs) including eHealth and mobile health (mHealth) interventions that are delivered via digital means including computers, websites, smartphone apps, text messages, and social media platforms represent a promising method for providing evidence-based health care at scale [25]. Proliferation of digital health care solutions is expected to help ease the pressure placed on traditional face-to-face health care services while reducing rising health care costs and health inequalities [26]. As such, the integration of innovative and evidence-based digital health solutions is of increasing interest to government or health agencies internationally, with the expectation that digital health solutions will become a foundational element of all modern health service delivery models [26].

Previous published reviews have explored the impacts of parent-targeted DHIs within relatively narrow criteria. For example, a systematic review by Corkery-Hayward and Talaei [27] focused on interventions targeting low-income women within high-income countries. A 2016 systematic review and meta-analysis by Lee et al [28] included studies only conducted within lower-middle income countries, and a meta-analysis by Sun et al [29] focused only on internet-based interventions, suggesting that an updated review and meta-analysis including studies conducted in high-, middle-, and low-income countries and examining a comprehensive range of DHIs would be of interest. Therefore, the aim of this systematic review and meta-analysis was to evaluate the effectiveness of parent-targeted DHIs (conducted in high-, middle-, and low-income countries) for improving breastfeeding outcomes. A secondary aim was to evaluate the impact of parent-targeted DHIs on breastfeeding self-efficacy, as well as explore evidence around their cost or cost-effectiveness and adverse effects.

## Methods

### Overview

A protocol for this systematic review was prospectively published in PROSPERO (CRD42023492644) [30] and included a variety of outcomes related to physical activity, diet, and sedentary behavior; however, this paper reports on the breastfeeding outcomes only. This systematic review and meta-analyses were conducted as per methods outlined in the Cochrane Handbook [31] and reported as per the PRISMA (Preferred Reporting Items for Systematic Reviews and Meta-Analyses) guidelines [32] (Checklist 1) as well as the PRISMA-S (Preferred Reporting Items for Systematic Reviews and Meta-Analyses-Searches) checklist [33] (Checklist 2), and PRISMA checklist for Abstracts (Checklist 3).

### Eligibility Criteria

#### Types of Studies

Parallel group randomized controlled trials (RCTs), cluster RCTs, and factorial RCTs were eligible for inclusion. Full-text papers published in the English language within peer-reviewed journals or within dissertations were eligible for inclusion. Conference abstracts without associated full-text papers were excluded.

#### Types of Participants

Eligible participants were generally healthy parents or caregivers (hereafter referred to as parents) of generally healthy children younger than 5 years of age. Parents could be recruited from any setting (eg, community, health care, or education settings), but the DHI needed to target breastfeeding behaviors outside the clinical setting.

Given the importance of generalizing the review findings, studies were excluded if they sampled participants (parents or infants) exclusively from populations with specific or preexisting medical conditions including (but not limited to) malnutrition, cystic fibrosis, cerebral palsy, preterm infants (ie, born before 36-week gestation), or disease diagnoses (ie, gestational diabetes or HIV).

#### Types of Interventions

This review included DHIs delivered to parents from conception to anytime postnatally, provided the intervention was intended to improve breastfeeding behaviors beyond the immediate birth of the child.

DHIs have been defined as “health interventions delivered through digital tools or communication technologies which collect, store, share and analyse health information for purposes of improving patient health and health care delivery” [34]. As such, DHI can be delivered through a broad range of digital tools including but not limited to wearable devices, mobile apps, texting through smartphones, websites, and telehealth platforms [35]. While a lack of consensus on DHI terminology remains, a review by Mangion and Piller [36] suggest that digital health relates to “an umbrella term used for all digital technologies to improve health and wellbeing”; eHealth relates to “DHIs delivered through the use of information and communication technologies”; computer-based eHealth includes “the use of desktop or web applications to deliver health services”; mHealth includes the “use of mobile technology, delivered via mobile devices or wearables, to deliver health services”; uHealth relates to “Health monitoring that takes place anytime, anywhere, typically through the integration of mobile and wearable technologies”; and telehealth involves “remote delivery of health services using telecommunications technologies, which may operate through mHealth or computer-based systems.”

This review included DHIs whereby the intervention content was primarily predetermined or prescribed (with content tailoring limited to a set of predefined pathways) to ensure that DHIs included were likely scalable at a population level. Therefore, interventions that were exclusively clinician-provided and included personalized one-on-one consultations via digital means (ie, videoconferencing or calls in place of face-to-face) were excluded from the review. Interventions that included digital and nondigital components were excluded from the review if the intervention clearly had a greater focus on the nondigital components.

#### Control

Eligible trials compared a parent-targeted DHI to one of the following types of control groups: (1) nonintervention control, (2) waitlist control, (3) nondigital usual care control, or (4) an attention control that did not seek to influence breastfeeding.

#### Types of Outcomes

Included studies reported on at least one of the following primary outcomes:

- Breastfeeding status: That is, the number or proportion of babies or infants “exclusively breastfeeding” or receiving “any breastmilk” at a specific time point (outside of the hospital after birth). Whereby EBF relates to infants (generally 6 months or less) who receive only breastmilk and no other liquids or solids (not even water). While “any” breastfeeding can relate to an infant or child who is receiving breastmilk with the presence of any other fluids or semisolid or solid food [37].
- Breastfeeding duration: That is, the mean duration of “exclusive” or “any” breastfeeding (reported in days, weeks, or months).

Where available in the main trial report, secondary outcomes of interest included:

- Parental or maternal breastfeeding self-efficacy, which is a measure of confidence in the ability to successfully breastfeed. Breastfeeding self-efficacy has been identified as a key modifiable factor for enabling EBF and breastfeeding duration [38].
- Intervention cost or cost-effectiveness: For example, absolute or crude intervention costs, implementation costs, and cost-effective estimates or ratios.
- Unintended adverse events as identified and reported within the published papers. If a study reported “no known adverse events,” this outcome was also extracted.

### Information Sources

#### Electronic Searches

The following electronic databases were originally searched on April 15, 2024, with an updated search conducted on December 9, 2025: CENTRAL in the Cochrane Library (up to November 2025), CINAHL Complete (EBSCO; 1981 to December 2025), Education Research Complete (EBSCO; up to December 2025), Embase (OVID; up to December 2025), MEDLINE (OVID; 1946 to December 12, 2025), PsycINFO (OVID; 1806 to December 2025), and Scopus (before 1960 to December 2025).w

No time restrictions were placed on the original search. Databases were searched separately, and as per protocol, study registry searches were not undertaken.

#### Unpublished or Gray Literature Searches

The first 100 search results from Google and Google Scholar were screened for relevant unpublished or gray literature using the following terms: “digital,” or “mHealth” or “app,” and “breastfeeding.”

#### Searching Other Sources

Two members of the research team (SK and JJ) conducted hand searches of the following: reference lists of included studies and reference lists of relevant systematic reviews identified from the electronic search.

Authors of relevant protocol papers identified during the electronic search were also contacted.

### Search Strategy

Search terms for “Digital Health Interventions” were adapted from previous systematic reviews to suit our research question [39-41] and reviewed by an experienced research librarian.

The search was based on broad domains using MeSH related to “Breastfeeding” combined with “Parents/carers” or “Infant/Newborn” combined with “Digital Health Intervention.” Cochrane recommended filters were applied to help identify RCTs, and limits were applied to the database searches (where possible) to identify human studies [42]. The search terms for each electronic database are listed in Multimedia Appendix 1.

### Selection Process

Following deduplication of identified titles and abstracts using the “find duplicate” function in EndNote (Clarivate; matching references for author, year, title, and reference type), additional duplicates were identified and removed within Covidence. Further, non-RCTs were removed prior to title and abstract screening using Covidence artificial intelligence filters [43].

Following deduplication of references, pairs of review authors (JJ, AR, ALB, RL, KP, KS, SS, and TD) independently screened titles and abstracts using Covidence software. Where discrepancies arose or where there was insufficient detail to determine eligibility based on the title or abstract, studies were advanced to full-text review.

Pairs of review authors (JJ, ALB, BS, RL, and NH) independently assessed full-text papers for eligibility using Covidence. Where discrepancies between reviewers for study inclusion could not be clearly reconciled by the lead reviewer (JJ), a field expert (RS) was consulted as a third reviewer to determine final study inclusion.

### Data Collection Process

Study characteristics were extracted by 1 author (SK) and verified by the lead reviewer (JJ), using an adapted and piloted version of the Cochrane Public Health data extraction template. Extracted data included author, location, design, population, and intervention description (as per the TIDieR [Template for Intervention Description and Replication] checklist [44]) and comparator components, duration, setting, sample size, participants’ age, outcomes, and results.

Primary outcome data were extracted independently by the lead reviewer (JJ) and checked by a second reviewer (SK). Any discrepancies were resolved by consensus.

### Synthesis Methods

#### Intervention Components

Intervention components were coded to the socioecological model (SEM) for breastfeeding [45]. As per the SEM, interventions could target individual factors, interpersonal factors, community factors, or policy. Based on intervention descriptions, the included interventions were classified into DHI type according to the Mangion and Piller [36] definitions. In circumstances where classifications of interventions as eHealth or mHealth were not clear based on intervention descriptions, our classifications were guided by the language used to refer to the intervention within the published paper (ie, text message or smartphone intervention was classified as mHealth) and/or intervention inclusion criteria (ie, if participants needed a smartphone to be considered for inclusion, the intervention was classified as mHealth).

#### Dichotomous Data

For EBF rates and rates of any breastfeeding, data were pooled in a random-effects meta-analysis using RevMan (Cochrane’s Review Manager). Odds ratios (ORs) were calculated using the generic inverse variance method, and corresponding 95% CIs were calculated by the Hartung-Sidik-Jonkman method [46]. Where breastfeeding rates for a single study had been reported at multiple time points, data corresponding with the longest follow-up period reported were included.

#### Continuous Data

Where available, data related to breastfeeding duration and breastfeeding self-efficacy were combined in a random-effects meta-analysis to calculate the standardized mean difference (SMD) using the generic inverse variance method in RevMan. Corresponding 95% CIs were calculated using the Hartung-Knapp-Sidik-Jonkman method [46].

#### Cluster RCTs

When clustering had not been adjusted for in the analysis reported within the study, the effective sample size for cluster RCT data was calculated using methods outlined in the Cochrane Handbook [31].

#### Trials With Multiple Groups

Aligned with Cochrane methods, if studies had 1 control group and 2 different relevant intervention groups, the control group number of events and participants were split in half to avoid “double counting” control participants for dichotomous outcomes [31]. For continuous outcomes, the intervention arms of interest were combined in RevMan to create a single pair-wise comparison, as recommended in the Cochrane Handbook [31].

#### Sensitivity Analysis

Sensitivity analyses based on the study risk of bias were performed by removing the high risk of bias studies from the analyses.

#### Subgroup Analyses and Investigation of Heterogeneity

Heterogeneity was considered substantial if the *I*^2^ was greater than 30% and either τ^2^ was greater than 0 or there was a low *P* value (<.10) in the chi-square test for heterogeneity. Based on this, the subgroup analyses were conducted for EBF and “any” breastfeeding, as they also contained an adequate number of studies to explore subgroup effects. Subgroups included:

- The time to outcome measurement: International breastfeeding guidelines including the World Health Organization [37] recommend EBF up to 6 months, with continued breastfeeding alongside the introduction of solid foods up until 2 years and beyond. Based on this, we used cutoff points of 6 months versus <6 months for the EBF meta-analysis. For “any” breastfeeding, we used cutoff points of ≥12 months versus 6 months to <12 months versus <6 months.
- The DHI modality according to definitions for Mangion and Piller [36] (ie, computer-based eHealth vs mHealth vs telehealth vs mixed DHI modalities).
- The timing of support (prenatal support vs only postnatal support).
- The type of participants engaged (mothers only vs parents or caregivers).
- The study country income level based on the World Bank classifications [47] (high-income vs upper or middle income vs low or lower middle income).

To explore how the effect sizes varied across studies included in meta-analyses, 95% prediction intervals were calculated for each meta-analysis, using methods outlined in the Cochrane Handbook [48]. This method is recommended for exploring heterogeneity when there are a reasonable number of studies included in the meta-analysis (ie, 5 or more), and there is no clear funnel plot asymmetry. Prediction intervals can indicate highly probable values for the true treatment effects in future studies [49].

#### Synthesis Without Meta-Analysis

Where only 1 trial reported on a particular outcome, or where meta-analysis was not possible, relevant findings of each trial were individually summarized.

### Study Risk of Bias Assessment

Pairs of unblinded review authors (JJ, SK, AR, RL, and LST) independently assessed the risk of bias of included studies using the Cochrane Risk of Bias 2 tool for randomized trials [50]. Additionally, as per the Cochrane Handbook, the risk of bias assessment focused on the main outcome of the review (breastfeeding status) and assumed an intention-to-treat effect (ie, based on assignment to the intervention) [51].

All recommended domains were assessed including (1) randomization process, (2) deviations from intended interventions, (3) missing outcome data, (4) measurement of the outcome, and (5) selection of the reported result. If the study was a cluster RCT, additional relevant criteria were assessed relating to the timing of identification or recruitment of participants. Overall study risk of bias was based on the algorithm recommended in the Cochrane Handbook [51]. For example, studies were considered high risk of bias overall if one or more risk of bias domains were assessed as high risk.

### Certainty of Evidence

The certainty of evidence related to our primary outcomes was evaluated using the Grading of Recommendations Assessment, Development, and Evaluation (GRADE) approach to categorize the certainty of evidence as “high,” “moderate,” “low,” or “very low.” Two authors (JJ and SK) used the GRADEpro GDT software (Evidence Prime Inc) to conduct separate summaries of findings and assessments of the level of evidence [52]. As part of the GRADE assessment, evidence was evaluated based on risk of bias, inconsistency, indirectness, imprecision, and other biases including publication bias and small study effects (by examining funnel plot asymmetry) [53]. Any discrepancies between author assessments were resolved by consensus.

## Results

### Results of the Search

**Figure 1.**
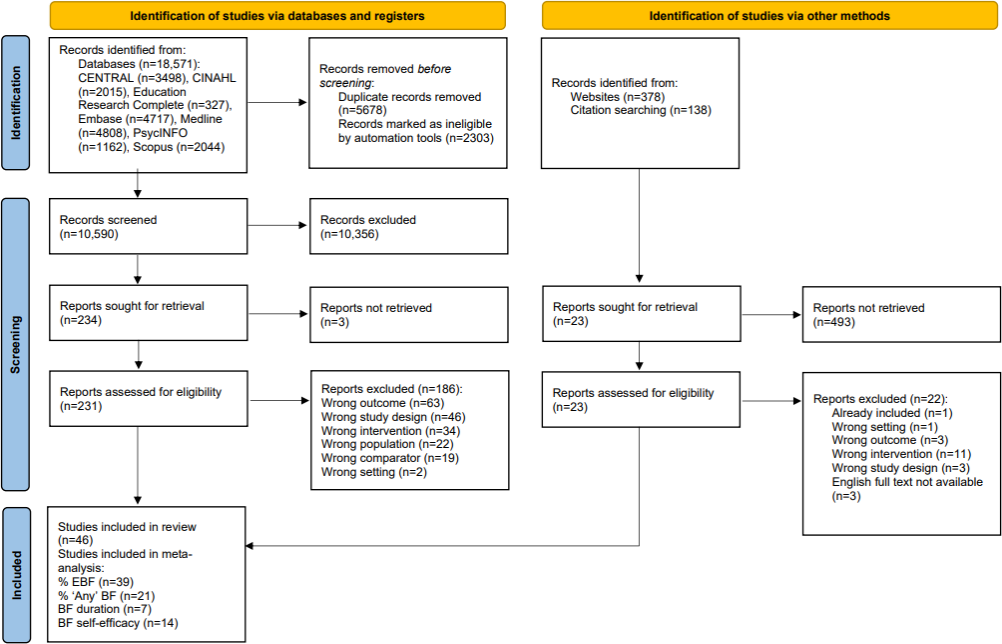
PRISMA flow diagram of study selection process. BF: breastfeeding; EBF: exclusive breastfeeding; PRISMA: Preferred Reporting Items for Systematic Reviews and Meta-Analyses.

The search identified a total of 11,106 records that underwent title and abstract screening (see Figure 1 for PRISMA flow diagram). Of these, 254 full-text papers were reviewed for eligibility, and a total of 208 records were excluded at full-text screening (primarily due to studies not reporting on the primary outcome: n=66, wrong study design: n=49, or wrong intervention: n=45). A total of 46 studies (all published in peer-reviewed journals) met the review eligibility criteria and were included within the review.

### Included Studies

Within the 46 included studies, there were 96 trial arms and 33,785 participants. Key details of the studies including study design, country, details of the DHI, intervention and comparator participants, outcomes, time points of measurement, and method of synthesis are included in Table 1.

**Table 1.** Overview of synthesis and included studies: including study design, population, digital health intervention (DHI) modality, comparator type, review synthesis method, and outcome assessment time points.

Author (year), study design, country	DHI mode and control type	Population and sample size	Outcome domains with available data	Method of synthesis in review	Time point of measurement
Abbass-Dick et al (2020) [54], RCT^a^, Canada	Computer-based eHealth Nondigital usual care control	Expectant mothers (>25-week gestation) and their coparents Intervention: n=56 mothers and n=50 coparents Control: n=57 mothers and n=54 coparents	BF^b^ BF self-efficacy	EBF^c^ (MA)^d^ BF self-efficacy scale—short form (MA)	4, 12, and 26 weeks after birth Baseline, 2, and 4 weeks after birth
Acar and Şahin (2024) [55], RCT, Turkey	mHealth^e^+uHealth Nondigital usual care control	Mothers of newborns Intervention: n=40 Control: n=40	BF	EBF (MA)	4 and 8 weeks after birth
Ahmed et al (2016) [56], RCT, United States	Computer-based eHealth+uHealth Nondigital usual care control	Mothers of healthy infants and intending to continue BF Intervention: n=49 Control: n=57	BF	EBF (MA)	1, 2, and 3 months after hospital discharge
Araban et al (2018) [57], RCT, Iran	mHealth Nondigital usual care control	Expectant mothers (35‐ to 37-week gestation) Intervention: n=60 Control: n=60	BF BF self-efficacy	EBF (MA) “Any” BF (MA) BF self-efficacy scale—short form (MA)	8 weeks after birth
Baransel and Çalışkan (2024) [58], RCT, Turkey	mHealth Nondigital usual care control	Mothers of newborns Intervention: n=70 Control: n=72	BF BF self-efficacy	EBF (MA) BF self-efficacy scale—short form (MA)	Baseline and 6 weeks after birth Baseline and 6 weeks after birth
Bender et al (2022) [59], RCT, United States	mHealth Attention control	Expectant mothers (34‐ to 38-week gestation) Intervention: n=106 Control: n=110	BF	EBF (MA) “Any” BF (MA)	6 weeks after hospital discharge
Bilgiç and Bozkurt (2024) [60], RCT, Turkey	Computer-based eHealth+telehealth Nondigital usual care control	Expectant mothers (32‐ to 38-week gestation) Intervention: n=55 Control: n=55	BF	EBF (MA)	Postbirth, 1, 2, and 3 months after birth
Bogaert et al (2024) [61], RCT, United States	mHealth Nondigital usual care control	Mothers discharged from hospital with their newborn Intervention: n=97 Control: n=94	BF	“Any” BF (MA)	Reported at 6-week postpartum visit
Bunik et al (2022) [62], RCT, United States	mHealth+uHealth Attention control	Expectant mothers (>36-week gestation) Intervention: n=310 Control: n=157	BF BF self-efficacy Adverse events	EBF (MA) “Any” BF (MA) BF self-efficacy scale—long form (MA) Adverse events (summary)	3 and 6 months after birth 3 and 6 months after birth 3 and 6 months after birth Study duration
Can and Bulduk (2025) [63], RCT, Turkey	Computer-based eHealth Nonintervention control	Adolescent (15‐19 years) expectant mothers (28‐ to 37-week gestation) Intervention: n=50 Control: n=50	BF BF self-efficacy	EBF (MA) BF self-efficacy scale—short form (MA)	1 and 8 weeks post partum
Cavalcanti et al (2018) [64], RCT, Brazil	Computer-based eHealth Attention control	Mothers discharged from hospital with their newborn Intervention: n=123 Control: n=128	BF	EBF (MA) EBF duration (MA)	1, 2, 3, 4, 5, and 6 months after birth
Çelik and Toruner (2024) [65], RCT, Turkey	mHealth+telehealth Nondigital usual care control	First-time adolescent (15‐19 years) mothers of full-term infants Intervention: n=17 Control: n=17	EBF BF self-efficacy	EBF (MA) BF self-efficacy scale—short form (MA)	1, 4, and 8 weeks after birth 1 and 8 weeks after birth
Chegeni et al (2022) [66], 3-arm RCT, Iran	Telehealth+ mHealth Nondigital usual care control	First-time mothers Intervention: n=87 phone and n=87 online Control: n=87	BF BF self-efficacy	EBF duration (MA) BF self-efficacy scale—long form (MA)	10 days, 1 and 4 months after birth Baseline, 10 days, and 1-month posthospital discharge
Davis et al (2023) [67], RCT, United States	mHealth Attention control	Parents of newborns Intervention: n=21 Control: n=17	BF	“Any” BF (MA) BF duration (MA)	2‐4 months, 6‐9 months, and 12 months after birth
de Mello Sa et al (2025) [68], RCT, United States	mHealth Nondigital usual care control	Expectant mothers (32‐ to 36-week gestation) within the study hospital Intervention: n=28 Control: n=34	BF BF self-efficacy Adverse events	EBF (MA) “Any” BF duration (MA) BF self-efficacy scale—short form (MA) Adverse events (summary)	6 months after birth 12 months after birth 12 months after birth 12 months after birth
Doan et al (2022) [69], RCT, Vietnam	mHealth Attention control	Expectant mothers (24‐ to 36-week gestation) planning on delivering by cesarean section Intervention: n=632 Control: n=634	BF	EBF (MA)	1, 4, and 6 months after birth
Fan et al (2022) [70], RCT, China	mHealth Nondigital usual care control	Primiparous women of term infants (37‐ to 42-week gestation) Intervention: n=15 Control: n=18	BF BF self-efficacy	EBF (MA) “Any” BF (MA) BF self-efficacy scale—short form (MA)	1, 2, 4, and 6 months after birth 1, 2, 4, and 6 months after birth Baseline and 2 months after birth
Fiks et al (2017) [71], RCT, United States	Computer-based eHealth Attention control	Expectant mothers (20‐ to 32-week gestation, BMI≥25 kg/m^2^) Intervention: n=43 Control: n=44	BF	EBF (MA)	6 months after birth
Gilano et al (2025) [72], cluster RCT, Ethiopia	mHealth Nondigital usual care control	Expectant mothers at the end of their second trimester Intervention: n=337 Control: n=338	BF	EBF (MA)	6 months after birth
Gonzalez-Darias et al (2020) [73], RCT, Spain	Computer-based eHealth Nondigital usual care control	Primiparous mothers of newborns Intervention: n=76 Control: n=78	BF	EBF (MA) “Any” BF (MA)	3 and 6 months after birth
Grijalva Eternod et al (2023) [74], cluster RCT (2×2 factorial), Somalia	mHealth Attention control	Households of children aged 0‐59 months Intervention: n=404 Control: n=370	BF	EBF (summary)	3 and 9 months after baseline
Harari et al (2018) [75], RCT, United States	mHealth+telehealth Nondigital usual care control	Expectant mothers (18‐ to 30-week gestation) from local Supplemental Nutrition Program for Women, Infants and Children sites Intervention: n=32 Control: n=26	BF	EBF (MA)	2 weeks after birth
Hmone et al (2023) [76], RCT, Myanmar	mHealth Attention control	Expectant mothers (28‐ to 34-week gestation) Intervention: n=179 Control: n=174	BF BF self-efficacy Adverse events	EBF (MA) “Any” BF (MA) BF self-efficacy scale—short form (MA) Adverse events (summary)	1‐6 months after birth 1‐6 months after birth 1, 3, and 5 months after birth 1‐6 months after birth
Huang et al (2024) [77], RCT, China	mHealth Nondigital usual care control	Expectant couples with a first pregnancy ≥28-week gestation Intervention: n=48 Control: n=48	BF	EBF (MA) “Any” BF (MA) EBF duration (MA)	1 month and 6 months after birth
Johnston et al (2025) [78], cluster RCT, India	mHealth Nondigital usual care control	Mothers and family caregivers of newborns (fathers and grandmothers) Intervention: n=6841 Control: n=6650	BF	EBF (MA) “Any” BF (MA)	8 weeks after birth
LeFevre et al (2022) [79], RCT, India	Telehealth Nonintervention control	Expectant mothers (12‐ to 34-week gestation) and their partners Intervention: n=2695 Control: n=2400	BF	EBF (MA) “Any” BF (MA)	0‐6 months 12 months after birth
Li et al (2024) [80], cluster RCT, China	mHealth Nondigital usual care control	Primary caregivers of 0‐ to 3-year-old children (including mothers, fathers, grandparents, and other caregivers including nannies) Intervention: n=746 Control: n=586	BF	EBF (MA) “Any” BF (MA)	1 month post 9-month intervention; children <6 months and EBF at baseline eligible 1 month post 9-month intervention; children <12 months and BF at baseline eligible
Martinez-Brockman et al (2018) [81], RCT^a^, United States	mHealth Nondigital usual care control	Expectant mothers (<28-week gestation) part of the BF peer counseling program Intervention: n=114 Control: n=98	BF	EBF (MA) “Any” BF (MA)	2 weeks and 3 months after birth
Maslowsky et al (2016) [82], RCT, Ecuador	Telehealth Nondigital usual care control	Mothers of newborns at a large public hospital Intervention: n=102 Control: n=76	BF	EBF (MA)	3 months after birth
Miremberg et al (2022) [83], RCT, Israel	mHealth+telehealth Nondigital usual care control	Mothers of newborns Intervention: n=92 Control: n=100	BF	“Any” BF (MA)	2 weeks, 6 weeks, 3 months, and 6 months after birth
Mukunya et al (2025) [84], cluster RCT, Uganda	mHealth Nondigital usual care control	Expectant mothers >28-week gestation within study villages Intervention: n=995 Control: n=882	BF	EBF (MA)	28 days after birth
Musiimenta et al (2022) [85], RCT, Uganda	mHealth+uHealth Nondigital usual care control	Expectant mothers (first or second trimester) in antenatal care Intervention: n=40 Control: n=40	BF	EBF (MA)	6 weeks after birth
Ogaji et al (2021) [86], RCT, Nigeria	Telehealth Nondigital usual care control	Mothers of newborns who birthed in the study hospital and intended to BF Intervention: n=75 Control n=75	BF Adverse events	EBF (MA) Adverse events (summary)	1, 2, 3, 4, 5, and 6 months after birth
Sevda and Sevil (2023) [87], RCT, Turkey	mHealth Nondigital usual care control	Primiparous mothers of newborns birthed in the study hospital Intervention: n=75 Control: n=75	BF	EBF (MA)	7 days, 15 days, 1, 2, 4, and 6 months posthospital discharge
Patel et al (2018) [88], cluster RCT, India	Telehealth+mHealth Nondigital usual care control	Expectant mothers (32‐ to 36-week gestation) receiving antenatal care from study hospitals Intervention: n=517 Control: n=518	BF Cost or cost-effectiveness	EBF (MA) Cost incurred (summary) Incremental cost-effectiveness (summary)	6, 10, and 14 weeks, and 6 months after birth
Raj et al (2025) [89], RCT, India	mHealth Nondigital usual care control	Expectant multigravida mothers attending the study clinic Intervention: n=72 Control: n=72	BF	EBF (MA) BF self-efficacy scale—short form (MA)	2, 4, and 6 months after the intervention Immediately after the intervention and 48 hours after delivery
Sari and Altay (2020) [90], RCT, Turkey	Computer-based eHealth Nondigital usual care control	Primiparous expectant women (27‐ to 32-week gestation) attending the study clinic Intervention: n=44 Control: n=44	BF	EBF (MA)	1 week and 3 months after birth
Saucedo Baza et al (2023) [91], RCT, United States	mHealth Nondigital usual care control	Mothers of newborns birthed within the study hospital Intervention: n=20 Control: n=20	BF BF self-efficacy	EBF (MA) BF duration (MA) BF self-efficacy scale—short form (MA)	4‐6 weeks after birth
Schwarz et al (2024) [92], RCT, United States	Telehealth Nondigital usual care control	Expectant mothers >28-week gestation Intervention: n=205 Control: n=206	BF	EBF (MA) Any BF	6 months after birth 12 months after birth
Scott et al (2021) [93], 4-arm RCT, Australia	mHealth Nondigital usual care control	Fathers of expectant couples (32-week gestation) receiving antenatal care at the study hospital Intervention: app only n=397 and app plus education n=333 Control: n=358	BF BF self-efficacy	EBF (MA) “Any” BF (MA) BF self-efficacy scale—short form (MA)	6, 12, 18, and 26 weeks after birth 6, 12, 18, and 26 weeks after birth 6 weeks after birth
Tizvir et al (2024) [94], cluster RCT, Iran	mHealth+telehealth Nonintervention control	Mothers EBF an infant <6 months, attending study health centers Intervention: n=111 Control: n=113	BF	“Any” BF (MA)	Immediately after the intervention, 3 and 6 months after the intervention
Unger et al (2018) [95], 3-arm RCT, Kenya	mHealth Nondigital usual care control	Expectant mothers (<36-week gestation) attending antenatal care Intervention: one-way n=99 and two-way n=99 Control: n=100	BF Adverse events	EBF (MA) Adverse events (summary)	10, 16, and 24 weeks after birth
Vila-Candel et al (2024) [96], RCT, Spain	mHealth+uHealth Nondigital usual care control	Expectant mothers receiving antenatal care at study clinics Intervention: n=136 Control: n=134	BF	“Any” BF (MA)	15 days, 6 weeks, 3 months, and 6 months after birth
Wen et al (2020) [97], 3-arm RCT, Australia	mHealth+telehealth Attention control	Expectant mothers (24‐ to 34-week gestation) Intervention: telephone n=386 and text message n=384 Control: n=385	BF	EBF (MA) “Any” BF (MA)	6 months after birth 6 and 12 months after birth
Wong and Chien (2023) [98], RCT, China	Computer-based eHealth+telehealth Nondigital usual care control	Primiparous mothers to newborns Intervention: n=20 Control: n=20	BF BF self-efficacy	EBF (MA) “Any” BF (MA) EBF duration (MA) BF self-efficacy scale—short form (MA)	2 months after birth
Wu et al (2020) [99], RCT, China	mHealth Nondigital usual care control	Expectant mothers (>37-week gestation) Intervention: n=170 Control: n=174	BF	EBF (MA) “Any” BF (MA)	0‐1 months, 2‐3 months, and 4‐5 months after birth

a RCT: randomized controlled trial.

b BF: breastfeeding.

c EBF: exclusive breastfeeding.

d MA: meta-analysis.

e mHealth: mobile health.

In total, 11 of the included studies were conducted within the United States [56,59,61,62,67,68,71,75,81,91,92], 7 studies were conducted in Turkey [55,58,60,63,65,87,90], 5 in China [70,77,80,98,99], 4 in India [78,79,88,89], 3 in Iran [57,66,94], and 2 studies each were conducted in Australia [93,97], Spain [73,96], and Uganda [84,85]. The remainder of studies were conducted across a broad cross-section of countries, with 1 each conducted in Brazil [64], Canada [54], Ecuador [82], Ethiopia [72], Israel [83], Kenya [95], Myanmar [76], Nigeria [86], Somalia [74], and Vietnam [69].

### Trial Design Characteristics

In total, 35 of the included studies were RCTs [54-65,67-71,73,75-77,79,81-83,85-87,89-92,96,98,99], 4 were multiple intervention arm RCTs [66,93,95,97], and 7 were cluster RCTs [72,74,78,80,84,88,94]. Trial populations ranged in size from 33 [70] to 13,491 participants [78].

### Participants

The majority (38/46) of the included studies targeted only mothers, recruited either prenatally (n=23) [57,59,60,62,63,68-72,75,76,81,84,85,88-90,92,95-97,99] or postnatally (n=15) [55,56,58,61,64-66,73,82,83,86,87,91,94,98]. In total, 7 of the studies targeted both parents [54,67,74,77-80], including 2 that targeted households (eg, primary caregivers of young children broadly) [74,80]. Only 1 intervention was “father-focused” [93].

### Interventions

Included interventions were summarized in Multimedia Appendix 2 [54-99], as per the TIDieR checklist [44]. Most (35/46) of the included studies had a focus on improving breastfeeding behaviors. The remaining 11 studies included breastfeeding content but had a focus on maternal and infant health more broadly [61,74,76,79,80,82-85,95,97]. In total, 25 of the interventions were delivered using mHealth (eg, messages or support delivered via mobile or smartphone) [55,57-59,61,62,67-70,72,74,76-78,80,81,84,85,87,89,93,95,96,99], 8 were delivered via computer-based eHealth [54,56,63,64,71,73,90,91], and 4 were delivered via telehealth [79,82,86,92]. A further 7 interventions combined mHealth and telehealth strategies [65,66,75,83,88,94,97], and 2 combined computer-based eHealth and telehealth strategies [60,98]. Interventions were delivered for a duration ranging from 1 week (posthospital discharge [66]) up to 12 months post partum [67,68,79]. In total, 13 of the interventions were delivered via automated systems [61,62,67,69,74,76,79,85,90,91,93,96,99], while the remaining 33 interventions contained some elements that required delivery via trained or qualified personnel (including peer counselors [70,73,75,81,84], research staff [54,55,57,58,60,63,65,66,72,87], academics [64], psychologists [71], nurses [82,95,97], lactation specialists [56,77,83,88,98], gynecologists [59], and pediatricians [86,89,94]).

In total, 11 of the studies indicated that the intervention content or information was underpinned by a theory or framework, the most common being the health belief model (n=3) [67,76,97] and the theory of planned behavior (n=3) [62,75,94]. Other frameworks and models included (1) breastfeeding coparenting framework [54], (2) self-regulation model [56], (3) social cognitive theory [62], (4) social learning theory [71], (5) health action process approach to behavior change [81], (6) Pender’s health promotion model [90], (7) Dennis’ breastfeeding self-efficacy framework [57,98], and (8) behavior change communications framework [72]. Coding to the SEM showed that all interventions targeted individual factors (eg, knowledge, self-efficacy, motivation, and skills), 17 also included components targeting interpersonal factors (eg, coparent support) [54,62,64-66,69-71,73-75,78,81,84,93,94,98], and 20 included community factors (eg, linking to health services or professionals) [56,59-61,68,69,71-73,77,78,82,83,85,86,88,89,94,95,98]. A total of 21 of the interventions incorporated elements that were tailored, including (1) participant preferences for time or day of receiving content (n=1) [95], (2) participant preferred language (n=6) [62,74,78,81,88,96], (3) the age and stage of gestation and/or the infant (n=3) [76,85,99], (4) the delivery pathway (n=1) [61], and (5) based on specific questions or issues raised by the participant (n=11) [56,59,60,65,66,70,71,73,82,83,96].

### Comparisons

Included studies compared the intervention to either nondigital usual care control (n=35), attention control (n=9) [59,62,64,69,71,74,76,97], or nonintervention control (n=2) [63,79].

### Primary Outcomes

All measures of the primary outcome (EBF, any breastfeeding, and breastfeeding duration) were measured via self-reported methods. EBF was reported in 41 of the included studies [54-60,62-66,68-93,95,97-99], and “any” breastfeeding was reported in 22 studies [57,59,61,62,67,68,70,73,76-81,83,92-94,96-99] (Table 1).

A total of 39 studies were included in the EBF meta-analysis [54-60,62-65,68-73,75-79,81,82,84-93,95,97-99], 21 were included in the meta-analysis exploring “any” breastfeeding rates [57,59,61,62,67,70,73,76-81,83,92,93,96-99], and 7 were included in the meta-analysis for breastfeeding duration [64,66-68,77,91,98].

### Secondary Outcomes

Breastfeeding self-efficacy was reported in 14 included studies, 2 of these studies used the long-form version of the validated tool [62,66], while 12 used the short-form version [54,57,58,63,65,68,70,76,89,91,93,98] of the Breastfeeding Self-Efficacy Scale [100]. The long-form of the Breastfeeding Self-Efficacy Scale uses a 33-item tool for scoring breastfeeding self-efficacy [101], and the short-form includes 14 items [102]. In both cases, a higher score indicates higher levels of breastfeeding self-efficacy. As such, all 14 studies were included in the meta-analysis [54,57,58,62,63,65,66,68,70,76,89,91,93,98].

Patel et al [88] was the only included study to report on intervention costs (ie, costs incurred by the health care providers and patients) or cost-effectiveness (ie, incremental cost-effectiveness).

Adverse outcomes were mentioned in 5 of the included studies and related to adverse events as reported by study participants [62,68], adverse infant feeding practices [76], adverse clinical nutrition status [86], and serious adverse events as a result of the intervention [95].

### Study Risk of Bias in Included Studies

**Figure 2.**
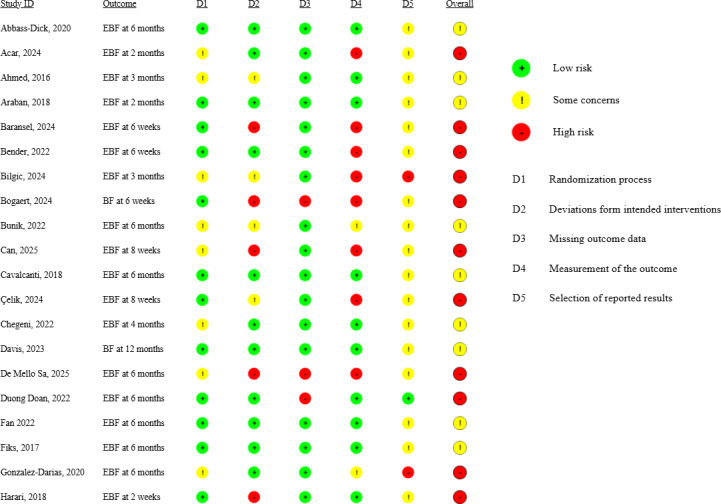
Risk of bias assessment of included randomized controlled trials using the Cochrane Risk of Bias 2 tool [54-71,73,75]. BF: breastfeeding; EBF: exclusive breastfeeding.

For the 39 RCTs, overall risk of bias was mostly assessed as having “some concerns” (n=15) [54,56,57,62,64,66,67,70,71,86,87,91,92,95,99] or “high risk” (n=20) [55,58-61,63,65,68,69,73,75,77,79,81-83,85,89,90,98] of bias. “Some concerns” for study risk of bias was most frequently indicated for domain 5 “the selection of reported results” due to the absence of a preregistered data analysis plan. “Some concerns” for study risk of bias was also frequently indicated for domain 1 the “randomization process,” due to the detection of baseline differences between intervention groups. “High risk” of bias was most frequently indicated for domain 2 “deviations from intended interventions” and domain 3 “missing outcome data,” whereby breastfeeding data were not available for “almost all” participants (Figures2  3).

For the 7 cluster RCTs, 1 study [74] was “low risk” of bias overall, and the other 6 were “high risk” of bias [72,78,80,84,88,94]. “High risk” of bias was most frequently indicated for domain 2 “deviations from the intended interventions” and domain 4 “measurement of the outcome” (Figure 4).

**Figure 3.**
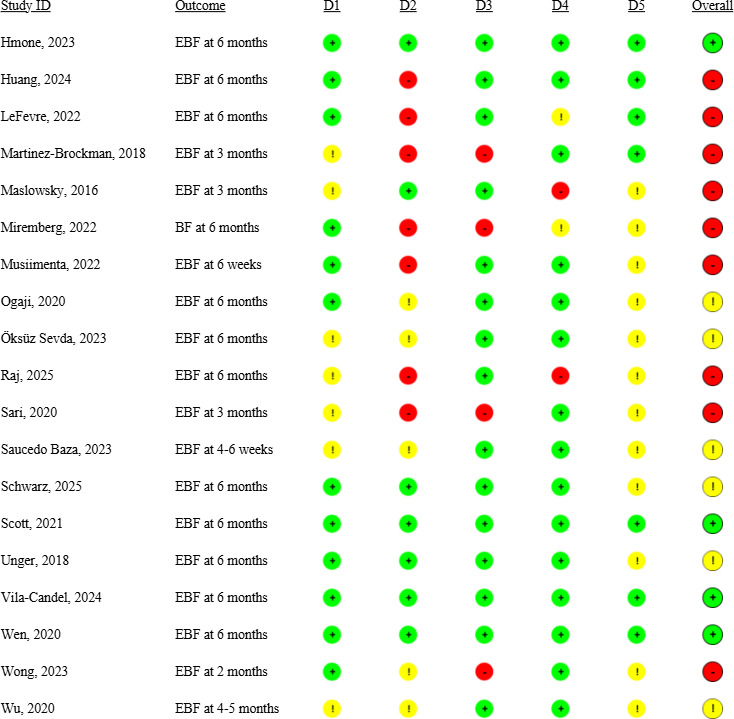
Risk of bias assessment of included randomized controlled trials using the Cochrane Risk of Bias 2 tool [76,77,79,81-83,85-87,89-93,95-99]. BF: breastfeeding; EBF: exclusive breastfeeding.

**Figure 4.**
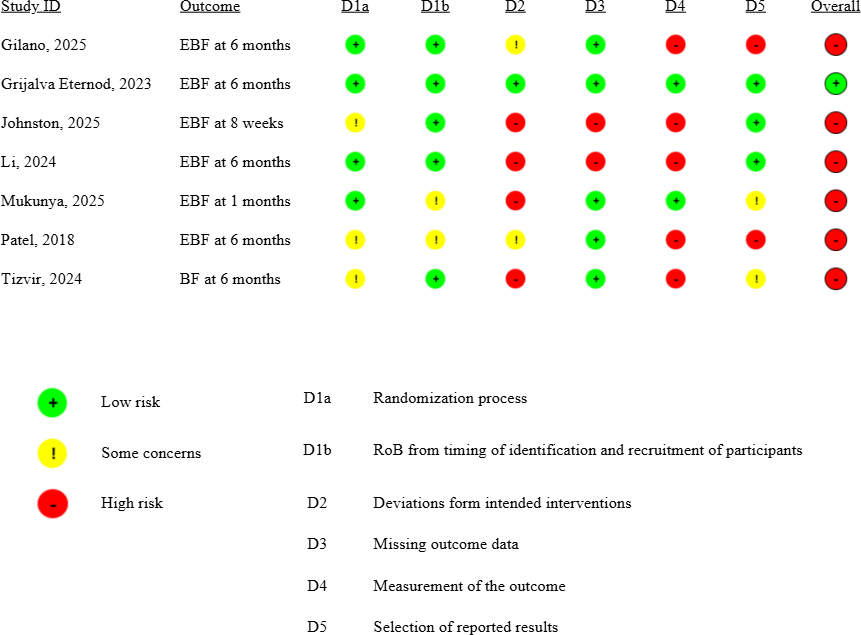
Risk of bias assessment of included cluster randomized controlled trials using the Cochrane RoB 2 tool, including cluster-specific bias domains [72,74,78,80,84,88,94]. BF: breastfeeding; EBF: exclusive breastfeeding; RoB: Risk of Bias.

### Results of Synthesis

#### Intervention Effects: Meta-Analysis

##### Exclusive Breastfeeding

**Figure 5.**
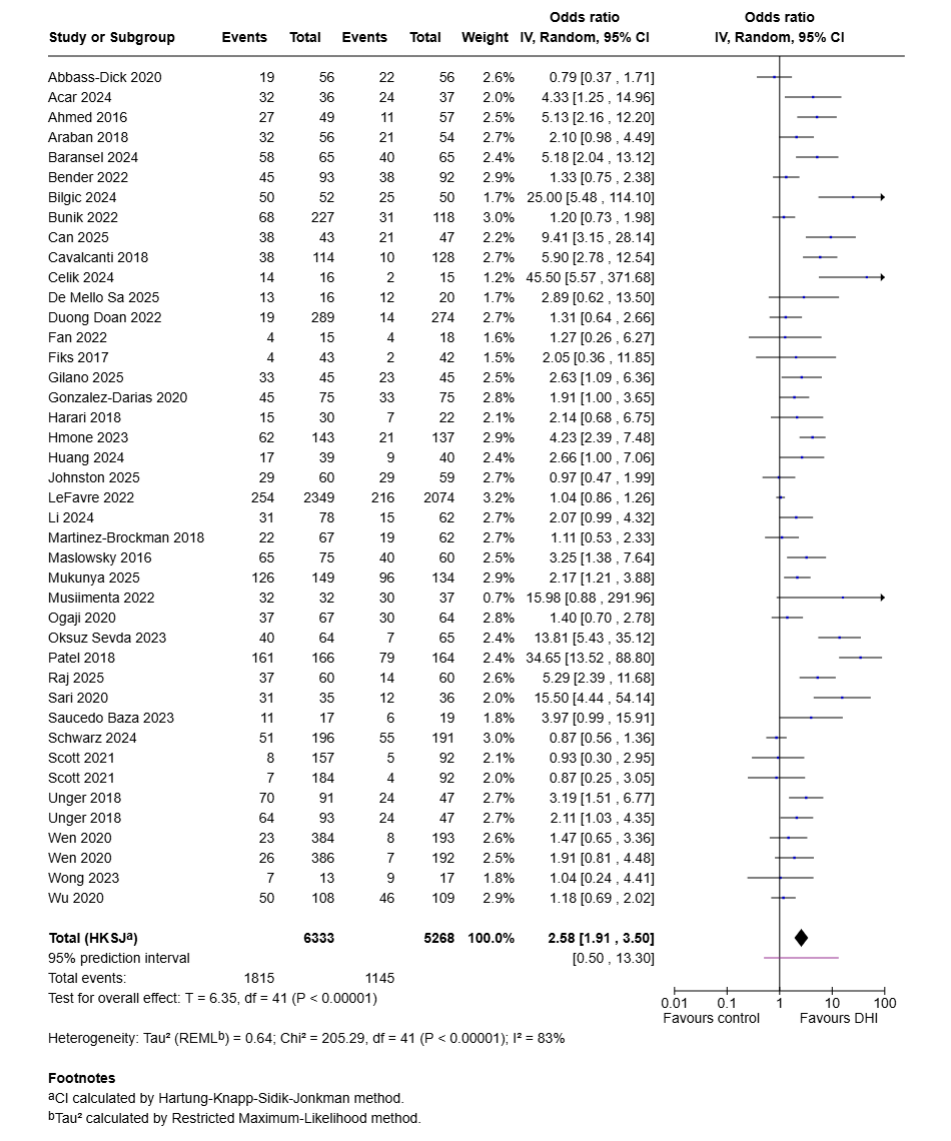
Meta-analysis: impact of DHIs on EBF [54-60,62-65,68-73,75-82,84-93,95,97-99]. Summary of pooled effects from 39 randomized controlled trials conducted across diverse geographic settings evaluating DHIs targeting mothers, fathers, and other caregivers compared with control conditions (eg, usual care, attention control, or no intervention) on EBF (n=11,601 participants). EBF was assessed between 1 week and 6 months post partum. Random-effects meta-analysis showed increased odds of EBF with DHIs (odds ratio 2.58, 95% CI 1.91‐3.50; *I*^2^=83%). DHI: digital health intervention; EBF: exclusive breastfeeding.

In total, 39 trials reported on the impact of a parent-targeted DHI on the prevalence of EBF and were included for meta-analysis (Figure 5). Relative to control, DHIs were shown to increase the odds of EBF (OR 2.58, 95% CI 1.91‐3.50; *I*^2^=83%; 39 trials, 11,601 participants); however, there was considerable evidence of heterogeneity. Results were similar in sensitivity analysis excluding trials at high risk of bias (OR 2.03, 95% CI 1.40‐2.96; *I*^2^=74%; 16 trials, 4171 participants; Figure S1 in Multimedia Appendix 3). Calculated prediction intervals indicate that 95% of studies comparable to those within this meta-analysis will sit within an OR of 0.50 and 13.30.

##### Subgroups

Subgroup analysis revealed that time to measurement of EBF status (ie, 6 months vs <6 months) did not modify the effect of the DHI (subgroup effect *P*=.24). There was, however, substantial unexplained heterogeneity between trials within each of these subgroups (eg, 6-month follow-up: *I*^2^=85% vs <6 months to follow-up: *I*^2^=77%; Figure S2 in Multimedia Appendix 3). Similarly, when the interventions were grouped based on prenatal support or postnatal only support, this was not shown to significantly modify the effect of the DHIs on EBF status (subgroup effect *P*=.15); yet, substantial unexplained heterogeneity between trial subgroups was present (eg, prenatal support: *I*^2^=85%; postnatal support: *I*^2^=70%; Figure S3 in Multimedia Appendix 3).

Studies grouped by type of digital modality (ie, computer-based eHealth vs mHealth vs telehealth vs mixed) indicated a subgroup effect (*P*=.02), suggesting that the treatment effect was greatest for the mixed modality interventions (OR 9.29, 95% CI 1.07‐80.60) followed by computer-based eHealth interventions (OR 3.78, 95% CI 1.45‐9.83), mHealth interventions (OR 2.16, 95% CI 1.63‐2.88), and then telehealth interventions (OR 1.33, 95% CI 0.73‐2.43; Figure S4 in Multimedia Appendix 3). A subgroup effect (*P*=.0002) was indicated if the DHI was directed at “mothers only” , suggesting that the “mother only” directed DHIs had a greater impact on EBF (OR 3.10, 95% CI 2.19‐4.38) than those directed at “parents/carers” more generally (OR 1.29, 95% CI 0.89‐1.88; Figure S5 in Multimedia Appendix 3). However, there are only a small number of studies included in the “parents/carers” subgroup (n=7), limiting the ability to detect an effect, and high levels of heterogeneity were indicated across the “mother only” subgroup (*I*^2^=80%). Subgroup analysis based on study country income demonstrated a significant subgroup effect (*P*=.0007), suggesting that the treatment effect was greater for the “upper middle income” countries (OR 4.35, 95% CI 2.42‐7.83) and “low or lower middle income” countries (OR 2.73, 95% CI 1.43‐5.21) than the “high income” countries (OR 1.45, 95% CI 1.08‐1.94; Figure S6 in Multimedia Appendix 3). However, heterogeneity was lowest in the “high income” subgroup (*I*^2^=38%) compared to “upper middle income” (*I*^2^=75%) and “low or lower middle income” (*I*^2^=89%).

##### Any Breastfeeding

**Figure 6.**
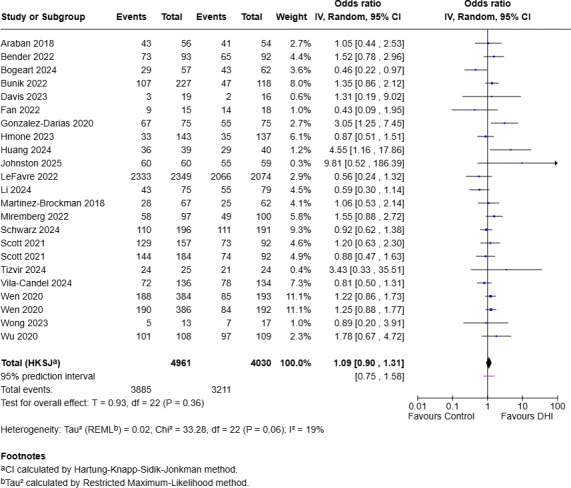
Meta-analysis: impact of DHIs on any breastfeeding [57,59,61,62,67,70,73,76-81,83,92-94,96-99]. Summary of pooled effects from 21 randomized controlled trials conducted across diverse geographic settings evaluating DHIs targeting mothers, fathers, and other caregivers compared with control conditions (eg, usual care, attention control, or no intervention) on any breastfeeding (n=8991 participants). Any breastfeeding was assessed up to 12 months post partum. Random-effects meta-analysis showed no significant effect of DHIs on any breastfeeding (odds ratio 1.09, 95% CI 0.90‐1.31; *I*^2^=19%). DHI: digital health intervention.

In total, 21 trials reported on the impact of a parent-targeted DHI on the prevalence of “any” breastfeeding and were able to be included in a meta-analysis (Figure 6). Relative to control, DHIs did not influence the prevalence of “any” breastfeeding (OR 1.09, 95% CI 0.90‐1.31; *I*^2^=19%; 21 trials, 8991 participants). Calculated prediction intervals indicated that 95% of studies comparable to those in the analysis will have an effect that will sit within an OR of 0.75 and 1.58. Sensitivity analysis excluding trials at high risk of bias indicated similar effect sizes and eliminated study heterogeneity (OR 1.08, 95% CI 0.93‐1.26; *I*^2^=0%; 10 trials, 3357 participants; Figure S1 in Multimedia Appendix 4). Further subgroup analyses did not indicate that factors including time to outcome measurement, timing of support (ie, pre- or postnatal), DHI modality, participant type, or country income level were modifying or influencing study heterogeneity (Figures S1-S6 in Multimedia Appendix 4).

##### Breastfeeding Duration

**Figure 7.**
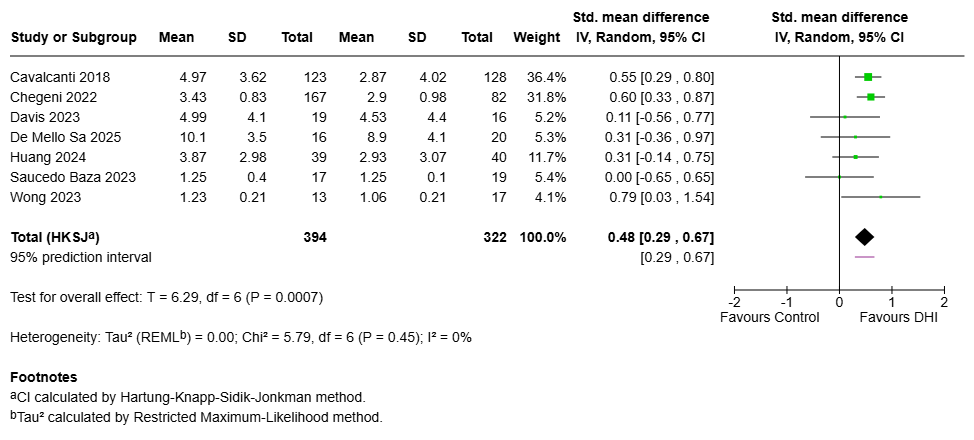
Meta-analysis: impact of DHIs on breastfeeding duration [64,66-68,77,91,98]. Summary of pooled effects from 7 randomized controlled trials conducted across diverse geographic settings evaluating DHIs targeting parents compared with control conditions (eg, usual care or attention control) on breastfeeding duration (n=716 participants). Random-effects meta-analysis showed increased breastfeeding duration with DHIs (standardized mean difference 0.48, 95% CI 0.29‐0.67; *I*^2^=0%). DHI: digital health intervention.

In total, 7 trials reported breastfeeding duration data that were able to be combined in a meta-analysis (Figure 7). Relative to control, the use of parent-targeted DHIs resulted in increased breastfeeding durations (SMD 0.48, 95% CI 0.29‐0.67; *I*^2^=0%; 7 trials, 716 participants). Calculated 95% prediction intervals suggest that comparable studies could expect to produce effects that sit between an SMD of 0.29 and 0.67. Sensitivity analysis excluding the high risk of bias studies indicated similar effect sizes (SMD 0.50, 95% CI 0.17‐0.83; *I*^2^=0%; 4 trials, 571 participants).

##### Breastfeeding Self-Efficacy

**Figure 8.**
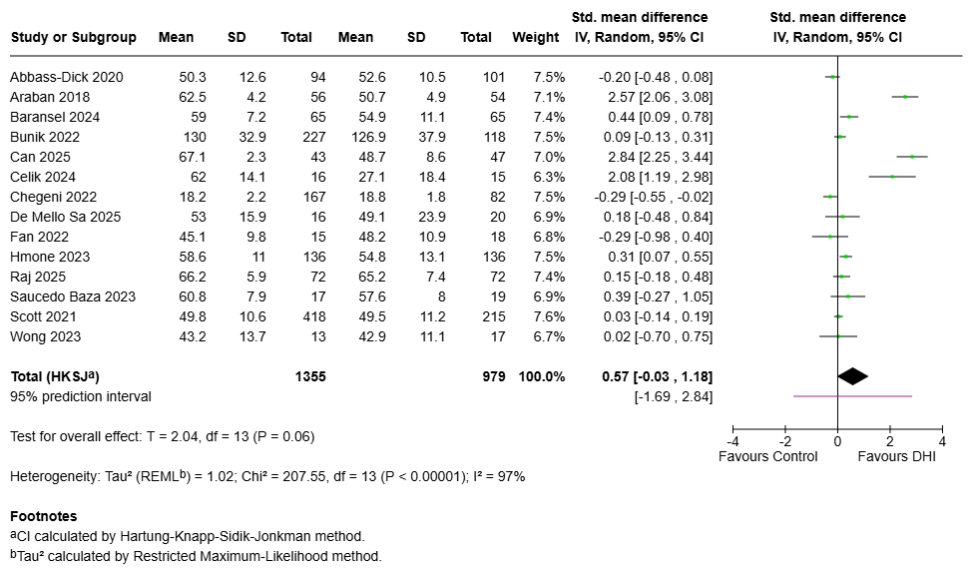
Meta-analysis: impact of DHIs on breastfeeding self-efficacy [54,57,58,62,63,65,66,68,70,76,89,91,93,98]. Summary of pooled effects from 14 randomized controlled trials conducted across diverse geographic settings evaluating DHIs targeting mothers, fathers, and other caregivers compared with control conditions (eg, usual care, attention control, or no intervention) on breastfeeding self-efficacy (n=2334 participants). Random-effects meta-analysis showed improved breastfeeding self-efficacy with DHIs (standardized mean difference 0.57, 95% CI −0.03 to 1.18; *I*^2^=97%). DHI: digital health intervention.

In total, 14 included trials reported on breastfeeding self-efficacy scores that were combined in a meta-analysis (Figure 8). Relative to control, the parent-targeted DHIs had no effect on breastfeeding self-efficacy scores (SMD 0.57, 95% CI −0.03 to 1.18; *I*^2^=97%; 14 trials, 2334 participants), with prediction intervals suggesting that comparable studies could expect effects to sit within an SMD of −1.69 to 2.84. Sensitivity analysis whereby high risk of bias studies were removed from the analysis reduced the impact of DHIs on breastfeeding self-efficacy scores but did not account for high levels of heterogeneity (SMD 0.32, 95% CI −0.46 to 1.09; *I*^2^=97%; 8 trials, 1873 participants).

##### Certainty of Evidence

GRADE assessments for primary outcomes explored in meta-analyses are presented in Table 2. Evidence for EBF was rated as very low certainty, downgraded for risk of bias and inconsistency (ie, heterogeneity), and suspected small study effects and publication bias (Figure S1 in Multimedia Appendix 5). Thus, while evidence indicates that parent-targeted DHIs may increase EBF, the evidence is very uncertain. Evidence for any breastfeeding was also rated as very low certainty, downgraded for risk of bias, and suspected small study effects and publication bias (Figure S2 in Multimedia Appendix 5), indicating that while parent-targeted DHIs have no effect on any breastfeeding, the evidence is very uncertain. Evidence for breastfeeding duration was rated as moderate and downgraded only for risk of bias, suggesting that parent-targeted DHIs likely increase breastfeeding duration.

**Table 2.** GRADE^a^ assessment.

Certainty assessment						Participants		Effect		Certainty	What happens
Study design (studies, n)	RoB^b^	Inconsistency	Indirectness	Imprecision	Other	DHI^c^, n/N (%)	Control, n/N (%)	Relative, OR^d^ (95% CI)	Absolute (95% CI)		
Exclusive breastfeeding (EBF)											
RCT^e^ (39)	Very serious^f^	Very serious^g^	Not serious	Not serious	Small study effects and publication bias suspected^h^	1815/6333 (28.7)	1145/5268 (21.7)	2.58 (1.91-3.50)	200 more per 1000 (from 129 more to 276 more)	⨁◯◯◯ Very low	Parent-targeted DHI may increase EBF, but the evidence is very uncertain.
Any breastfeeding											
RCT (21)	Very serious^i^	Not serious	Not serious	Not serious	Small study effects and publication bias suspected^j^	3885/4961 (78.3)	3211/4030 (79.7)	1.09 (0.90-1.31)	14 more per 1000 (from 18 fewer to 40 more)	⨁◯◯◯ Very low	Parent-targeted DHI has no effect on any breastfeeding, but the evidence is very uncertain.
Breastfeeding duration											
RCT (7)	Very serious^k^	Not serious	Not serious	Not serious	None^l^	394^m^	322^m^	—^n^	SMD^o^ 0.48 SD Higher (0.29 higher to 0.66 higher)	⨁⨁⨁◯ Moderate	Parent-targeted DHI likely increases breastfeeding duration.

a GRADE: Grading of Recommendations Assessment, Development, and Evaluation.

b RoB: Risk of Bias.

c DHI: digital health intervention.

d OR: odds ratio.

e RCT: randomized controlled trial.

f In total, 23 studies are at high risk of bias, and a further 12 are of some concerns.

g Heterogeneity across studies is 83% and unable to adequately explain heterogeneity with subgroup analyses.

h In total, 11 studies are at high risk of bias, and a further 6 are of some concerns.

i Indicated via funnel plot inspection (Figure S1 in Multimedia Appendix 5).

j Indicated via funnel plot inspection (Figure S2 in Multimedia Appendix 5).

k In total, 3 studies are at high risk of bias, and a further 4 studies are of some concerns.

l Indicated via funnel plot inspection (Figure S3 in Multimedia Appendix 5).

m n values are presented for continous variable.

n Not applicable.

o SMD: standardized mean difference.

### Intervention Effects: Summary

#### Exclusive Breastfeeding

One 2×2 factorial cluster RCT by Grijalva-Eternod et al [74] provided breastfeeding data that were unable to be combined in a meta-analysis. The study reported no effect of the mHealth intervention on EBF for infants younger than 6 months of age (adjusted OR 2.12, 95% CI 0.33‐13.71; *P*=.43).

#### Cost of Delivering the DHI

Patel et al [88] was the only study to report on relevant cost outcomes, including the health care costs incurred by the health care provider and patients and the incremental total cost of the intervention per percentage increase in EBF. The DHI delivered during 2010‐2012 via telephone calls was estimated to cost on average US $69.04, while control was estimated to cost on average US $37.28. The incremental cost-effectiveness ratio was calculated at US $63.65, given the 50% improvement in EBF rates indicated at 6 months in the intervention group compared with control [88].

#### Unintended Adverse Events

Compared to a control, no unintended adverse effects of the included DHIs were identified in the 5 trials that reported assessing them (1330 participants) [62,68,76,86,95].

## Discussion

### Principal Findings

This systematic review and meta-analysis included a total of 39 RCTs and 7 cluster RCTs exploring the effect of parent-directed DHIs (inclusive of eHealth, mHealth, and telehealth interventions) on breastfeeding behaviors, spanning broad contexts and populations from high- to low-income countries. Using gold-standard systematic review methodology, this review provides global evidence exploring the impact of DHIs on key breastfeeding behaviors (ie, EBF and breastfeeding duration), addressing a timely and policy-relevant evidence gap. Our pooled findings indicate that the odds of EBF are approximately double for those receiving a DHI than control (OR 2.58, 95% CI 1.91-3.50; *P*<.00001); however, our certainty of this evidence is very low. Our pooled analysis also suggests with moderate certainty an effect for parent-targeted DHIs on breastfeeding duration compared with control (SMD 0.48; *P*=.0007), equivalent to an additional 2 weeks of breastfeeding.

The results from this review are aligned with previous reviews [27-29,103], indicating that parent-targeted DHIs are effective for improving breastfeeding behaviors, despite extending the literature search to include high-, middle-, and low-income countries, while examining a comprehensive range of modalities for DHI delivery. The results of this review are highly encouraging, given that small population-level increases in breastfeeding exclusivity and duration contribute significant and ongoing health benefits for both infants and mothers [104,105]. As such, this review provides evidence of very low to moderate certainty that there is a role for DHIs as part of the health care provided to parents during the first 2000 days as a potentially scalable strategy for improving breastfeeding behaviors [106].

While the meta-analysis for EBF demonstrated considerable heterogeneity (*I*^2^=83%), this was only slightly reduced when we excluded high risk of bias studies (*I*^2^=74%). Tests for subgroup differences suggested that while in all cases the DHI favored improved EBF rates compared with control, there was a statistically significant subgroup effect based on DHI type (*P*=.02), parent type targeted (*P*=.0002), and study country income level (*P*=.0007). These statistically significant subgroup effects indicated that a treatment effect was greatest for interventions that combined mHealth or eHealth strategies with telehealth, DHIs that targeted only mothers and were delivered within upper-middle-income countries. However, in all cases, there remained substantial unexplained heterogeneity within subgroups, suggesting that the validity of the treatment effect estimate for each subgroup is uncertain, as individual trial results remain inconsistent. While GRADE assessment of the EBF evidence suggests a very low level of certainty (influenced by high levels of heterogeneity and study risk of bias), the calculated 95% prediction intervals suggest that future studies exploring the impact of DHIs on EBF could reasonably expect effects ranging between moderately reduced odds (OR 0.50) due to DHI exposure to a very large increased odds (OR 13.30), demonstrating a very high potential for benefit. In addition, our meta-analysis exploring the impact of DHIs on breastfeeding duration was statistically significant with no heterogeneity present (SMD 0.48; *P*=.0007; *I*^2^=0%). Additionally, calculated 95% prediction intervals indicated that future studies could expect DHI exposure to increase breastfeeding durations between 1 and 3 weeks (SMD 0.29-0.66) with moderate certainty in the evidence, adding to the overall findings that parent-directed DHIs are effective at improving breastfeeding outcomes.

A recent systematic review and network meta-analysis by Fan et al [107] indicated that text messaging interventions that covered antenatal and postnatal periods and delivered weekly were most effective in improving EBF rates. Meanwhile, our meta-analysis of DHIs more broadly indicated that the odds of EBF were not significantly modified based on whether the DHI was delivered only during the postnatal period (OR 3.51; *P*=.0003) or commenced during the prenatal period (OR 2.25; *P*=.0001). This result should however be interpreted with some caution, given that the number of studies included in the postnatal engagement subgroup is much lower (n=13) compared with the prenatal engagement subgroup (n=26). Thus, several other factors could be driving this result such as the broad differences between DHIs in terms of intensity, dosage, and the inclusion of peer or health professional contact. Given the diversity of included DHIs within this review, quantifying the impact of these factors was not possible within the current scope of this review. However, DHIs that were assessed at a lower risk of bias and produced clear improvements to EBF compared with control contained some consistent design elements. These elements related to the use of evidence and theory in guiding intervention content and design [56,64,76,87,91,97]; the inclusion of actionable or motivational content addressing common breastfeeding challenges [56,76,87,95,97]; and the DHI content was provided alongside another nondigital element such as a physical booklet, face-to-face education sessions, and/or existing health care model [56,64,87,95,97].

As birthing parents globally continue to face barriers to optimal breastfeeding behaviors [108,109], DHIs demonstrate huge potential to positively transform breastfeeding support, as they uniquely offer a highly scalable model to directly reach consumers with relatively high fidelity [26]. While this review, along with previously published literature, demonstrates efficacy of DHIs for improving breastfeeding outcomes [27-29,103], we have identified an ongoing need to explore and publish the safety (eg, unintended adverse events) and cost-effectiveness of such innovations. As published data for these key scalability outcomes remain scarce across the published literature [110], we recommend future DHI research prioritize collecting and publishing safety and cost-effectiveness data to support the case for countries to invest in digital health care policy and infrastructure. A review of reviews by Tomori et al [111] suggests that there is a need to build on well-established knowledge to scale up breastfeeding protection, promotion, and support programs, suggesting that the literature as a whole disproportionately focused on high-income and upper-middle-income settings. Tomori et al [111] also suggest that while DHIs offer a promising opportunity for improving breastfeeding outcomes, better quality research design is necessary to ascertain the most effective types of DHIs. Our EBF meta-analysis included 13 RCTs conducted in high-income countries, 15 from upper-middle, and 11 from low or lower-middle-income countries, suggesting a fair spread of RCT evidence informing our findings, including a broad variety of cultural and health system contexts. Further, the subgroup analysis by country income indicated statistically significant differences across subgroups (*P*=.0007), with the greatest effect noted for upper-middle-income countries (OR 4.35), followed by low or lower-middle-income (OR 2.73) and high-income (OR 1.45); yet, the positive impact of DHIs on EBF was statistically significant for all subgroups. However, given some of the inherent limitations of DHIs including a reliance on personal technology and local network access, varied engagement levels, and health or technology literacy disparities [112,113], concerns remain for breastfeeding inequities for socioeconomically vulnerable populations and those who belong to discriminated ethnic or racial groups [114,115]. As such, future studies should prioritize exploring the reach and impact of DHIs across population subgroups to ensure that population-level scale-up and the expected breastfeeding benefits would reach priority populations.

### Strengths and Limitations

While the meta-analyses for breastfeeding duration indicated a consistent effect across individual DHI studies, with no heterogeneity (*I*^2^=0%), there was considerable unexplained heterogeneity present in the EBF meta-analysis. This heterogeneity is likely driven by the large number of included studies that carried differences in study risk of bias (eg, methodological heterogeneity) and differences in participants, types of DHIs, and outcome assessment (eg, clinical heterogeneity). For example, many studies included in the analysis (57%) were deemed to have a high risk of bias, diminishing the trustworthiness and generalizability of the EBF results, which has been reflected in our GRADE assessments. However, when we removed the high risk of bias studies, the meta-analysis indicated similar effect sizes and statistical significance.

Studies included within this review almost entirely relied on self-reported data to inform breastfeeding status despite previous research suggesting self-reported measures of EBF are likely overestimated due to parents’ misunderstanding of what constitutes EBF [116]. Further, varied definitions of what qualified EBF within studies were minimally provided and could also be influencing large variations. For included studies that reported on EBF and “any” breastfeeding across multiple time points, we included the data for the longest follow-up time point (ie, included 6 months data rather than 3-month data) within our meta-analyses. Given that there is an ongoing need to increase the proportion of infants EBF to 6 months and an increase in breastfeeding duration is most desirable [117], the inclusion of data to the longest point to follow-up aligns best with recommended breastfeeding behaviors. However, it is possible that this approach is underestimating the impact of some DHIs included within the review.

Higher breastfeeding self-efficacy scores are consistently linked with better breastfeeding outcomes [118]. Our meta-analysis suggests that parent-targeted DHIs have no impact on breastfeeding self-efficacy. However, given our meta-analysis only included RCTs that reported on a breastfeeding outcome aligned with our primary outcomes (as per systematic review inclusion criteria), this analysis is likely missing key studies to inform the impact of DHIs on breastfeeding self-efficacy; thus, the true impact of DHIs on this outcome could be underestimated and should be taken with caution. Additionally, consistent with previous systematic reviews of this literature [103], the use of “usual care control” groups likely contributed a major source of inconsistency between studies. This review included RCTs that targeted parents and primary caregivers generally, which is an extension from previous reviews that focused exclusively on mothers [103]. While the subgroup analysis of EBF indicated that DHIs targeting parents generally (ie, fathers or nonbirthing coparents) were not effective (OR 1.29), this subgroup included only 7 studies; thus, further research exploring this relationship could be beneficial, given fathers or nonbirthing coparents and caregivers are considered a key source of social support for mothers [119], and seems an emerging field of exploration.

Strengths of this systematic review and meta-analysis include the use of robust research methodology aligned with best practice Cochrane methodology [31] and include high-quality research (RCTs), providing a high level of evidence for the effect of DHIs on breastfeeding behaviors. Further, this systematic review includes studies from a variety of countries internationally, addressing a key limitation of previous systematic reviews conducted on this topic [27].

### Conclusions

To the authors’ knowledge, this systematic review is the first to combine global RCT evidence spanning diverse cultural and health system contexts to comprehensively synthesize the impacts of parent-targeted DHIs (including eHealth, mHealth, and telehealth) on key breastfeeding outcomes. This review is innovative in that it has comprehensively combined data using best practice methodology to summarize evidence from a rapidly growing field, and to address an ongoing public health and policy priority. Given even minor improvements to EBF and breastfeeding duration are linked to significant societal and public health benefits [15], our findings indicate with very low to moderate certainty that parent-targeted DHIs may be an effective breastfeeding promotion strategy, specifically for EBF rates, and improved breastfeeding duration. It is important to highlight that the results of this review have been graded as moderate to very low certainty and should be interpreted cautiously, due to the presence of trial methodology biases, potential publication bias, and heterogeneity. Our findings indicate real-world implications for parent-targeted DHIs, suggesting that DHIs should cautiously be considered as a complementary strategy to support the usual health care provided to parents during the first 2000 days. Given the identified limitations in the quality and consistency of current evidence, future trials should seek to minimize possible biases by publishing comprehensive trial and data analysis protocols. Further, data pertaining to the potential adverse events, cost-effectiveness, and equity of DHIs are urgently needed to support justification for widescale implementation and policy investment.

## Supplementary material

10.2196/89214 Multimedia Appendix 1 Electronic database search terms.

10.2196/89214 Multimedia Appendix 2 Intervention details as per TIDieR checklist and the socioecological model.

10.2196/89214 Multimedia Appendix 3 Exclusive breastfeeding sensitivity and subgroup analyses.

10.2196/89214 Multimedia Appendix 4 Any breastfeeding sensitivity and subgroup analyses.

10.2196/89214 Multimedia Appendix 5 Funnel plots for Grading of Recommendations Assessment, Development, and Evaluation assessment.

10.2196/89214 Checklist 1 Expanded PRISMA checklist.

10.2196/89214 Checklist 2 PRISMA-S checklist.

10.2196/89214 Checklist 3 PRISMA-Abstract Checklist.

## Abbreviations

DHI: digital health intervention
EBF: exclusive breastfeeding
GRADE: Grading of Recommendations Assessment, Development, and Evaluation
mHealth: mobile health
OR: odds ratio
PRISMA: Preferred Reporting Items for Systematic Reviews and Meta-Analyses
PRISMA-S: Preferred Reporting Items for Systematic Reviews and Meta-Analyses-Searches
RCT: randomized controlled trial
SEM: socioecological model
SMD: standardized mean difference
TIDieR: Template for Intervention Description and Replication
