# Supplementary material for: The Effectiveness of Parent-Targeted Digital Health Interventions on Breastfeeding Practices: Systematic Review and Meta-Analysis of Randomized Controlled Trials
Source: J Med Internet Res. 2026 Jul 2;28:e89214. doi: 10.2196/89214 (PMC13326728; doi:10.2196/89214)
Supplement: Multimedia Appendix 1 [file jmir-v28-e89214-s001.docx]

**Electronic database search terms**

Cochrane Central Register of Controlled Trials (CENTRAL)

| **#** | **Searches** |
| --- | --- |
| 1 | [mh ^exercise] |
| 2 | [mh ^“physical conditioning, human”] |
| 3 | [mh ^“running”] |
| 4 | [mh ^swimming] |
| 5 | [mh ^“Motor Activity”] |
| 6 | [mh “Physical Fitness”] |
| 7 | [mh ^“life style”] |
| 8 | [mh ^“healthy lifestyle”] |
| 9 | [mh ^“healthy aging”] |
| 10 | [mh ^“diet, healthy”] |
| 11 | (physical NEXT (education or training)):ti,ab |
| 12 | ((lifestyle OR “life style”) NEAR/5 (activity OR program)):ti,ab |
| 13 | (“physical activity” OR exercise OR spor*):ti,ab |
| 14 | [mh Diet] |
| 15 | [mh ^“Child Nutrition Sciences”] |
| 16 | [mh ^Fruit] |
| 17 | [mh ^Vegetables] |
| 18 | [mh ^Eating] |
| 19 | [mh ^“Feeding Behaviour”] |
| 20 | [mh ^Food] |
| 21 | [mh ^“Carbonated Beverages”] |
| 22 | [mh ^“dietary fats”] |
| 23 | [mh ^“dietary fats, unsaturated”] |
| 24 | (nutrition OR “healthy eating” OR fruit OR vegetable OR calorie OR soda OR “soft drink” OR “sweet drink” OR confectionary):ti,ab |
| 25 | (energy NEXT (energy OR intake)):ti,ab |
| 26 | ((feeding OR eating OR food) NEXT (behavi*r OR habit)):ti,ab |
| 27 | [mh ^“Sedentary Behaviour”] |
| 28 | (sedentary NEXT (lifestyle OR behavi*)):ti,ab |
| 29 | (“physical inactivity”):ti,ab |
| 30 | [mh ^“Breast Feeding”] |
| 31 | (“breast feed” OR “breast fed” OR breastfeed OR breastfed):ti,ab |
| 32 | {or #1-#31} |
| 33 | [mh ^Parents] |
| 34 | [mh ^mothers] |
| 35 | [mh ^Pregnancy] |
| 36 | [mh ^“Postpartum Period”] |
| 37 | [mh ^Infant] |
| 38 | [mh ^“infant, newborn”] |
| 39 | (parent OR parent-targeted OR family OR infant OR newborn OR neonatal or child or baby or babies or “early child” Or maternal or mother):ti,ab,kw |
| 40 | ((Postnatal OR post-natal OR perinatal OR peri-natal OR postpartum OR post-partum) NEAR/2 (care OR service)):ti,ab |
| 41 | {or #33-#40} |
| 42 | [mh ^“Computers, handheld”] |
| 43 | [mh “Cell Phone”] |
| 44 | [mh ^“Mobile Applications”] |
| 45 | [mh ^“Virtual reality”] |
| 46 | [mh ^“Online Systems”] |
| 47 | [mh ^“Electronic mail”] |
| 48 | [mh ^telemedicine] |
| 49 | [mh ^“User-Computer Interface”] |
| 50 | [mh ^internet] |
| 51 | [mh ^“social media”] |
| 52 | (“mobile device” OR online OR internet OR email OR e-mail OR “world wide web” OR WWW OR website OR e-health OR ehealth OR “elctronic health” OR m-health OR mhealth OR “mobile health”):ti,ab |
| 53 | ((mobile OR cell OR smart) NEXT (phone OR telephone)):ti,ab |
| 54 | ((“digital health” OR digital OR mhealth) NEAR/3 intervention):ti,ab |
| 55 | ((interactive OR health OR communication) NEXT (video OR technology OR multimedia OR app)):ti,ab |
| 56 | ((phone OR mobile) NEXT app):ti,ab |
| 57 | {or #42-#56} |
| 58 | #32 and #41 and #57 |

CINAHL Complete

| **#** | **Searches** |
| --- | --- |
| 1 | (MH “Exercise”) |
| 2 | (MH “Running”) |
| 3 | (MH “Swimming”) |
| 4 | (MH “Walking”) |
| 5 | (MH “Motor Activity”) |
| 6 | (MH “Physical Education and Training”) |
| 7 | (MH “Physical Fitness”) |
| 8 | (MH “Life Style”) |
| 9 | “healthy aging” |
| 10 | (MH “Sports+”) |
| 11 | TI (“physical activit*” OR “physical fitness” OR exercis* OR sport* or “healthy lifestyle” or “healthy life style”) |
| 12 | AB(“physical activit*” OR “physical fitness” OR exercis* OR sport* or “healthy lifestyle” or “healthy life style”) |
| 13 | TI (Physical n1 (education or training)) |
| 14 | AB (Physical n1 (education or training)) |
| 15 | TI(Fitness n1 (class* or regime or program*)) |
| 16 | AB (Fitness n1 (class* or regime or program*)) |
| 17 | TI((Lifestyle or “life style”) n5 (activit* or program*)) |
| 18 | AB((Lifestyle or “life style”) n5 (activit* or program*)) |
| 19 | (MH “Diet+”) |
| 20 | (MH “Nutrition”) or ‘nutrition*” |
| 21 | TI “Healthy eating” or AB “Healthy eating” |
| 22 | “Child nutrition sciences” |
| 23 | TI fruit* or AB fruit* |
| 24 | (MH “Fruit”) |
| 25 | TI Vegetable* or AB Vegetable* |
| 26 | (MH “Vegetables”) |
| 27 | “calorie*” |
| 28 | (MH “Energy Intake”) |
| 29 | (MH “Eating”) |
| 30 | (MH “Eating Behaviour”) |
| 31 | (MH “Food”) |
| 32 | (MH “Food Habits”) |
| 33 | (MH “Carbonated Beverages”) |
| 34 | TI “soft drink” or AB “soft drink” |
| 35 | TI soda or AB soda |
| 36 | TI “sweetened drink*” or AB “sweetened drink*” |
| 37 | (MH “Dietary Fats”) |
| 38 | TI confectionar* or AB confectionar* |
| 39 | TI ( (( food or nutrition*) n1 program*)) OR AB (((food or nutrition*) n1 program*)) |
| 40 | (MH “Life Style, Sedentary”) |
| 41 | TI “physical inactivit*” or AB “Physical inactivit*” |
| 42 | TI (Sedentary n1 (lifestyle or behavio*)) OR AB (Sedentary n1 (lifestyle or behavio*)) |
| 43 | (MH “Breast Feeding”) |
| 44 | TI (“breast feed” or “breast fed” or breastfeed* or breastfed) |
| 45 | AB (“breast feed” or “breast fed” or breastfeed* or breastfed) |
| 46 | S1 OR S2 OR S3 OR S4 OR S5 OR S6 OR S7 OR S8 OR S9 OR S10 OR S11 OR S12 OR S13 OR S14 OR S15 OR S16 OR S17 OR S18 OR S19 OR S20 OR S21 OR S22 OR S23 OR S24 OR S25 OR S26 OR S27 OR S28 OR S29 OR S30 OR S31 OR S32 OR S33 OR S34 OR S35 OR S36 OR S37 OR S38 OR S39 OR S40 OR S41 OR S42 OR S43 OR S44 OR S45 |
| 47 | (MH “Parents”) |
| 48 | (MH “Mothers”) |
| 49 | (MH “Pregnancy”) |
| 50 | (MH “Postnatal Period”) |
| 51 | (MH “Infant”) |
| 52 | (MH “Infant, Newborn”) |
| 53 | TI (Pregnancy or parent* or parent-targeted or famil* or infant* or newborn* or neonat* or child or baby or babies or “early child*” or maternal or mother*) |
| 54 | AB (Pregnancy or parent* or parent-targeted or famil* or infant* or newborn* or neonat* or child or baby or babies or “early child*” or maternal or mother*) |
| 55 | TI ((Postnatal or post-natal or perinatal or peri-natal or postpartum or post-partum) n2 (care or service*)) |
| 56 | AB ((Postnatal or post-natal or perinatal or peri-natal or postpartum or post-partum) n2 (care or service*)) |
| 57 | S47 OR S48 OR S49 OR S50 OR S51 OR S52 OR S53 OR S54 OR S55 OR S56 |
| 58 | (MH “Computers, Hand-Held") |
| 59 | (MH “Cellular Phone”) or (MH “Smartphone”) |
| 60 | (MH “Mobile Applications”) |
| 61 | (MH “Virtual Reality”) or “Virtual reality” |
| 62 | (MH “Online Systems”) or “online” |
| 63 | (MH “Electronic Mail”) or “e-mail” or “email” |
| 64 | (MH “User-Computer Interface”) |
| 65 | (MH “internet”) or “internet” |
| 66 | TI “mobile device*” or AB “mobile device*” |
| 67 | TI (web n1 based) or AB (web n1 based) |
| 68 | TI((world wide web or www or website*)) or AB ((world wide web or www or website*)) |
| 69 | TI (((Mobile or cell* or smart n1 (phone* or telephone*))) or AB(((Mobile or cell* or smart n1 (phone* or telephone*))) |
| 70 | TI((e-health or ehealth or “electronic health”)) or AB((e-health or ehealth or “electronic health”)) |
| 71 | TI((“m-health” or mhealth or “mobile health”)) or AB((“m-health” or mhealth or “mobile health”)) |
| 72 | TI(((“digital health” or digital) n3 intervention*)) or AB (((“digital health” or digital) n3 intervention*)) |
| 73 | TI((interactive n1 ((health n1 communicat*) or video* or technolog* or multimedia))) or AB ((interactive n1 ((health n1 communicat*) or video* or technolog* or multimedia))) |
| 74 | S58 OR S59 OR S60 OR S61 OR S62 OR S63 OR S64 OR S65 OR S66 OR S67 OR S68 OR S69 OR S70 OR S71 OR S72 OR S73 |
| 75 | PT (“Randomized controlled trial” OR “Controlled clinical trial”) |
| 76 | S46 AND S57 AND S74 AND S75 |
| 77 | (MH “Animals”) NOT (MH “Human”) |
| 78 | S76 not S77 |

Education Research Complete

| **#** | **Searches** |
| --- | --- |
| 1 | “physical activit*” |
| 2 | “physical fitness” |
| 3 | exercis* |
| 4 | sport* |
| 5 | “healthy lifestyle” |
| 6 | “healthy life style” |
| 7 | (Physical W1 (education or training)) |
| 8 | (Fitness W1 (class* or regime or program*)) |
| 9 | ((lifestyle or “life style”) w5 (activit* or program*)) |
| 10 | diet |
| 11 | nutrition* |
| 12 | “healthy eating” |
| 13 | fruit* |
| 14 | vegetable* |
| 15 | calorie* |
| 16 | soda* |
| 17 | “soft drink*” |
| 18 | “sweet* drink*” |
| 19 | confectionar* |
| 20 | (Energy w1 (density or intake)) |
| 21 | ((feeding or eating or food) w1 (behavio* or habit*)) |
| 22 | (food w2 (purchas* or select*)) |
| 23 | “physical inactivit*” |
| 24 | (Sedentary w1 (lifestyle or behavio*)) |
| 25 | “breast feed*” |
| 26 | “breast fed” |
| 27 | breastfeed* or breastfed |
| 28 | S1 OR S2 OR S3 OR S4 OR S5 OR S6 OR S7 OR S8 OR S9 OR S10 OR S11 OR S12 OR S13 OR S14 OR S15 OR S16 OR S17 OR S18 OR S19 OR S20 OR S21 OR S22 OR S23 OR S24 OR S25 OR S26 OR S27 |
| 29 | Pregnancy |
| 30 | parent* |
| 31 | parent-targeted |
| 32 | famil* |
| 33 | infant* |
| 34 | newborn* |
| 35 | neonat* |
| 36 | child* |
| 37 | baby |
| 38 | “early child*” |
| 39 | maternal |
| 40 | mother* |
| 41 | ((Postnatal or post-natal or perinatal or peri-natal or postpartum or post-partum) w2 (care or service*)) |
| 42 | S29 OR S30 OR S31 OR S32 OR S33 OR S34 OR S35 OR S36 OR S37 OR S38 OR S39 OR S40 OR S41 |
| 43 | computer* |
| 44 | “mobile app” or “mobile apps” or “mobile application” |
| 45 | “mobile device*” |
| 46 | (online or internet) |
| 47 | (email* or e-mail*) |
| 48 | (“world wide web” or www or website) |
| 49 | (e-health or ehealth or “electronic health” |
| 50 | (m-health or mhealth or “mobile health”) |
| 51 | “mobile health” |
| 52 | “virtual reality” |
| 53 | (web-base* or webbase*) |
| 54 | ((Mobile or cell* or smart) w1 (Phone* or telephone*)) |
| 55 | TI ((“digital health” or digital or mhealth) w3 intervention*) |
| 56 | AB ((“digital health” or digital or mhealth) w3 intervention*) |
| 57 | TI ((interactive or health or communicat*) w1 (video* or technolog* or multimedia or app*)) |
| 58 | AB ((interactive or health or communicat*) w1 (video* or technolog* or multimedia or app*)) |
| 59 | TI (“Randomized controlled trial” or “controlled clinical trial” or randomized) |
| 60 | AB (“Randomized controlled trial” or “controlled clinical trial” or randomized) |
| 61 | S59 OR S60 |
| 62 | S43 OR S44 OR S45 OR S46 OR S47 OR S48 OR S49 OR S50 OR S51 OR S52 OR S53 OR S54 OR S55 OR S56 OR S57 OR S58 |
| 63 | S28 AND S42 AND S61 AND S62 |

Embase

| **#** | **Searches** |
| --- | --- |
| 1 | Exercise/ or physical education/ or swimming/ or walking/ or running/ |
| 2 | Motor Activity/ |
| 3 | Fitness/ |
| 4 | lifestyle/ or healthy lifestyle/ |
| 5 | healthy againg/ or healthy diet/ |
| 6 | (physical adj (education or training)).ti,ab,kw. |
| 7 | Exp Sport/ |
| 8 | Physical activity/ or physical* activ*.ti,ab,kw. |
| 9 | (exercis* or sport*).ti,ab,kw. |
| 10 | (fitness adj (class* or regime* or program*)).mp. |
| 11 | ((“lifestyle*” or life style*) adj5 (activit* or program*)).ti,ab,kw. |
| 12 | exp Diet/ |
| 13 | Child Nutrition Sciences.ti,ab,kw. |
| 14 | Fruit/ |
| 15 | Vegetable/ |
| 16 | Eating/ |
| 17 | Feeding Behaviour/ |
| 18 | Food/ |
| 19 | Carbonated Beverage/ |
| 20 | Fat intake/ or dietary intake/ |
| 21 | (nutrition* or healthy eating).ti,ab,kw. |
| 22 | (fruit* or vegetable*).ti,ab,kw. |
| 23 | Calorie*.ti,ab,kw. |
| 24 | (energy adj (density or intake)).ti,ab,kw. |
| 25 | ((feeding or eating or food) adj (behavio* or habit*)).ti,ab,kw. |
| 26 | (soda* or soft drink* or sweet* drink*).ti,ab,kw. |
| 27 | confectionar*.ti,ab,kw. |
| 28 | (food adj2 (purchas* or select*)).ti,ab,kw. |
| 29 | Sedentary lifestyle/ |
| 30 | sedentary.ti,ab,kw. |
| 31 | Physical* inactiv*.ti,ab,kw. |
| 32 | Breast Feeding/ |
| 33 | (breast feed* or breast fed or breastfeed* or breastfed).ti,ab,kw. |
| 34 | 1 or 2 or 3 or 4 or 5 or 6 or 7 or 8 or 9 or 10 or 11 or 12 or 13 or 14 or 15 or 16 or 17 or 18 or 19 or 20 or 21 or 22 or 23 or 24 or 25 or 26 or 27 or 28 or 29 or 30 or 31 or 32 or 33 |
| 35 | Parents/ or mothers/ |
| 36 | Pregnancy/ |
| 37 | Postpartum Period/ |
| 38 | Infant/ or newborn/ |
| 39 | (parent* or parent-targeted or famil*).ti,ab,kw. |
| 40 | (infant* or newborn* or neonat* or child* or baby or babies or early child*).ti,ab,kw. |
| 41 | (maternal or mother* or mom* or mum*).ti,ab,kw. |
| 42 | ((postnatal or post-natal or perinatal or peri-natal or postpartum or post-partum) adj2 (care or service*)).ti,ab,kw. |
| 43 | 35 or 36 or 37 or 38 or 39 or 40 or 41 or 42 |
| 44 | Smartphone/ or mobile phone/ or mobile device*.ti,ab,kw. |
| 45 | Mobile Application/ |
| 46 | Virtual reality/ or (virtual adj reality).ti,ab,kw. |
| 47 | Online Systems/ or online.ti,ab,kw. |
| 48 | e-mail/ |
| 49 | Computer interface/ |
| 50 | Internet/ |
| 51 | (online or internet or e-mail* or e-mail* or electronic mail).ti,ab,kw. |
| 52 | (world wide web or WWW or website*).ti,ab,kw. |
| 53 | (e-health or ehealth or electronic health).ti,ab,kw. |
| 54 | (m-health or mhealth or mobile health).ti,ab,kw. |
| 55 | (web adj based).ti,ab,kw. |
| 56 | ((mobile or cell* or smart) adj (phone* or telephone*)).ti,ab,kw. |
| 57 | ((digital health or digital or mhealth) adj3 intervention*).ti,ab,kw. |
| 58 | ((interactive or health or communicat*) adj (video* or technolog* or multimedia or app*)).ti,ab,kw. |
| 59 | ((chat adj room*) or chatroom*).ti,ab,kw. |
| 60 | ((phone or mobile) adj app*).ti,ab,kw. |
| 61 | 44 or 45 or 46 or 47 or 48 or 49 or 50 or 51 or 52 or 53 or 54 or 55 or 56 or 57 or 58 or 59 or 60 |
| 62 | exp randomized controlled trial/ |
| 63 | Controlled clinical trial/ |
| 64 | Random$.ti,ab. |
| 65 | Randomization/ |
| 66 | (compare or compared or comparison).ti,ab. |
| 67 | ((evaluated or evaluate or evaluating or assessed or assess) and (compare or compared or comparing or comparison)).mp. [mp=title, abstract, heading word, drug trade name, original title, device manufacturer, device trade name, keyword heading word, floating subheading word, candidate term word] |
| 68 | (assigned or allocated).ti,ab. |
| 69 | (controlled adj7 (study or design or trial)).ti,ab. |
| 70 | (volunteer or vounteers).ti,ab. |
| 71 | Human experiment/ |
| 72 | trial.ti. |
| 73 | 62 or 63 or 64 or 65 or 66 or 67 or 68 or 69 or 70 or 71 or 72 |
| 74 | 34 and 43 and 61 and 73 |
| 75 | Animal experiment/ not (human experiment/ or human/) |
| 76 | 74 not 75 |
| 77 | 62 or 63 or 64 or 65 or 72 |
| 78 | 34 and 43 and 61 and 77 |
| 79 | 78 not 75 |

MEDLINE

| **#** | **Searches** |
| --- | --- |
| 1 | Exercise/ or physical conditioning, human/ or swimming/ or walking/ or running/ |
| 2 | Motor Activity/ |
| 3 | exp Physical Fitness |
| 4 | life style/ or healthy lifestyle/ |
| 5 | healthy aging/ or diet, healthy/ |
| 6 | (physical adj (education or training)),ti,ab,kw. |
| 7 | exp Sports/ |
| 8 | physical* activ*.ti,ab,kw. |
| 9 | (exercise* or sport*).ti,ab,kw. |
| 10 | (fitness adj (class* or regime* or program*)).mp. |
| 11 | ((lifestyle or life style) adj5 (activit* or program*)).ti,ab,kw. |
| 12 | exp Diet/ |
| 13 | Child Nutrition Sciences/ |
| 14 | Fruit/ |
| 15 | Vegetables/ |
| 16 | Eating/ |
| 17 | Feeding Behaviour |
| 18 | Food/ |
| 19 | Carbonated Beverages |
| 20 | dietary fats/ or dietary fats, unsaturated/ |
| 21 | (nutrition* or healthy eating).ti,ab,kw. |
| 22 | (fruit* or vegetable*).ti,ab,kw. |
| 23 | Calorie*.ti,ab,kw. |
| 24 | (energy adj (density or intake)).ti,ab,kw. |
| 25 | ((feeding or eating or food) adj (behavio* or habit*)).ti,ab,kw. |
| 26 | (soda* or soft drink* or sweet* drink*).ti,ab,kw. |
| 27 | confectionar*.ti,ab,kw. |
| 28 | (food adj2 (purchas* or select*)).ti,ab,kw. |
| 29 | Sedentary Behaviour/ |
| 30 | (sedentary adj (lifestyle or behavio*)).ti,ab,kw. |
| 31 | Physical* inactiv*.ti,ab,kw. |
| 32 | Breast Feeding/ |
| 33 | (breast feed* or breast fed or breastfeed* or breastfed).ti,ab,kw. |
| 34 | 1 or 2 or 3 or 4 or 5 or 6 or 7 or 8 or 9 or 10 or 11 or 12 or 13 or 14 or 15 or 16 or 17 or 18 or 19 or 20 or 21 or 22 or 23 or 24 or 25 or 26 or 27 or 28 or 29 or 30 or 31 or 32 or 33 |
| 35 | Parents/ or mothers/ |
| 36 | Pregnancy/ |
| 37 | Postpartum Period |
| 38 | Infant/ or infant, newborn/ |
| 39 | (parent* or parent-targeted or famil*).ti,ab,kw. |
| 40 | (infant* or newborn* or neonat* or child* or baby or babies or early child*).ti,ab,kw. |
| 41 | (maternal or mother* or mom* or mum*).ti,ab,kw. |
| 42 | ((postnatal or post-natal or perinatal or peri-natal or postpartum or post-partum) adj2 (care or service*)).ti,ab,kw. |
| 43 | 35 or 36 or 37 or 38 or 39 or 40 or 41 or 42 |
| 44 | Smartphone/ or Cell Phone/ or mobile device*.ti,ab,kw. |
| 45 | Mobile Applications/ |
| 46 | Virtual reality/ or (virtual adj reality).ti,ab,kw. |
| 47 | Online Systems/ or online.ti,ab,kw. |
| 48 | Electronic mail/ or telemedicine/ |
| 49 | User-computer interface/ |
| 50 | Internet/ |
| 51 | (online or internet or email* or e-mail*).ti,ab,kw. |
| 52 | (world wide web or WWW or website*).ti,ab,kw. |
| 53 | (e-health or ehealth or electronic health).ti,ab,kw. |
| 54 | (m-health or mhealth or mobile health).ti,ab,kw. |
| 55 | (web adj based).ti,ab,kw. |
| 56 | ((mobile or cell* or smart) adj (phone* or telephone*)).ti,ab,kw. |
| 57 | ((digital health or digital or mhealth) adj3 intervention*).ti,ab,kw. |
| 58 | ((interactive or health or communicat*) adj (video* or technolog* or multimedia or app*)).ti,ab,kw. |
| 59 | ((chat adj room*) or chatroom*).ti,ab,kw. |
| 60 | ((phone or mobile) adj app*).ti,ab,kw. |
| 61 | 44 or 45 or 46 or 47 or 48 or 49 or 50 or 51 or 52 or 53 or 54 or 55 or 56 or 57 or 58 or 59 or 60 |
| 62 | exp randomized controlled trial/ |
| 63 | Controlled clinical trial.pt. |
| 64 | Randomized.ab. |
| 65 | Randomly.ab. |
| 66 | Trial.ab. |
| 67 | Groups.ab. |
| 68 | 62 or 63 or 64 or 65 or 66 or 67 |
| 69 | 34 and 43 and 61 and 68 |
| 70 | exp animals/ not humans.sh. |
| 71 | 69 not 70 |

Psycinfo

| **#** | **Searches** |
| --- | --- |
| 1 | exercise/ or physical activity/ |
| 2 | Physical Fitness/ |
| 3 | lifestyle/ or active living/ or lifestyle changes/ |
| 4 | (physical conditioning or running or walking).ti,ab,id. |
| 5 | (physical education and training).mp. |
| 6 | exp Sports/ |
| 7 | physical activ*.ti,ab,id. |
| 8 | (exercis* or sport*).mp. |
| 9 | (fitness adj (class* or regime or program*)).ti,ab,id. |
| 10 | ((“lifestyle*” or life style*) adj5 activ*).ti,ab,id. |
| 11 | Diets/ |
| 12 | Eating Behaviour/ |
| 13 | Food Intake/ |
| 14 | FOOD/ |
| 15 | (nutrition* or healthy eating).ti,ab,id. |
| 16 | (fruit* or vegetable*).ti,ab.id. |
| 17 | Calorie*.ti,ab,kw. |
| 18 | (energy intake or energy density).ti,ab,id. |
| 19 | (feeding behavio* or dietary intake or food habits).ti,ab,id. |
| 20 | (carbonated beverage* or soft drink* or soda or sweetened drink*).ti,ab,id. |
| 21 | confectionar*.ti,ab,id. |
| 22 | (food adj2 (purchas* or select)).ti,ab,kw. |
| 23 | Sedentary Behaviour/ |
| 24 | Sedentary.ti,ab,id. |
| 25 | Physical inactivit*.ti,ab,id. |
| 26 | Breast Feeding/ |
| 27 | (breast feed* or breast fed or breastfeed* or breastfed).ti,ab,id. |
| 28 | 1 or 2 or 3 or 4 or 5 or 6 or 7 or 8 or 9 or 10 or 11 or 12 or 13 or 14 or 15 or 16 or 17 or 18 or 19 or 20 or 21 or 22 or 23 or 24 or 25 or 26 or 27 |
| 29 | Parents/ or mothers/ |
| 30 | Pregnancy/ |
| 31 | Postnatal Period/ |
| 32 | (parent* or parent-targeted or famil*).ti,ab,id. |
| 33 | (infant* or newborn* or neonat* or child*or baby or babies or early child*).ti,ab,id. |
| 34 | (maternal or mother*).ti,ab,id. |
| 35 | ((postnatal or post-natal or perinatal or peri-natal or postpartum or post-partum) adj2 (care or service*)).ti,ab,id. |
| 36 | 29 or 30 or 31 or 32 or 33 or 34 or 35 |
| 37 | Computers/ or Computer Mediated Communication |
| 38 | exp Cellular Phones/ |
| 39 | exp Mobile Devices/ |
| 40 | Virtual reality/ or (virtual adj reality).ti,ab,id. |
| 41 | Electronic mail/ or telemedicine/ |
| 42 | Internet/ or social media/ |
| 43 | Online Social Networks/ |
| 44 | Mobile device*.ti,ab,id. |
| 45 | (online or internet or email* or e-mail* or electronic mail).ti,ab,id. |
| 46 | (world wide web or WWW or website).ti,ab,id. |
| 47 | (e-health or ehealth or electronic health).ti,ab,id. |
| 48 | (m-health or mhealth or mobile health).ti,ab,id. |
| 49 | Human Computer Interface/ or Human computer interaction.ti,ab,id. |
| 50 | (web adj based).ti,ab,id. |
| 51 | ((mobile or cellular or cell or smart) adj (phone* or telephone*)).ti,ab,id. |
| 52 | ((digital health or digital or mhealth) adj3 intervention*).ti,ab,id. |
| 53 | (interactive adj ((health adj communicat*) or video* or technolog* or multimedia)).ti,ab,id. |
| 54 | ((chat adj room*) or chatroom).ti,ab,id. |
| 55 | (phone adj app*).ti,ab,id. |
| 56 | 37 or 38 or 39 or 40 or 41 or 42 or 43 or 44 or 45 or 46 or 47 or 48 or 49 or 50 or 51 or 52 or 53 or 54 or 55 |
| 57 | Randomized controlled trial/ |
| 58 | Clinical trial/ |
| 59 | Randomized.ab. |
| 60 | Randomly.ab. |
| 61 | Trial.ab. |
| 62 | Groups.ab. |
| 63 | 57 or 58 or 59 or 60 or 61 or 62 |
| 64 | 28 and 36 and 56 and 63 |
| 65 | Animals/ not humans/ |
| 66 | 64 not 65 |
| 67 | Limit 66 to english language |

Scopus

TITLE ( exercis* OR "physical conditioning" OR running OR swimming OR walking OR "motor activity" OR "physical education and training" OR "physical fitness" OR "cardiorespiratory fitness" OR "life style" OR lifestyle OR "healthy ag?ing" OR "healthy diet" OR sport* OR "physical activit*" OR "physical inactivit*" OR sedentary OR "fitness class*" OR "fitness regime*" OR "fitness program*" OR diet OR nutrition* OR "child nutrition science*" OR fruit* OR vegetable* OR calorie* OR "energy intake" OR "energy density" OR "feeding behavio*" OR "dietary intake*" OR food OR "carbonated beverage*" OR "soft drink*" OR soda OR "sweetened drink" OR "dietary fat*" OR confectionar*) AND ABS ( postnatal OR post-natal OR perinatal OR peri-natal OR postpartum OR pregnancy OR parent* OR parent-targeted OR famil* OR infant* OR newborn* OR neonat* OR child OR baby or babies OR "early child*" OR maternal or mother*) AND ABS ( smartphone* OR "cell phone*" OR "smart phone*" OR "mobile device*" OR "mobile app*" OR "virtual reality" OR online OR internet OR "web based" OR "world wide web" OR www OR website* OR email* OR "e-mail*" OR "electronic mail*" OR "e-health" OR ehealth OR "electronic health" OR "m-health" OR mhealth OR "mobile health" OR ( digital AND intervention* ) OR ( ( interactive OR video* OR technolog* OR multimedia ) AND health ) OR "chat room*" OR chatroom* OR "phone app*" OR "user computer interface" OR "human computer interaction*" ) AND ( INDEXTERMS ( "clinical trials" OR "clinical trials as a topic" OR "randomized controlled trial" OR "Randomized Controlled Trials as Topic" OR "controlled clinical trial" OR "Controlled Clinical Trials" OR "random allocation" OR "clinical trial" OR "controlled study" OR "randomization" OR "placebo" ) ) AND ( LIMIT-TO ( EXACTKEYWORD,"Human" ) )
