# Supplementary material for: The Effectiveness of Parent-Targeted Digital Health Interventions on Breastfeeding Practices: Systematic Review and Meta-Analysis of Randomized Controlled Trials
Source: J Med Internet Res. 2026 Jul 2;28:e89214. doi: 10.2196/89214 (PMC13326728; doi:10.2196/89214)
Supplement: Multimedia Appendix 2 [file jmir-v28-e89214-s002.docx]

**Intervention details as per TIDier checklist and the socioecological model (SEM)**

| **Author, Year** | **Intervention Details as per TIDier Checklist** | **SEM components Targeted** |
| --- | --- | --- |
| Abbass-Dick et al (2020) [54] | *Name:* NA.  *Rational:* Providing mothers and their co-parents with education and support to assist them in meeting their BF goals has been found to increase BF outcomes. However, the most effective way to design interventions that include co-parents is unknown.  *Materials:* Co-parenting website (based on the BF Co-parenting Framework) covers: (1) Why BF; (2) How to BF; (3) The early days; (4) Common concerns; (5) Supporting mothers/fathers/partners; (6) Where to get help; (7) Everyday life; and (8) Helpful links. Included resources on how to work as a team to reach BF goals.  Who provided: Participants met with research staff to review how to use and access the website.  *Mode of delivery:* Website (Computer-Based eHealth).  *Where:* Online.  *When & how much:* Email reminders sent weekly for 6 weeks.  *Tailoring/Modification:* Not mentioned. | Individual (knowledge, behaviours and self-efficacy);  Interpersonal (coparent support); |
| Acar and Şahin (2024) [55] | *Name*: Mobile Application Based BF Program (MABP).  *Rational*: Mobile health-based BF support can be an effective method for offering BF support to mothers ‘anytime, anywhere’.  *Materials*: Mobile app-based BF education prepared in line the WHO and Turkey Ministry of Health recommendations. Includes 6 screens: (1) BF; (2) Expressing; (3) Contingencies; (4) FAQ; (5) BF log; (6) BF diary.  *Who provided*: Researchers.  *Mode of delivery*: Mobile app (Android and iOS) (mHealth + uHealth).  *Where*: Mobile app.  *When & how much*: Researchers sent weekly notifications via the app to increase motivation (approx. 8 weeks).  *Tailoring/Modification*: Problems with entering the program and BF entry were reported and resolved by the mobile application developer. | Individual (knowledge, behaviours) |
| Ahmed et al (2016) [56] | *Name*: NA.  *Rational*: Given the inconsistent evidence on the relationship between BF and post-partum depression (PPD), further research is warranted to explore whether web-based interventions can improve BF outcomes and reduce symptoms of PPD.  *Materials*: Web-based BF monitoring system developed with a self-regulation model. Mothers enter BF data and any problems for at least 30 days. Automatic notifications were sent back with tailored interventions to address the problem entered (e.g. newborn’s latch, sore nipples, engorgement). Professional educational resource also available in the system.  *Who provided*: Mother’s data was monitored by research assistant and lactation specialist. Mothers encouraged to contact their lactation specialist with any BF problems.  *Mode of delivery*: Computer application (Computer-based eHealth + uHealth).  *Where*: Online.  *When & how much*: Notifications were developed to target common BF problems in the 1^st^ few weeks following hospital discharge. System provided notifications if mother BF 8-10 times per day and read notifications.  *Tailoring/Modification*: Notifications tailored to the specific BF issues reported by Mothers. | Individual (knowledge, behaviours)  Community (lactation support) |
| Araban et al (2018) [57] | *Name:* NA.  *Rationale:* BF self-efficacy is an important variable because it is potentially modifiable and amenable to intervention. Low BF self-efficacy has been positively associated with perceptions of insufficient milk supply, a prevalent factor that leads to early discontinuation of BF and supplementation with formula worldwide.  *Materials:* The multifaceted BF self-efficacy intervention was developed from a Canadian BF self-efficacy intervention (McQueen et al., 2011) that was based on Dennis’s (1999) BF self-efficacy framework. It included two 1-hour group-based BF education sessions, an information booklet with BF images, and biweekly text messages. The text messages were sent to provide positive encouragement and reinforce EBF (verbal persuasion).  *Who provided:* A research nurse with extensive BF knowledge and experience in assisting BF women.  *Mode of delivery:* Text message (mHealth) and face to face.  *Where:* Mobile phone.  *When & how much:* The text messages were sent twice weekly after the first antenatal group session (35-37 weeks gestation) and continued until 8 weeks postpartum.  *Tailoring/Modification:* Not mentioned. | Individual (self-efficacy) |
| Baransel and Çalışkan (2024) [58] | *Name*: NA.  *Rational*: Education in the early postpartum period and the use of mobile messages after discharge, especially for first-time mothers on issues such as BF, self-care, and newborn care, where they have difficulties and need help, can be helpful in supporting the mother.  *Materials*: Mobile message content aligned with literature and WHO guidelines. Content included: 1) the importance of BF; 2) BF technique and thing to consider; 3) milking and storage conditions; 4) nipple problems; 5) postpartum mother-neonatal care; and 6) post-discharge emergencies. Intervention also included 2x BF education sessions (lasting 20-25 minutes each).  *Who provided*: Researchers.  *Mode of delivery*: Mobile messages (mHealth) and face to face.  *Where*: Mobile.  *When & how much*: Messages sent between 8am and 10am every day for 6 weeks following hospital discharge.  *Tailoring/Modification*: Not mentioned. | Individual (knowledge, self-efficacy) |
| Bender et al (2022) [59] | *Name*: NA.  *Rational*: There is a need to examine whether postpartum bidirectional text messaging can improve BF rates and exclusivity in diverse U.S. settings.  *Materials*: While pregnant, women received motivational BF text messages once weekly (as part of a run-in period- only women who responded to text messages during this time progressed to the next phase (i.e. randomisation)). After delivery mothers received informational and motivational text message-based content on BF in weekly intervals.  *Who provided*: Researcher (an obstetrician-gynaecologist).  *Mode of delivery*: Text-message (mHealth).  *Where*: Mobile phone.  *When & how much*: 6 messages over 6 weeks (one per week after birth).  *Tailoring/Modification*: Participants could ask questions that were responded to by the researcher (non-standardised responses). On occasion participants were referred for telehealth or in-person visits with healthcare professionals. | Individual (knowledge, motivation)  Community (healthcare professionals) |
| Bilgiç and Bozkurt (2024) [60] | *Name*: NA.  *Rational*: Evidence suggests that online postpartum support is an effective alternative to home visits, and Meleis’s Transition Theory could be a strong framework for guiding this support.  *Materials*: 2 online sessions. The first session covered: BF, structure of breast milk, advantages of BF, BF technique, expressing breast milk, storing expressed milk, infant health monitoring, newborn screening, vaccines, development of emotional bonding with baby. At the end of the session women were asked to write a letter to their babies and choose a lullaby. The second session covered: characteristics of the baby room, safety of the baby, sleep safety, prevention of falls and accidents and common problems. Two videos on baby bathing and massage were shown. Weekly phone calls up until baby was 3 months to explore BF problems. Mothers could contact the researchers at any time during the 3 months.  *Who provided*: Researcher.  *Mode of delivery*: Online sessions and phone calls (Computer-based eHealth + telehealth).  *Where*: Online and telephone.  *When & how much*: Online sessions delivered during prenatal period. The first online session lasted ~35 minutes. The second online session lasted ~20 minutes, and the two online videos were 18 minutes in total.  *Tailoring/Modification*: Some content was tailored to respond to specific concerns/questions. | Individual (knowledge), Community (healthcare professionals) |
| Bogaert et al (2024) [61] | *Name*: NA.  *Rational*: There is a need to examine whether an mHealth intervention using educational text messages and appointment reminders could support women in the vulnerable postpartum period by increasing adherence to care and attendance at scheduled clinic visits.  *Materials*: A pre-designed, online, text-based digital navigation program, where the patient’s phone number, postpartum appointment date, and delivery pathway were inputted. The system sent prewritten automated text messages to participants based on their postpartum appointment date. Included weekly text messages with educational information regarding postpartum care as well as specific text message appointment reminders for 6-week postpartum visits. Women who had conditions such as pregestational or intrapartum hypertension or delivery by caesarean section received additional educational messages around these conditions. Patients were able to access links to educational materials, as well as respond to the system to obtain phone numbers for social workers and nurses in the clinic.  *Who provided*: Rx.Health Digital Health Platform (Rx.Health Inc., New York).  *Mode of delivery*: Text-message (mHealth).  *Where*: Online.  *When & how much*: 3 text messages a week for 6 weeks.  *Tailoring/Modification*: Message content was tailored to patient inputted data. | Individual (knowledge), Community (healthcare professionals) |
| Bunik et al (2022) [62] | *Name*: Mother’s Milk Messaging.  *Rational*: New mothers experience BF challenges but have limited evidence-based technology enabled support.  *Materials*: Text message + app: daily text messages for 3-4 weeks before birth and up to 3 months after the birth. 20 messages received during pregnancy focused on benefits of BF and self-efficacy. 40 messages for post-partum centred on strategies for social support, enhanced behavioural skills and self-efficacy to overcome BF challenges. Messages were planned to be relevant to address specific issues for the BF journey. Messages based on social cognitive theory and the theory of planned behaviour. App included short videos, feed tracking features and content from BF Telephone Triage and Advice.  *Who provided*: Not mentioned.  *Mode of delivery*: Text message and app (mHealth + uHealth).  *Where*: Mobile phone.  *When & how much*: One message per day for 4 months.  *Tailoring/Modification*: Some participants had access to a doctor moderated private Facebook group.  Messages could be delivered in Spanish or English. | Individual (knowledge, self-efficacy, skills and behaviour)  Interpersonal (social support) |
| Can and Bulduk (2025) [63] | *Name*: WEB-BEP (Web-Based BF Education Program)  *Rational:* A method on the rise or increasingly popular for promoting BF is through web-based education interventions, but few have been developed and evaluated specifically for adolescent pregnant women.  *Materials:* Website compatible with any kind of device (computer, tablet or phone). Included professionally filmed videos covering expert approved BF content. Pregnant women could log in to the website to access and watch the videos at their convenience.  *Who provided:* Researcher delivered.  *Mode of delivery:* Online videos (Computer-based eHealth).  *Where:* Website was compatible with any kind of device.  *When & how much:* Website available for 8 weeks.  *Tailoring/Modification: Not mentioned.* | Individual (knowledge and skills) |
| Cavalcanti et al (2018) [64] | *Name*: Projeto Amamenta Mamãe (Mama Breastfeeding Project).  *Rational*: There is a need for more effective post-discharge support and technology-based interventions to help mothers sustain EBF.  *Materials*: A booklet with BF information (based on recommendations of the Brazil Ministry of Health and WHO) developed by the research team. The booklet included 24 topics and messages were direct, easy to read and understand. A closed group on the online social media network Facebook. Content on the network was based on the topics within the booklet, structured in a pedagogical manner. Messages were monitored by the project team.  *Who provided*: Researchers (academics, nutritionists and paediatricians).  *Mode of delivery*: Booklet and private Facebook group (Computer-based eHealth).  *Where*: Online.  *When & how much*: A new topic each week for 24 weeks.  *Tailoring/Modification*: Not mentioned. | Individual (knowledge) Interpersonal (social support) |
| Çelik and Toruner (2024) [65] | *Name*: NA.  *Rational*: Adolescent mothers often exhibit low BF rates, warranting evaluation of a technology support BF program designed for adolescent mothers.  *Materials*: 3 face-to-face interviews (week 1, week 4 and week 8) lasting up to 45 minutes. BF training and resources provided (written and PDF on android phones). Interviews included tailored BF problem solving. Mothers supported by telephone interviews (weeks 2, 3 and 6) to address any needs or problems related to infant care or BF (lasting 3-7 minutes on average). Access to 24/7 BF counselling via telephone as needed. Motivational text messages regarding BF were sent during weeks 2, 3, 4, 5, 6, 7 and 8.  *Who provided*: Researchers.  *Mode of delivery*: Face-to-face, telephone and text-message (telehealth + mHealth).  *Where*: Telephone/mobile.  *When & how much*: 3 x face-to-face interviews at week 1, 4 and 8. 3 x telephone interviews at weeks 2, 3 and 6, plus 24/7 telephone support. Text-messages at weeks 2, 3, 4, 5, 6, 7 and 8.  *Tailoring/Modification*: Tailored telephone counselling support offered, to address specific needs or problems. | Individual (knowledge, skills, self-efficacy and motivation), Interpersonal (social support). |
| Chegeni et al (2022) [66] | *Name*: NA.  *Rational*: Growing evidence suggests that e-learning and internet-based counselling can effectively support mothers, warranting evaluation of approaches such as telephone and social messaging app follow-up on BF self-efficacy and EBF continuation.  *Materials*: All participants received an educational package to promote BF, including 4 sections: 1) The importance and benefits of BF for mothers and babies; 2) Factors affecting the continuation of BF and EBF; 3) Correct BF technique; 4) Possible problems in the BF process.  Telephone group: The research called at 4 time-points to present a section of the educational material spending on average 20-30 minutes.  Social messenger group: A group was created in a social messaging app. Content was sent to their personal page at 4 time-points and shared in the created group. Mothers were asked to discuss their questions and experiences in the group and answer each other’s questions.  *Who provided*: Researchers.  *Mode of delivery*: Telephone or social messenger (telehealth + mHealth).  *Where*: Telephone or messaging.  *When & how much*: Contact days 1, 3, 5 and 7 after discharge.  *Tailoring/Modification*: Participants could ask questions. | Individual (knowledge, skills)  Interpersonal (social network- social messaging group only) |
| Davis et al 2023 [67] | *Name*: The Baby Bites Text Messaging Project.  *Rational*: Mobile health (mHealth), including text messaging, may be one cost-effective approach to influence early feeding decisions.  *Materials*: All participants received handouts on infant feeding and general infant safety advice. Intervention parents received gain framed text messages based on the Health Belief Model concept of perceived benefits, perceived barriers, self-efficacy and cues to action regarding healthy feeding practices. BF parents received messages promoting and supporting BF. Formula feeding parents received messages intended to provide cues to action regarding appropriate feeding practices. At 6 months, parents received a text to enrol in the introduction to solids group. Some messages contained links to a web page where parents could read content with more information on feeding advice.  *Who provided*: Not mentioned.  *Mode of delivery*: Text message (mHealth).  *Where*: A service called Healthy-Txt (Columbus, OH, USA) delivered the texts.  *When & how much*: 12-month intervention. Participants received a text four times/week for the first 4 weeks, two times/week by 5 weeks, and once a week from 8 weeks to 12 months of age.  *Tailoring/Modification*: Not mentioned. | Individual (knowledge, self-efficacy, motivation) |
| De Mello Sa et al (2025) [68] | *Name*: ‘Breastfeeding at AU’.  *Rational*: By exploring the potential of an mHealth intervention to support BF, this study has the potential to provide valuable insights into practical strategies for promoting maternal and child health.  *Materials*: A free Apple-Based smartphone application the participant can download. In the app, there is information and tips for BF success and continuation, as well as information on additional resources both online and in the community  *Who provided*: Not mentioned.  *Mode of delivery*: Mobile app (Apple based smartphone application) (mHealth).  *Where*: Mobile.  *When & how much*: Participants can access the app up to 12 months (follow-up).  *Tailoring/Modification*: Not mentioned. | Individual (knowledge, self-efficacy, motivation)  Community |
| Duong Doan et al (2022) [69] | *Name*: NA  *Rational*: Mobile phone-based interventions have the potential to improve BF outcomes, however, evidence is lacking on their effectiveness among women delivering by caesarean section, despite rising global caesarean rates.  *Materials*: The mobile application developed with a simple and user-friendly interface. Included auto-generated notifications that appear on the phone’s locked screen to encourage Mother’s use of the app. The app worked without internet access. Themes of the messages included: (1) believing breastmilk is the best option for newborns until 6 months; (2) believing mothers have enough milk and can BF early including those who have caesarean sections; (3) planning for EBF; (4) involving the husband and family members in preparing for EBF. Mothers were encouraged to involve health care providers and family members to support early and EBF.  *Who provided*: Not mentioned.  *Mode of delivery*: Mobile app (mHealth).  *Where*: Mobile phone.  *When & how much*: 3 messages were auto-generated weekly during pregnancy, and 12 key messages were repeated after 4 weeks. Another 48 messages were developed to send twice per week after delivery.  *Tailoring/Modification*: Not mentioned. | Individual (knowledge, self-efficacy)  Interpersonal (family support)  Community (health care providers) |
| Fan et al (2022) [70] | *Name*: NA.  *Rational*: To explore the feasibility and effectiveness of BF peer support delivered using an online text messaging group.  *Materials*: In addition to standard care, participants received peer support via a WhatsApp group. Peer counsellors encouraged women to discuss BF related issue, and provided emotional, informational and appraisal support. Participants were sent prompts asking for questions and providing BF related information. The platform offered a place to share advice, experiences and answer questions.  *Who provided*: 2 trained peer counsellors who had at least 2 months BF experience.  *Mode of delivery*: online instant messaging mobile app (WhatsApp) (mHealth).  *Where*: Mobile app.  *When & how much*: Weekly for 6 months.  *Tailoring/Modification*: Information shared was tailored to participant questions. | Individual (knowledge)  Interpersonal (social support) |
| Fiks et al (2017) [71] | *Name*: Grow2Gether.  *Rational*: Social media offers scalable, low-cost platform for low-income mothers to foster behaviours promoting healthy infant growth.  *Materials*: Participants joined a private Facebook peer group. Involved online group activities for 11 months (2 months prenatally, and until the infant was 9 months). Four separate peer groups including 9-13 women each, were formed based on due date. The curriculum included infant feeding practices, sleep, positive parenting, and maternal well-being based on Social Learning Theory. The group was structured around video-based content and encouraged participant interaction. Participants were required to post once in the group to receive a monthly stipend of $50/month for the first 8 months, and $10/month for the final 3 months.  *Who provided*: Each group was facilitated by a psychologist.  *Mode of delivery*: Facebook peer group.  *Where*: Online (plus 2 in-person meetings) (Computer-based eHealth).  *When & how much*: 1 short video/week for 6 months, then 1 short video/biweekly from 6-11 months.  *Tailoring/Modification*: Group members could post as much as desired. Participants provided feedback on one another’s post and the facilitator provided feedback. | Individual (knowledge)  Interpersonal (peer support)  Community (health professional) |
| Gilano et al (2025) [72] | *Name*: NA.  *Rational*: Evidence on the effectiveness of mHealth interventions for promoting EBF in Ethiopia is limited.  *Materials*: Tailored automated one-way messages and reminders sent to mobile phones.  *Who provided*: The FrontlineSMS application was customized and monitored by the trial manager (researcher) who tracked message delivery and reading status. Messages focus on antenatal care, postpartum family planning, vaccination, BF/nutrition, tips for maintaining EBF, danger signs, partner/community engagement, and reminders. Content was based on WHO guidelines, UNICEF BF guidelines, national Reproductive, Maternal, Newborn, Child Health and Family Planning strategies, and behaviour change communication frameworks (BCCI).  *Mode of delivery*: SMS (mHealth).  *Where*: Mobile.  *When & how much*: Pregnant women started receiving messages at 24-28 weeks gestation. Messages sent biweekly (every 2 weeks) for 9 months without interruption at 7am local time.  *Tailoring/Modification*: Information was ‘tailored’ – but unclear how. | Individual (knowledge), Interpersonal (partner engagement), Community (community engagement) |
| Gonzalez-Darias et al (2020) [73] | *Name*: Supporting a first-time mother.  *Rational*: Internet-based peer-support platforms offer a promising, accessible way to enhance BF outcomes in first-time mothers.  *Materials*: Website with updated information on BF and upbringing BF babies. Included a chat site, where supporters and mothers could interact and ask for advice regarding BF. Email notified mothers and supports on new messages posted on the website.  *Who provided*: Peer support volunteers (someone who has EBF to at least 6 months), who were provided 4-hour training from the research team (on common BF difficulties and how to advise women on those issues, and when to recommend women visit a health professional). The research team supervised all mothers-supporters’ interactions and the advice given to mothers.  *Mode of delivery*: Website (Computer-based eHealth).  *Where*: Online.  *When & how much*: UC.  *Tailoring/Modification*: Peer support volunteers answered questions and provided encouragement. | Individual (knowledge)  Interpersonal (peer support)  Community (Health professional) |
| Grijalva Eternod et al (2023) [74] | *Name*: The Cash for Improved Nutrition in Somalia study.  *Rational*: mHealth interventions show great promise in many areas of public health, but evidence for their impact on reducing the risk factors for malnutrition is uncertain.  *Materials*: A series of 30 pairs of health promoting audio messages. Each message included a 2-minute long drama and 1-minute-long reenforcing message sent on separate days to participant mobile phones. Message content related to: (1) Vaccination; (2) Water, sanitation and hygiene; (3) Infant and young child feeding practices; (4) Identifying signs of serious illness and seeking care; (5) Recognition, treatment and prevention of acute malnutrition; (6) Maximising health and nutrition for all household members. Messages recorded in Mahrati and Maay (2 most common languages in Somalia).  *Who provided*: Not mentioned.  *Mode of delivery*: Audio messages were prerecorded. Messages sent to recipient’s mobile phone (mHealth).  *Where*: Mobile phone.  *When & how much*: 5 seasons of the drama, each with 6 pairs of messages delivered over 8 months.  *Tailoring/Modification*: Participants received the messages in their preferred language. | Individual (knowledge)  Interpersonal (peer support) |
| Harari et al (2018) [75] | *Name*: LATCH pilot.  *Rational*: There are few trials of interventions to test the effectiveness of text messaging used to provide BF support.  *Materials*: Web-based 2-way texting intervention to improve BF rates. Participants provided evidenced-based BF education through automated texts, starting prenatally to 2 weeks postpartum. Messages were based on the theory of planned behaviour. Women could exchange texts with BF peer counsellor (PC). PC responded to texts between 8am-5pm, Monday-Friday.  *Who provided*: PC.  *Mode of delivery*: Web-based texting (mHealth + telehealth).  *Where*: Mobile phone.  *When & how much*: Participants sent 3 messages per week prenatally. 1 text 24-48 hours post-partum. 2-3 messages per day for 2 weeks.  *Tailoring/Modification*: Not mentioned. | Individual (knowledge);  Interpersonal (PC) |
| Hmone et al (2023) [76] | *Name*: M528.  *Rational*: No prior evaluations of public health interventions using mobile phones conducted in Myanmar have been reported.  *Materials*: Text messages informed by the Health Belief Model. Each text was 160 Myanmar characters. Texts were from a trusted source, short, locally acceptable, memorable and contained actionable content (cues to actions).  *Who provided*: Not mentioned.  *Mode of delivery*: Text-messages (mHealth).  *Where*: Mobile.  *When & how much*: 3 messages/week.  *Tailoring/Modification*: Messaging context was tailored to the mother’s stage of gestation and infants’ age in weeks. | Individual (knowledge) |
| Huang et al (2024) [77] | *Name*: NA.  *Rational*: There is a need to comprehensively explore the possible benefits and drawbacks of incorporating fathers into coparenting methods targeted at encouraging effective BF practices.  *Materials*: Parenting course, a father’s support group and individual counselling, covering 7 topics. Between 28-37 weeks of gestation, educational sessions focusing on BF significance and techniques were provided via WeChat video on the WeChat account. From 37 weeks gestation, information on BF in specific circumstances through text and video materials were delivered by the research team (supplemented by community nurses). Face to face researcher and community nurse meeting with couples at 14 days post-birth, plus handouts. 4 weeks postpartum, additional resources provided regarding postpartum depression, including textual, visual and video materials. Father support group via WeChat to share feelings and concerns. Couples could seek help through individual counselling as needed via WeChat or phone call.  *Who provided*: WeChat account developed by the research group. Research team consisted of international board-certified lactation consultant, a national second-level psychological counsellor, and postgraduate student major in maternal and child health.  *Mode of delivery*: WeChat public account named ‘Guardian of Maternal and Infant Health” (mHealth).  *Where*: Online, WeChat channel with resources, texts and calls.  *When & how much*: Commencing 28-37 weeks gestation until 4 weeks postpartum.  *Tailoring/Modification*: Beyond one-on-one counselling, not mentioned. | Individual (knowledge), Interpersonal (co-parent engagement and peer support)  Community (health professional) |
| Johnston et al (2025) [78] | *Name*: Mobile care companion programme.  *Rational*: More studies are needed to establish key strategies to improve the effectiveness of mobile messaging interventions, particularly in low- and middle-income countries.  *Materials*: Free mobile messaging service on WhatsApp. Delivers 25 messages, including seven videos, over 50 days. The messages are for entire families and align with the in-person programme, and promotes WHO-recommended postnatal practices, including early and EBF. Recipients can also ask questions on WhatsApp.  *Who provided*: Messages reviewed by a team of medical experts. Health educators encourage family members to engage with the service alongside new mothers.  *Mode of delivery*: WhatsApp messaging (mHealth).  *Where*: Mobile.  *When & how much*: 25 messages over 50 days.  *Tailoring/Modification*: Messages are available in 7 languages: English, Hindi, Kannada, Marathi, Punjabi, Tamil and Telugu. | Individual (knowledge), Interpersonal (targets households including fathers and grandmothers of newborns), Community (health professionals) |
| LeFevre et al (2022) [79] | *Name*: Kilkari.  *Rational*: Current evidence on large-scale mHealth messaging programmes is limited and inconsistent.  *Materials*: Pre-recorded calls about reproductive, maternal, neonatal and child health, starting from 2^nd^ trimester of pregnancy until the child is 1 yr old. Calls span on average of 77 seconds in duration and are framed as coming from ‘Dr Anita’.  *Who provided*: Not mentioned.  *Mode of delivery*: Pre-recorded calls.  *Where*: Mobile phone (telehealth).  *When & how much*: 72 once weekly voice calls: 24 during pregnancy; 24 within the first 6 months postpartum; 24 from 7-12 months postpartum.  *Tailoring/Modification*: Not mentioned. | Individual (knowledge) |
| Li et al (2024) [80] | *Name*: NA.  *Rational*: By integrating diverse forms of information, including text, images, videos and links to digital books and web pages, WeChat and WeChat Official Accounts, there is potential to enhance parental health literacy.  *Materials*: A specially designed WeChat Official Account was integrated into the WeChat App. Videos were developed in accordance with 12 sub dimensions of health literacy by the WHO. The videos included 4 video clips of experts’ talks between 13 and 33 minutes and 6 animated video clips of 1 to 5 minutes, totalling 2 hours. Additionally, participants were provided with reading materials from other trusted sources as supplementary materials to videos.  *Who provided*: WeChat.  *Mode of delivery*: Videos and related online content (mHealth).  *Where*: Online/Mobile.  *When & how much*: During the 9-month intervention, participants could self-navigate, select topics that they were interested in, and decided the order and pace of material they read or watched.  *Tailoring/Modification*: Beyond participants having the ability to self-navigate to content of interest, not mentioned. | Individual (knowledge) |
| Martinez-Brockman et al (2018) [81] | *Name*: LATCH trial.  *Rational*: Little evidence exists as to the effect of SMS interventions on BF behaviour.  *Materials*: 2-way text messaging platform that allowed prenatal and postpartum messages to be sent automatically according to a predetermined schedule. Content related to: (1) Benefits of BF; (2) examples of proper positioning; (3) if the baby is getting enough milk; (4) de-bunking BF myths. Messages based on the Health Actional Process Approach to behaviour change (to address self-efficacy and planning).  *Who provided*: Trained International Board-Certified Lactation Consultants trained PCs used the web-based platform to interact with participants prenatally and up to 3 months postpartum.  *Mode of delivery*: Mobile commons (web-based text messaging platform) (mHealth).  *Where*: Mobile.  *When & how much*: Text messages were sent with increasing frequency prenatally and decreasing frequency postpartum.  *Tailoring/Modification*: Participants received messages in language of preference (English or Spanish). | Individual (knowledge, self-efficacy);  Interpersonal (PC) |
| Maslowsky et al (2016) [82] | *Name*: NA.  *Rational*: Some mobile phone-based interventions in Latin America show promise, and in Ecuador mobile phone access is widespread.  *Materials*: Part 1: Educational session administered by a nurse via phone within 48 hours of hospital discharge Topics guided by a checklist/protocol. Part 2: Access to a nurse on-call during the first 30 days of a newborn’s life. Nurses provided medical advice, information and support and triaged patients to determine if a clinic visit was needed. Nurses were available via phone 8am-5pm Monday to Friday.  *Who provided*: A bachelor-degree-level, licensed Ecuadorian nurse with more than 15 years of clinical experience.  *Mode of delivery*: Phone call (telehealth).  *Where*: Mobile.  *When & how much*: 1 call within 48 hours of hospital discharge and access to on-call nurse for up to 30 days postpartum.  *Tailoring/Modification*: Nurse tailored content to participant questions. | Individual (knowledge);  Community (Health professional) |
| Miremberg et al (2022) [83] | *Name*: NA.  *Rational*: Studies that confirm the usefulness of mobile interventions and their impact on outcomes are still limited.  *Materials*: In addition to standard care, participants installed a web-based smartphone app. The app was in Hebrew and included information on the study and possible emotional challenges post-partum. Participants were encouraged to ask questions to the specialist team and received individualised responses via email.  *Who provided*: Specialist team included a maternal foetal medicine specialist, post-partum nurses, lactation consultants certified by the Israeli Ministry of Health, and a clinical psychologist.  *Mode of delivery*: Smartphone app (mHealth + telehealth).  *Where*: Mobile.  *When & how much*: The app was available for 6 months.  *Tailoring/Modification*: Tailored support to participant questions related to lactation tips/techniques, coping with challenges and positive feedback. | Individual (knowledge, skills, self-efficacy)  Community (Health professional) |
| Mukunya et al (2025) [84] | *Name*: NA.  *Rational*: The scale-up of timely initiation of BF is listed as a priority in the reduction of child mortality.  *Materials*:  3 x 20–60-minute face to face peer counselling (pre-delivery), plus 1 session within 3 days post-partum. Counselling took place at the mothers’ homes and involve mother, husband, mother-in-law, and any significant others. Peer counselling supplemented by mobile phone messaging. Mobile phone messages contained same topics discussed in counselling sessions: (1) encouraging health facility births; (2) birth preparedness; (3) early initiation of BF, and skin-to-skin care. Participants were also provided mama kits (i.e. clean delivery kits).  *Who provided*: Trained peer counsellors provided counselling.  *Mode of delivery*: Text-message system was automated with messages sent weekly until birth (mHealth).  *Where*: At home and mobile.  *When & how much*: Weekly messages during third trimester until birth. 4 face-to-face at home peer counselling sessions.  *Tailoring/Modification*: Unclear. Messages were validated and translated into Lango, the local language. | Individual (knowledge), Interpersonal (peer and household engagement) |
| Musiimenta et al (2022) [85] | *Name*: MatHealth App.  *Rational*: There is a lack of specific application of mHealth technologies targeting rural women with limited education.  *Materials*: Offline application including video/audio content, installed onto participant phones. The app displays notifications for new content tailored to each women’s stage of pregnancy which is pre-set in the app. The app was installed on low-cost smartphones and provided to participants at enrolment. Women were provided solar electricity charges. App included: (1) Video and audio files; (2) Appointment reminder; (3) Obstetrician connection.  *Who provided*: Not mentioned.  *Mode of delivery*: Mobile App (mHealth + uHealth).  *Where*: Mobile phone.  *When & how much*: On a monthly basis, the app displayed notifications of new offline videos tailored to each women’s stage of pregnancy which is pre-set within the app.  *Tailoring/Modification*: Content tailored to women’s stage of pregnancy. | Individual (knowledge)  Community (Health care; Health Professional) |
| Ogaji et al (2020) [86] | *Name*: NA.  *Rational*: Although beneficial effects of phone-based BF support have been demonstrated in low- and middle-income countries, there is still a paucity of data to demonstrate its effect on EBF practice and the nutritional indices of the babies whose mothers received this intervention in addition to the usual BF support provided in a Baby Friendly Hospitals.  *Materials*: Paediatrician mobile-phone advisory support service. Mothers received a structured instruction package (that guided telephone chats) covering topics including: (1) Benefits of EBF; (2) common BF questions; (3) Wellbeing of mother and baby.  *Who provided*: Paediatrician.  *Mode of delivery*: Phone calls (telehealth).  *Where*: Mobile phone.  *When & how much*: Participants contacted on the 7^th^ and 14^th^ day of each month post-birth, until the baby was 6 months old. An average of eight calls were made to each mother, and they could call back anytime they desire.  *Tailoring/Modification*: not mentioned. | Individual (knowledge)  Community (Health Professional) |
| Öksüz Sevda and Sevil (2023) [87] | *Name*: NA.  *Rational*: WhatsApp can be used to provide continuous lactation support, but there are few studies on this subject.  *Materials*: In additional to face-to-face group BF education. BF education content was delivered to participants via WhatsApp. Information included messages, audio recordings, images, videos encouraging BF knowledge and motivation. Participants could contact the researchers at any time, and problems were resolved using the plan-do-check-act cycle.  *Who provided*: Researcher.  *Mode of delivery*: WhatsApp (mHealth).  *Where*: Mobile phones/online.  *When & how much*: Content sent every day for first 10 days, and once per month until 6 months.  *Tailoring/Modification*: not mentioned. | Individual (knowledge and motivation) |
| Patel et al (2018) [88] | *Name*: NA.  *Rational*: BF practices can be enhanced through cell phone counselling. It can provide opportunity for early detection of BF problems, preventing of erroneous guidance by family members, friends, or health professionals, and reduce the need to visit a hospital.  *Materials*: Cell phone counselling provided from third trimester of pregnancy until a week after the infant was 6 months old. Counselling included: importance of antenatal care, iron-folic acid supplementation, maternal nutrition, appropriate feeding practices, avoiding pre-lacteal feeds, dealing with BF problems and infant immunisations. Women were provided cell phones, recharge vouchers and subsidised prepaid calling cards.  *Who provided*: Lactation counsellor: auxiliary nurse midwives with additional training for counselling over the phone.  *Mode of delivery*: Phone: Calls and text (telehealth + mHealth).  *Where*: Mobile phone.  *When & how much*: 1 counselling call per week. 1 text message per day, starting in third trimester until 6 months.  *Tailoring/Modification*: Women received text messages in regional language to augment appropriate feeding practices. | Individual (knowledge)  Community (lactation consultant and health professional) |
| Raj et al (2025) [89] | *Name*: NA.  *Rational*: As the penetration of mobile technology increases in India, video-based content for mobile devices can facilitate the dissemination of health education. WhatsApp: a mobile communication tools that supports video transfer, can be utilized for information and self-reinforcement.  *Materials*: Participants received an in person 30-minute one-on-one training session with the paediatric resident 24-48 hours before delivery. A video assisted tool about the method of BF (Free educational video by global health project media, duration 2 min and 25 seconds) which includes technique of BF translated and validated into Kannada language was shown during this session, then transferred via WhatsApp to participants smartphones.  *Who provided*: Paediatric resident provided first viewing of video.  *Mode of delivery*: The video of transferred via WhatsApp to participant smartphones (mHealth).  *Where*: Mobile/online.  *When & how much*: Sessions completed before birth.  *Tailoring/Modification*: Not mentioned. | Individual (knowledge), Community (health professional) |
| Sari and Altay (2020) [90] | *Name*: NA.  *Rational*: Limited studies have assessed the effect of web-based care programs beginning from the antenatal period to the postnatal period in primiparous women.  *Materials*: Web-based education program (informed by Pender’s Health Promotion Model) covering the benefits of BF, bathing and care, safe sleep and communication with baby in terms of maternal self-efficacy and infant care based on the concept of ‘perceived benefits’. The site included education subjects, videos, data collection instruments and ‘ask a question’.  *Who provided*: Not mentioned.  *Mode of delivery*: Website (Computer-based eHealth).  *Where*: Online.  *When & how much*: Unclear.  *Tailoring/Modification*: Not mentioned. | Individual (knowledge, self-efficacy) |
| Saucedo Baza et al (2023) [91] | *Name*: Breastfeeding at AU.  *Rational*: Data are limited on local BF rates, maternal BF self-efficacy, and the effectiveness of existing hospital-based educational programs.  *Materials*: App downloaded to participant smart phones immediately after enrolment (i.e. delivery). The app contained educational content related to: Benefits of BF; Infant BF needs; BF resources (e.g. videos and infographics from evidenced based sources). Content, educational videos and infographics obtained from evidenced based and government websites.  *Who provided*: Not mentioned.  *Mode of delivery*: Mobile app (apple devices for the pilot) (mHealth).  *Where*: Mobile.  *When & how much*: Access to the app for the duration of the study (6 weeks) and beyond.  *Tailoring/Modification*: Not mentioned. | Individual (knowledge, self-efficacy) |
| Schwarz et al (2024) [92] | *Name*: NA.  *Rational*: Mothers with greater awareness of the maternal benefits of lactation may have stronger and longer intensions to BF and may impact the duration of BF.  *Materials*:  Prenatal counselling intervention delivered using the Zoom videoconferencing platform. The virtual counselling session was scripted, lasting 10 minutes and addressed the maternal benefits of BF (written in Flesch-Kincaid Seventh Grade Level).  *Who provided*: Unclear.  *Mode of delivery*: Online (videoconferencing) (telehealth).  *Where*: Online.  *When & how much*: 1 x 10-minute session.  *Tailoring/Modification*: unclear. | Individual (knowledge) |
| Scott et al (2021) [93] | *Name*: Parent Infant Feeding Initiative: Milk Man.  *Rational*: mHealth interventions can provide the user with readily accessible information despite geographical distance or time constraints, and the immediacy offered by digital technologies provides users with information when it is most needed.  *Materials*: Milk Man app used gamification, social connectivity and push notifications to engage fathers with the app content and conversations. App included BF and parenting information with links to external websites.  *Who provided*: Not mentioned.  *Mode of delivery*: Smart phone app (Milk Man) (mHealth).  *Where*: Mobile.  *When & how much*: Twice weekly push notifications from 32 weeks gestation until 6 months post-partum.  *Tailoring/Modification*: Not mentioned. | Individual (knowledge, self-efficacy)  Interpersonal (engaged fathers) |
| Tizvir et al (2024) [94] | *Name*: NA.  *Rational*: The theory of planned behaviour (TPB) may positively influence BF rates. Considering the impact of continued BF on the health of infants, the decline of this index in the Iranian population, there is a need to investigate the impact of an education intervention based on the TPB on the continuity of BF among Iranian mothers.  *Materials*: Credible sources were used to inform the educational content, including from the WHO and CDC, resources developed between the Ministry of Health and Medical Education and UNICF Iran. Peer group education: group discussions with 10-12 mothers at health centres, included content related to 1) knowledge on continued BF; 2) Supplementary nutrition besides BF; 3)BF and body fitness; 4) BF and work. Pamphlet 1 and 2 was delivered to all, but 3 and 4 was optional based on mothers’ demographics. Peer groups included: group discussion, viewing of educational videos, dissemination of posters and printed materials, and all mothers received 1 educational SMS per week for 12 weeks.  Social network education: a group called “Mehr-e-Madar, Shir-e-Madar" (translates to “Mother’s Low, Mother’s Milk) was offered, and mothers interested were added to the group via Telegram. Participants could engage with media presentations and ask questions.  *Who provided*: A trained facilitator delivered group education. A physician with >20 years experience in child health education and BF oversaw the telegram group.  *Mode of delivery*: Videos and SMS. The social network education was launched on Telegram social network (mHealth + telehealth).  *Where*: Online and Mobile.  *When & how much*: Up to 4 peer education group sessions (minimum of 2). 1 SMS per week for 12 weeks. Access social networking group.  *Tailoring/Modification*: Not mentioned. | Individual (Knowledge, skills, motivation, attitude); Interpersonal (social network), Community (access to Health professionals) |
| Unger et al (2018) [95] | *Name*: Mobile WACh.  *Rational*: Maternal and child health SMS programmes are predominantly unidirectional (one-way). To compare the effect of one-way and two-way interactive SMS text messaging compared to control on EBF and maternal, neonatal and child health outcomes.  *Materials*: Both intervention groups accessed the Mobile WACh SMS platform. Women in the one-way messaging group received weekly push educational and motivational SMS. Women in the two-way messaging group received the same weekly SMS as the one-way, but each message contained questions, and women were encouraged to send an SMS with concerns or questions. SMS topics included antenatal care, pregnancy complications, family planning, infant health, EBF, infant immunisation and visit reminders.  *Who provided*: Nurses answered questions for the 2-way messaging group. One-way messages were automated.  *Mode of delivery*: SMS (mHealth).  *Where*: Mobile.  *When & how much*: Weekly ‘push’ SMS until 12 weeks post-partum.  *Tailoring/Modification*: Message delivery tailored to participant preference for message delivery language, day of the week and time of day for delivery. Participants received tailored content according to demographics (e.g. first-time mothers). Messages included participant name, clinic, nurse name and actionable advice targeting one of the main study outcomes. | Individual (knowledge and motivation)  Community (Health professional) |
| Vila-Candel et al (2024) [96] | *Name*: COMLACT study.  *Rational*: Various studies have examined the relationship between the use of applications and the maintenance of BF but found mixed results.  *Materials*: LactApp is an automated BF consultation system that includes a self-administered questionnaire that supports the tailoring of program content. The app enables BF tracking, BF related difficulties, mood and other aspects. Includes a live chat feature for users to discuss BF concerns. The app sends timely reminders (e.g. BF positions, feeding frequency, milk extraction techniques and significance of EBF) and covers various topics (e.g. baby care, weight gain, creating milk reserves and handling and storing milk).  *Who provided*: LactApp is an automated BF consultation system.  *Mode of delivery*: LactApp (mHealth + uHealth).  *Where*: Mobile.  *When & how much*: Unclear how many messages were sent during the 6 months study.  *Tailoring/Modification*: An initial questionnaire yields 2300 personalised answers, navigating through 76,100 potential paths, tailored to users’ profiles and selected options. Content has been developed in English and Spanish. | Individual (knowledge) |
| Wen et al (2020) [97] | *Name*: Healthy Beginnings.  *Rational*: Literature on telephone or SMS support for the appropriate timing of the introduction of solid foods and cup use is scarce.  *Materials*: Intervention content developed based on the Health Belief Model corresponding to key stages of child feeding and movement. Booklets were mailed to intervention groups matching the delivery timing of the telephone and SMS support. The telephone support group were called to discuss the information in the booklet. Support scripts were developed to assist the clinician with providing support. The SMS support group were sent SMS messages via a 2-way automated SMS system. Messages reinforced the information in the booklets.  *Who provided*: Telephone calls made by Child and Family Health Nurses. SMS messages were automated.  *Mode of delivery*: Telephone and SMS (mHealth + telehealth).  *Where*: Mobile phone.  *When & how much*: Telephone group were contacted 6 times during the intervention (1 in the third trimester and 5 postnatally up to 10 months). Calls lasted 30-60 minutes. SMS group were sent messages twice a week for 4 weeks (between 10am and 1pm) at each stage.  *Tailoring/Modification*: Not mentioned. | Individual (knowledge, motivation) |
| Wong and Chien (2023) [98] | *Name*: NA.  *Rational*: Early postpartum support including timely education, LC telephone support, and measures to strengthen BF self-efficacy is important to prevent early weaning and promoting EBF, particularly among first-time mothers.  *Materials*: Participant received an online BF talk covering common BF problems- content was designed according to Dennis’s BF self-efficacy framework. Five online sessions were held postnatally to increase BF self-efficacy, correct latch, address BF problems and provide encouragement. Women could share their BF experiences as part of a 10-minute online group meeting with other mothers.  *Who provided*: International Board-Certified Lactation Consultant.  *Mode of delivery*: Online (Zoom) and telephone (Computer-based eHealth + telehealth).  *Where*: Online.  *When & how much*: 1 x online BF talk (antenatal); 5 x daily online BF coaching (postnatal) and seven weekly telephone follow-ups.  *Tailoring/Modification*: Not mentioned. | Individual (knowledge, self-efficacy),  Interpersonal (peers),  Community (health professional) |
| Wu et al (2020) [99] | *Name*: ‘Ke Xue Wei Yang’ (Optimal Feeding).  *Rational*: No studies have focused on using WeChat to support caregivers with infant and young child feeding.  *Materials*: Participants were to subscribe and register to the WeChat module by entering their name, phone number, gestational age, expected date of delivery, and village and country of residence. 4 components to the Ke Xue Wei Yang module: 1) feeding messages; 2) feeding knowledge competition; 3) baby growth chart; and 4) online forum. BF messages were designed for BF promotion education (BF knowledge, advice, common problems and preparation for complementary feeding).  *Who provided*: Not mentioned.  *Mode of delivery*: WeChat (mHealth).  *Where*: Mobile.  *When & how much*: Messages could be read at any point after subscribing to the module. 3 additional sets of messages were sent during late pregnancy, 1 month and 4 months post-partum, on a Monday, Wednesday and Friday every week.  *Tailoring/Modification*: Messages were tailored to women’s stage of gestation or post-partum. | Individual (knowledge, skills) |

BF: Breastfeeding; mHealth: Mobile Health; EBF: Exclusive Breastfeeding
