# Supplementary material for: The Effectiveness of Parent-Targeted Digital Health Interventions on Breastfeeding Practices: Systematic Review and Meta-Analysis of Randomized Controlled Trials
Source: J Med Internet Res. 2026 Jul 2;28:e89214. doi: 10.2196/89214 (PMC13326728; doi:10.2196/89214)
Supplement: Multimedia Appendix 3 [file jmir-v28-e89214-s003.docx]

**Additional Exclusive Breastfeeding Meta-Analysis Figures (S1-S6).**


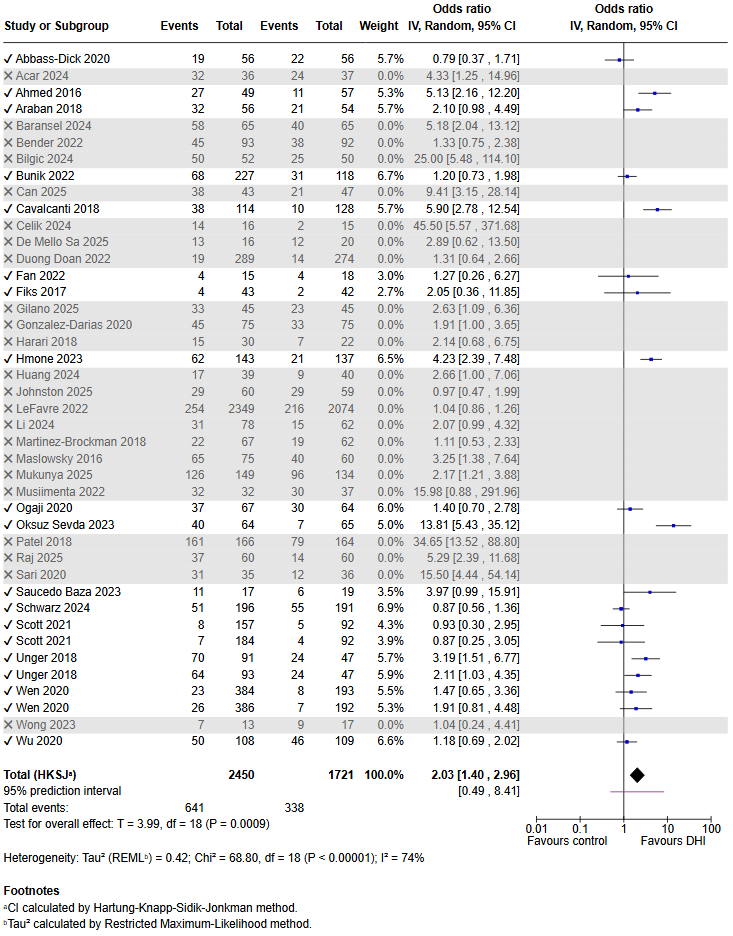


**Figure S1. Meta-analysis: Exclusive breastfeeding excluding high risk of bias trials.**

Sensitivity analysis excluding high risk of bias studies. Pooled effects from 16 randomized controlled trials conducted across diverse geographic settings evaluating digital health interventions (DHIs) targeting mothers, fathers, and other caregivers versus control conditions on exclusive breastfeeding (EBF) (N=4,171). EBF was assessed between 1 week and 6 months postpartum. Random-effects meta-analysis showed increased odds of EBF (odds ratio [OR] 2.03, 95% CI 1.40–2.96; I²=74%).

*Abbreviations: DHI: digital health intervention; EBF: exclusive breastfeeding; OR: odds ratio; I²: heterogeneity statistic.*


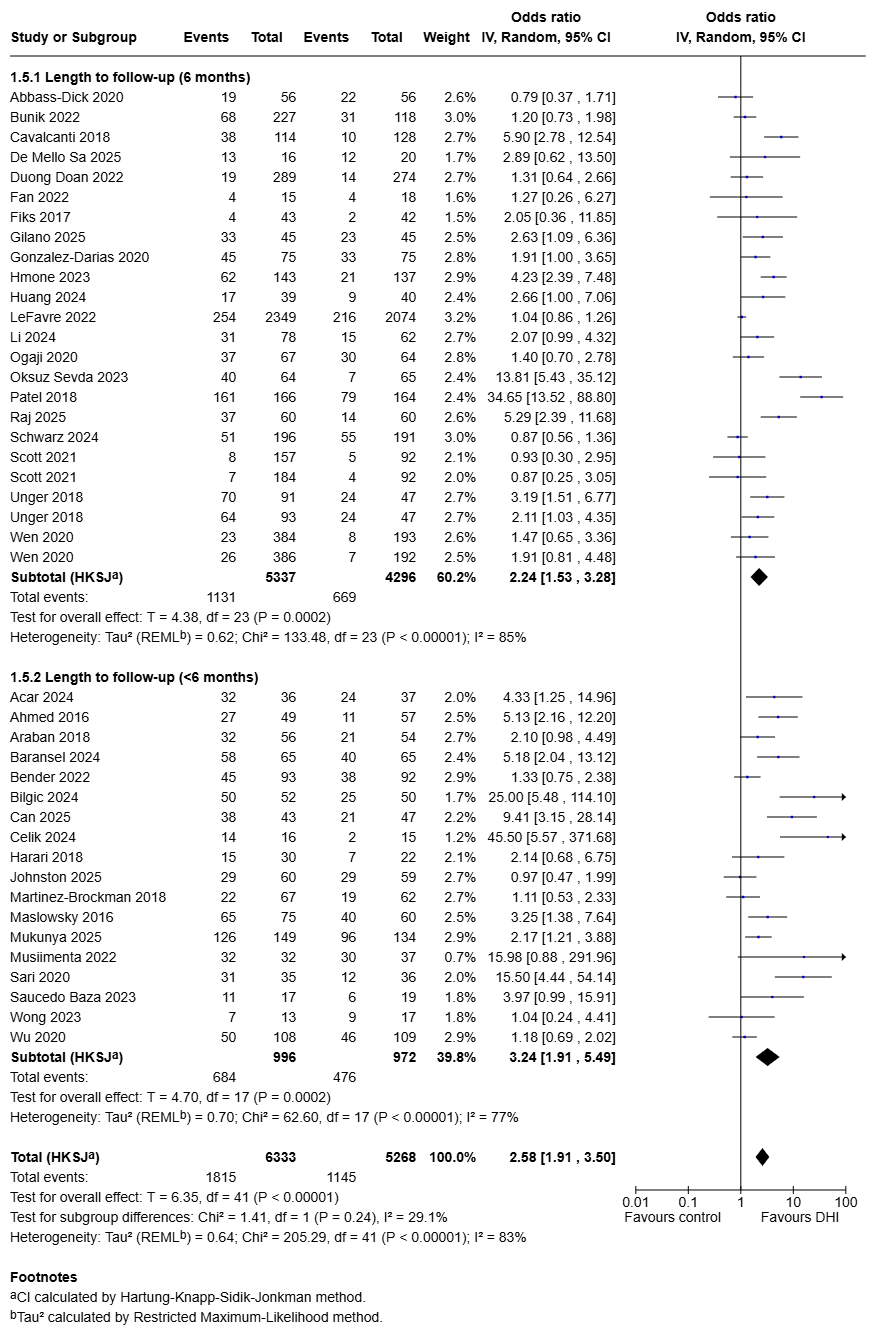


**Figure S2. Meta-analysis: Time to outcome measurement for exclusive breastfeeding.**

Trials stratified by timing of EBF assessment: <6 months versus 6 months postpartum. Subgroup analysis of 39 randomized controlled trials conducted across diverse geographic settings evaluating digital health interventions (DHIs) targeting mothers, fathers, and other caregivers compared with control conditions on exclusive breastfeeding (EBF). DHIs increased odds of EBF across both subgroups, and subgroup differences were not statistically significant (subgroup effect p=0.24). Substantial heterogeneity remained within subgroups (6 months follow-up: I²=85%; <6 months follow-up: I²=77%).

*Abbreviations: DHI: digital health intervention; EBF: exclusive breastfeeding; I²: heterogeneity statistic.*


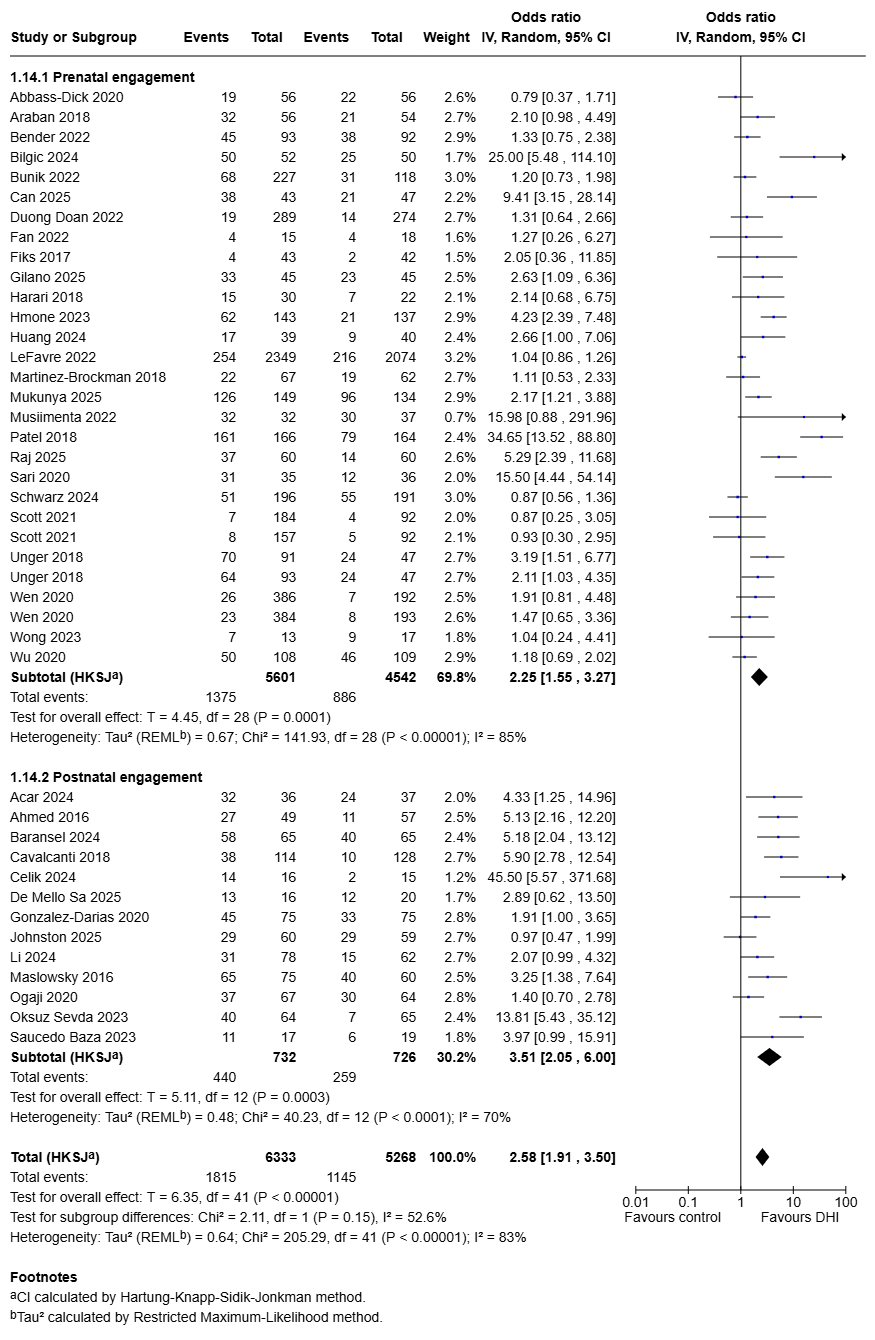


**Figure S3. Meta-analysis: Prenatal vs postnatal support for exclusive breastfeeding.**

Trials stratified by timing of intervention delivery: prenatal support versus postnatal-only support. Subgroup analysis of 39 randomized controlled trials conducted across diverse geographic settings evaluating digital health interventions (DHIs) targeting mothers, fathers, and other caregivers compared with control conditions on exclusive breastfeeding (EBF). DHIs increased odds of EBF in both subgroups, and subgroup differences were not statistically significant (subgroup effect p=0.15). Substantial heterogeneity remained within subgroups (prenatal support: I²=85%; postnatal support: I²=70%).

*Abbreviations: DHI: digital health intervention; EBF: exclusive breastfeeding; I²: heterogeneity statistic.*


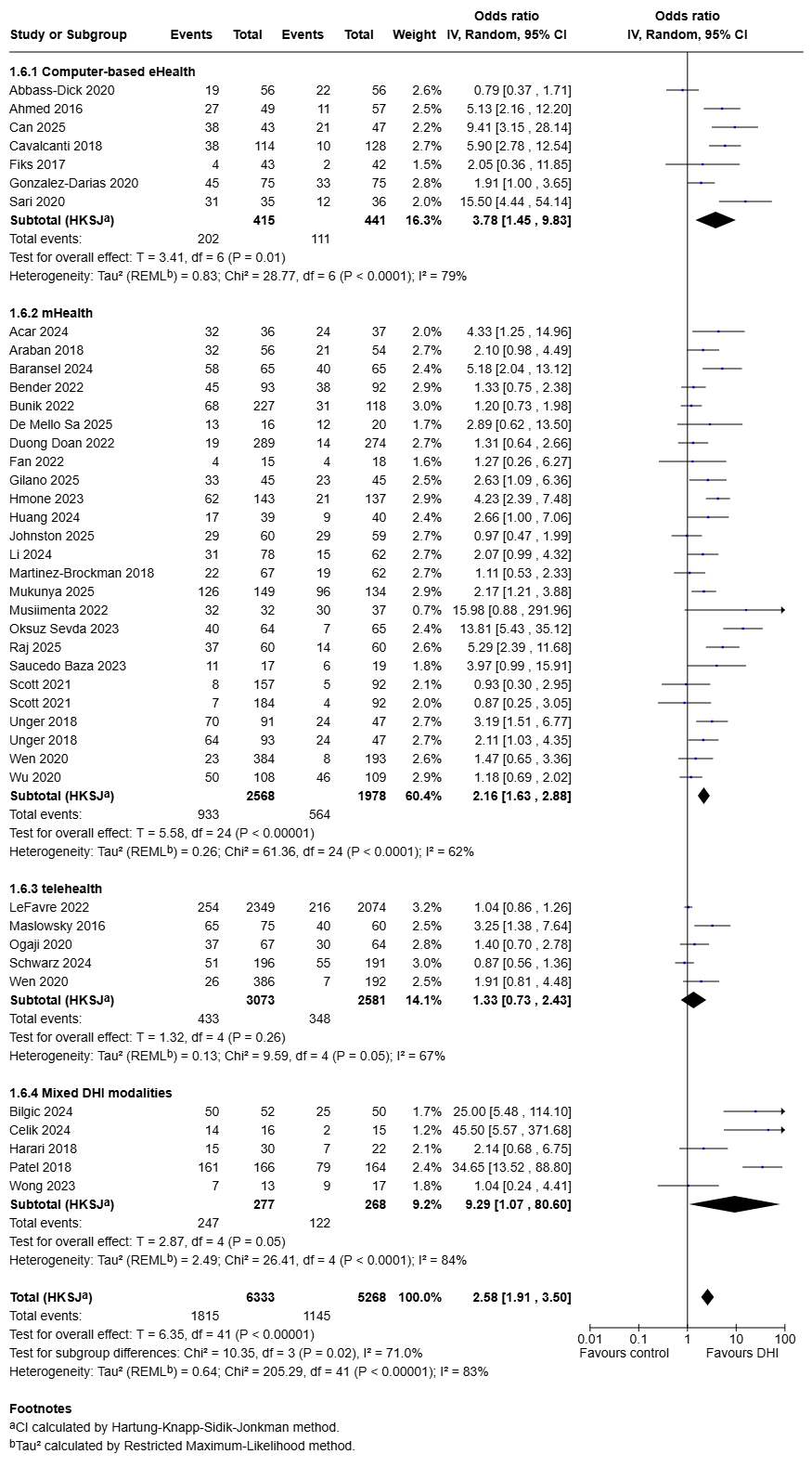


**Figure S4. Meta-analysis: Effect of digital health intervention modality on exclusive breastfeeding.**

Trials stratified by type of digital modality: computer-based eHealth, mHealth, telehealth and mixed. Subgroup analysis of 39 randomized controlled trials conducted across diverse geographic settings evaluating digital health interventions (DHIs) targeting mothers, fathers, and other caregivers compared with control conditions on exclusive breastfeeding (EBF). There was a significant subgroup effect (p=0.02), with the greatest effect observed for mixed modality interventions (OR 9.29, 95% CI 1.07–80.60), followed by computer-based eHealth (OR 3.78, 95% CI 1.45–9.83), mHealth (OR 2.16, 95% CI 1.63–2.88), and telehealth (OR 1.33, 95% CI 0.73–2.43).

*Abbreviations: DHI: digital health intervention; EBF: exclusive breastfeeding; OR: odds ratio; CI: confidence interval; I²: heterogeneity statistic.*


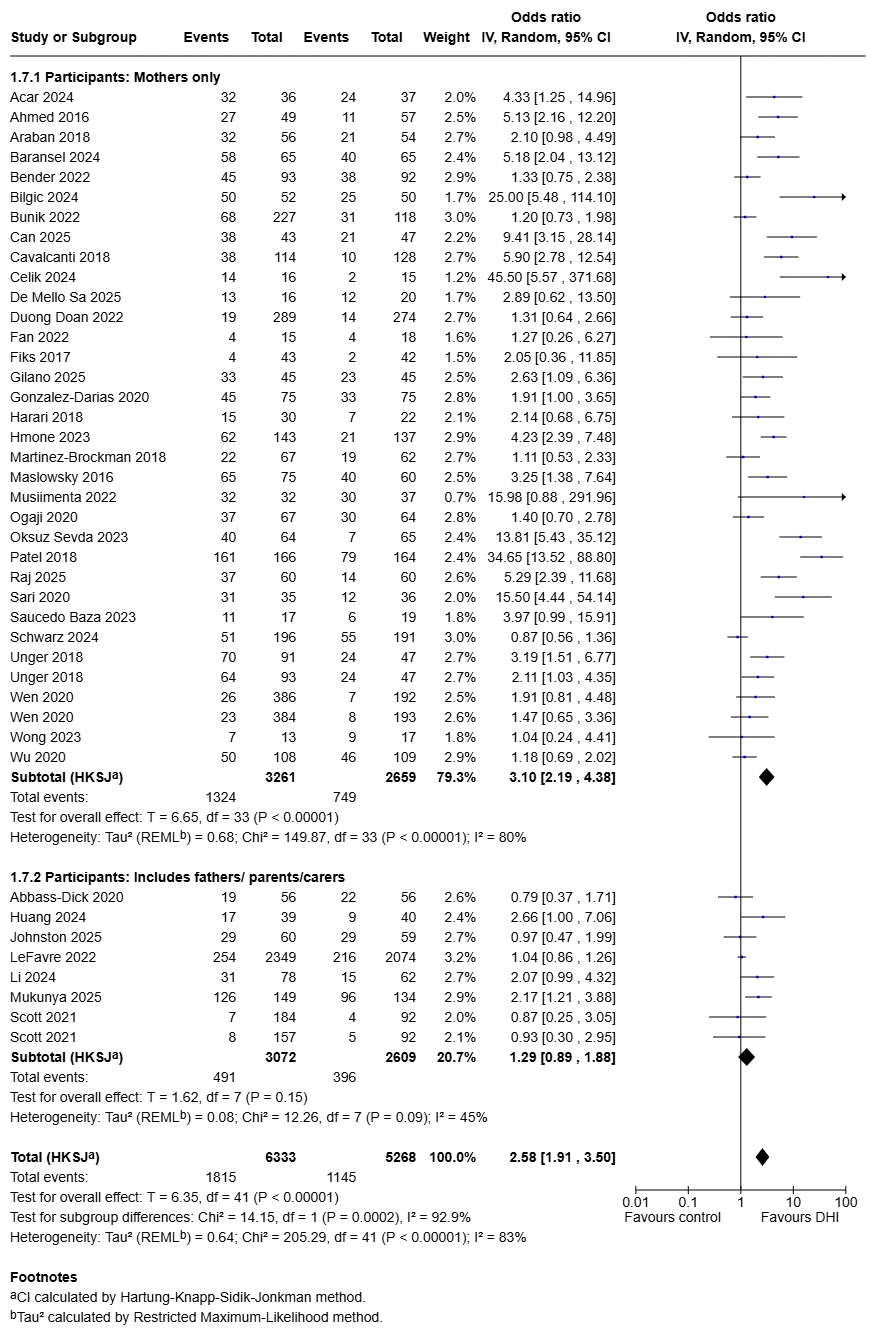


**Figure S5. Meta-analysis: Effect of digital health intervention target population on exclusive breastfeeding.**

Trials stratified by target population: mothers only versus fathers/parents/caregivers. Subgroup analysis of 39 randomized controlled trials conducted across diverse geographic settings evaluating digital health interventions (DHIs) compared with control conditions on exclusive breastfeeding (EBF). A significant subgroup effect was observed (p=0.0002), with greater impact for DHIs directed at mothers only (OR 3.10, 95% CI 2.19–4.38) than those directed at fathers/parents/caregivers more generally (OR 1.29, 95% CI 0.89–1.88; n=7 studies). High heterogeneity was present in the mothers-only subgroup (I²=80%).

*Abbreviations: DHI: digital health intervention; EBF: exclusive breastfeeding; OR: odds ratio; CI: confidence interval; I²: heterogeneity statistic.*


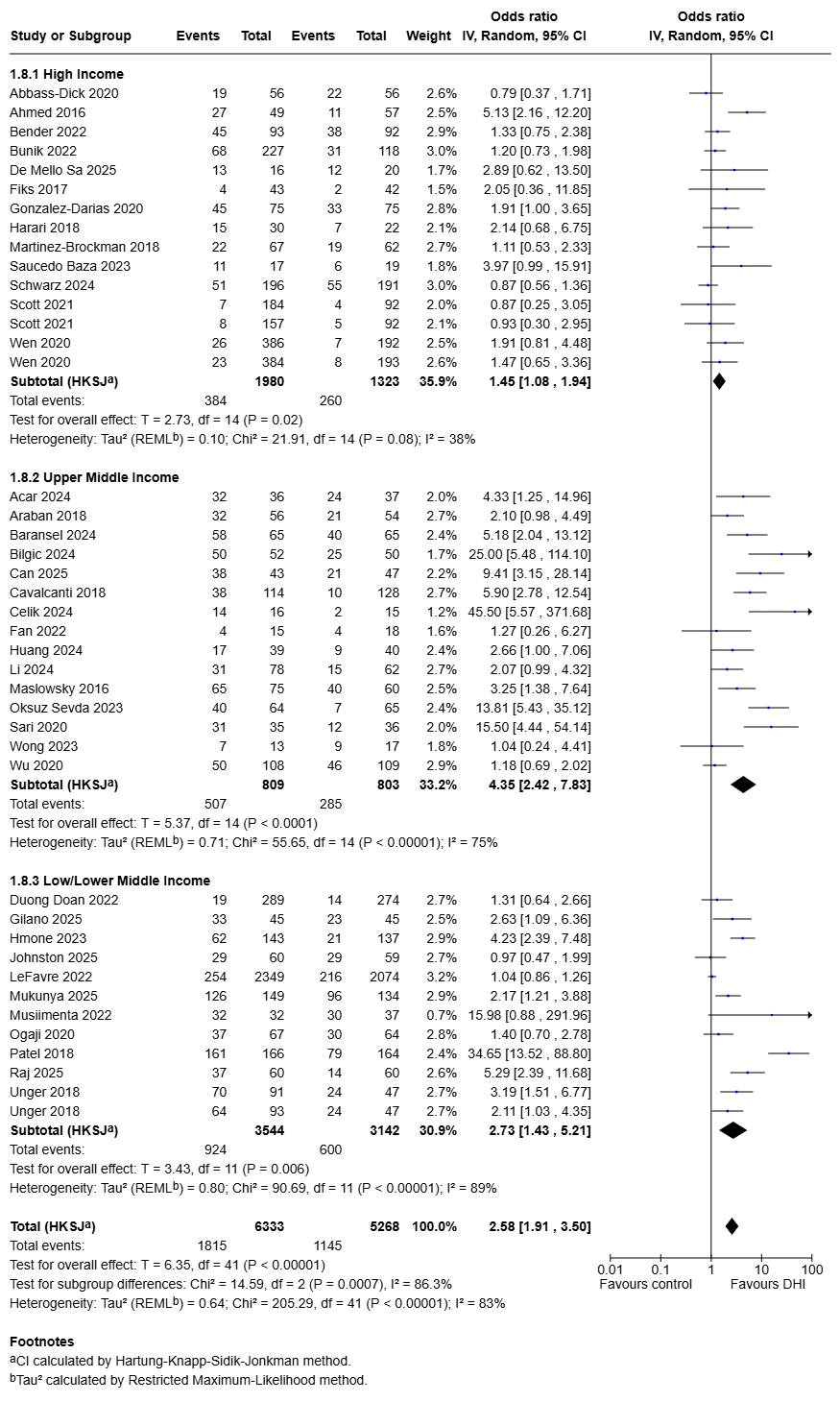


**Figure S6. Meta-analysis: Effect of country income on exclusive breastfeeding.**

Trials were grouped by country income: high, upper-middle, and low/lower-middle income. Subgroup analysis of 39 randomized controlled trials conducted across diverse geographic settings evaluating digital health interventions (DHIs) targeting mothers, fathers, and other caregivers compared with control conditions on exclusive breastfeeding (EBF). A significant subgroup effect was observed (p=0.0007), with greater impact for DHIs in upper-middle income countries (OR 4.35, 95% CI 2.42–7.83) and low/lower-middle income countries (OR 2.73, 95% CI 1.43–5.21) compared with high-income countries (OR 1.45, 95% CI 1.08–1.94). Heterogeneity was lowest in high-income countries (I²=38%) and higher in upper-middle (I²=75%) and low/lower-middle income countries (I²=89%).

*Abbreviations: DHI: digital health intervention; EBF: exclusive breastfeeding; OR: odds ratio; CI: confidence interval; I²: heterogeneity statistic.*
