# Supplementary material for: The Effectiveness of Parent-Targeted Digital Health Interventions on Breastfeeding Practices: Systematic Review and Meta-Analysis of Randomized Controlled Trials
Source: J Med Internet Res. 2026 Jul 2;28:e89214. doi: 10.2196/89214 (PMC13326728; doi:10.2196/89214)
Supplement: Multimedia Appendix 4 [file jmir-v28-e89214-s004.docx]

**Additional Any Breastfeeding Meta-Analysis Figures (S1-S6)**


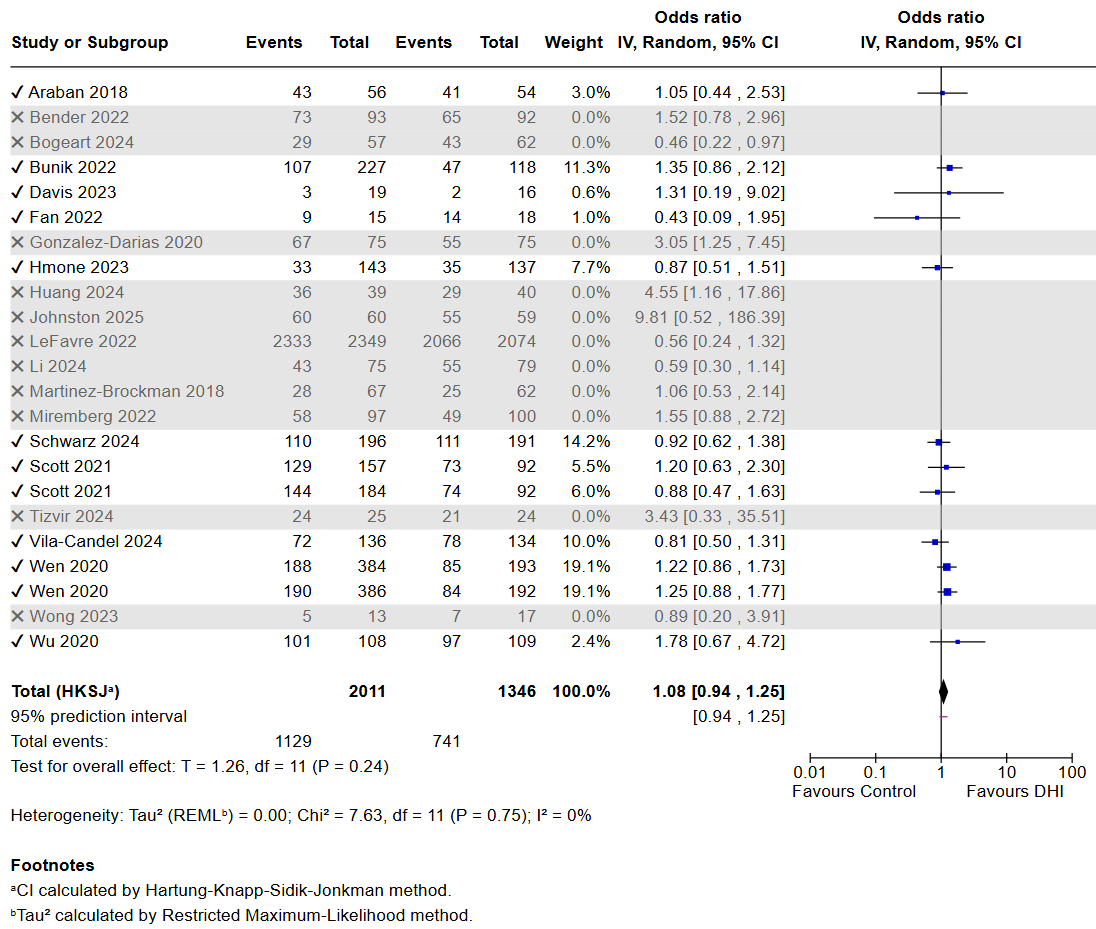
**Figure S1. Meta-analysis: Any breastfeeding excluding high risk of bias trials.**

Sensitivity analysis excluding high risk of bias studies. Pooled effects from 10 randomized controlled trials conducted across diverse geographic settings evaluating digital health interventions (DHIs) targeting mothers, fathers, and other caregivers compared with control conditions on any breastfeeding, excluding studies at high risk of bias (N=3,357 participants). Random-effects meta-analysis showed no significant effect of DHIs on any breastfeeding (odds ratio [OR] 1.08, 95% CI 0.94–1.25; I²=0%).

*Abbreviations: DHI: digital health intervention; OR: odds ratio; CI: confidence interval; I²: heterogeneity statistic.*


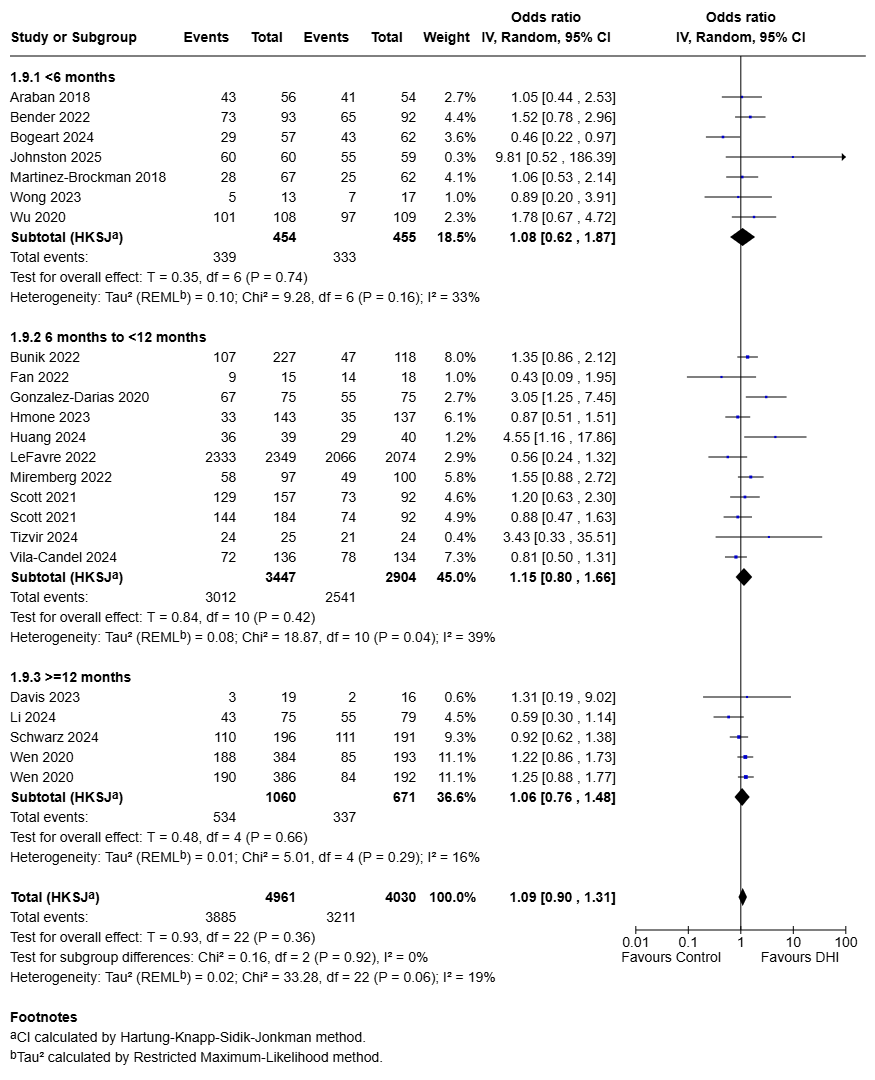


**Figure S2. Meta-analysis: Time to outcome measurement for any breastfeeding.**

Trials were stratified by timing of any breastfeeding assessment: <6 months, 6 months to <12 months and ≥12 months postpartum. Subgroup analysis of 21 randomized controlled trials conducted across diverse geographic settings evaluating digital health interventions (DHIs) targeting mothers, fathers, and other caregivers compared with control conditions on any breastfeeding. DHIs showed no significant effect on any breastfeeding across all subgroups, and subgroup differences were not statistically significant (subgroup effect p=0.92). Heterogeneity ranged from low to moderate across subgroups (<6 months follow-up: I²=33%; 6 months to <12 months follow-up: I²=39%; ≥12 months follow up: I²=16%). *Abbreviations: DHI: digital health intervention; I²: heterogeneity statistic.*


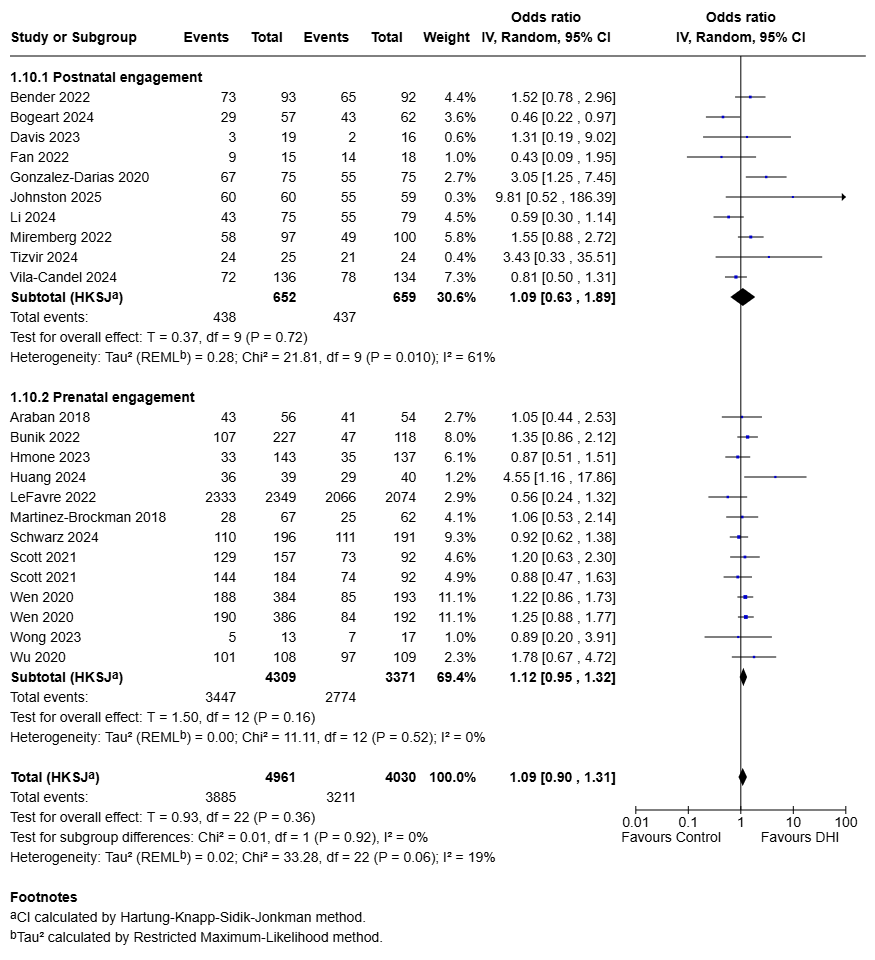


**Figure S3. Meta-analysis: Prenatal vs postnatal support for any breastfeeding.**

Trials were grouped by timing of intervention delivery: prenatal support versus postnatal-only support. Subgroup analysis of 21 randomized controlled trials conducted across diverse geographic settings evaluating digital health interventions (DHIs) targeting mothers, fathers, and other caregivers compared with control conditions on any breastfeeding. DHIs showed no significant effect on any breastfeeding across all subgroups, and subgroup differences were not statistically significant (subgroup effect p=0.92). Heterogeneity ranged from low to moderate across subgroups (prenatal support: I²=0%; postnatal support: I²=61%).

*Abbreviations: DHI: digital health intervention; I²: heterogeneity statistic.*


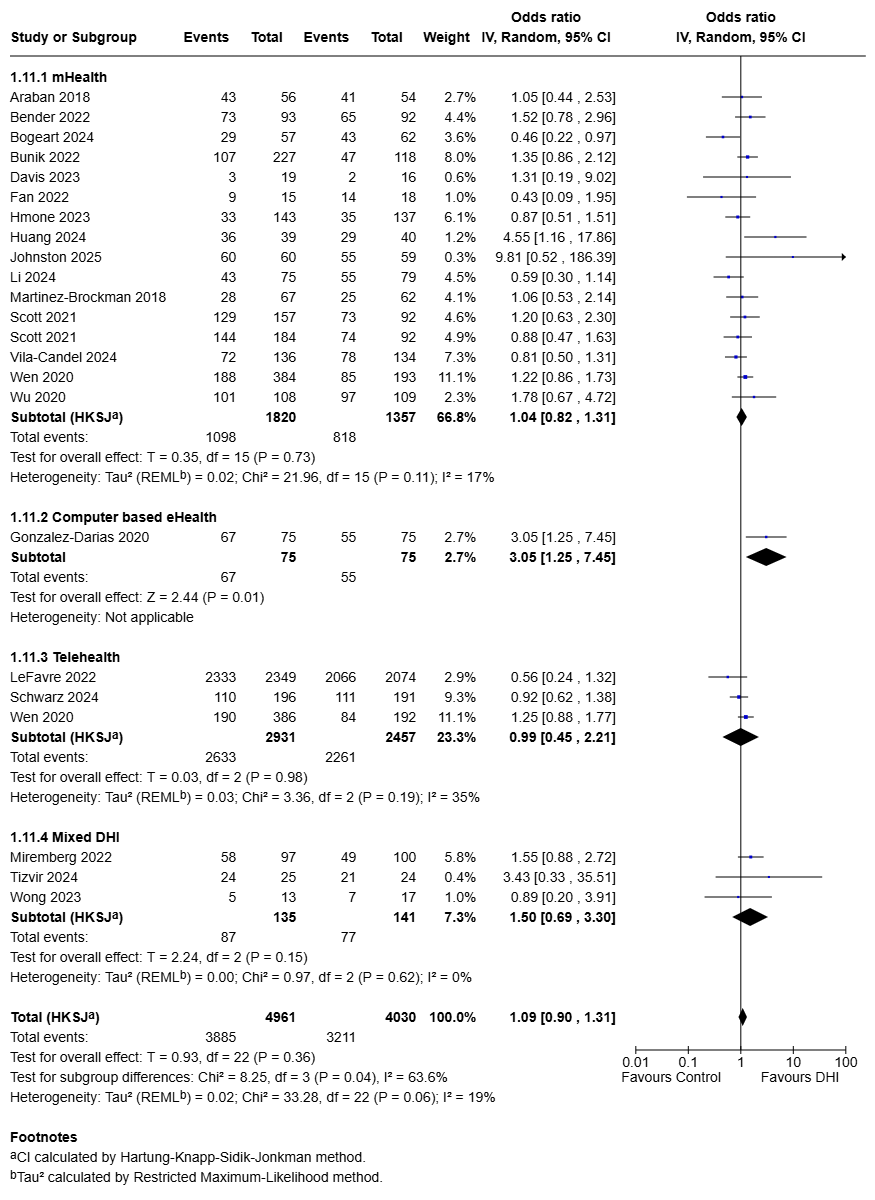


**Figure S4. Meta-analysis: Effect of digital health intervention modality on any breastfeeding.**

Trials were grouped by type of digital modality: computer-based eHealth, mHealth, telehealth and mixed. Subgroup analysis of 21 randomized controlled trials conducted across diverse geographic settings evaluating digital health interventions (DHIs) targeting mothers, fathers, and other caregivers compared with control conditions on any breastfeeding. Subgroup differences were statistically significant (p=0.04), however this is likely due to Gonzalez-Darias being grouped on its own (Computer based eHealth). Heterogeneity ranged from low to moderate across subgroups (computer-based eHealth: N/A; mHealth: I²=17%; telehealth: I²=35%; mixed: I²=0%). *Abbreviations: DHI: digital health intervention; I²: heterogeneity statistic.*


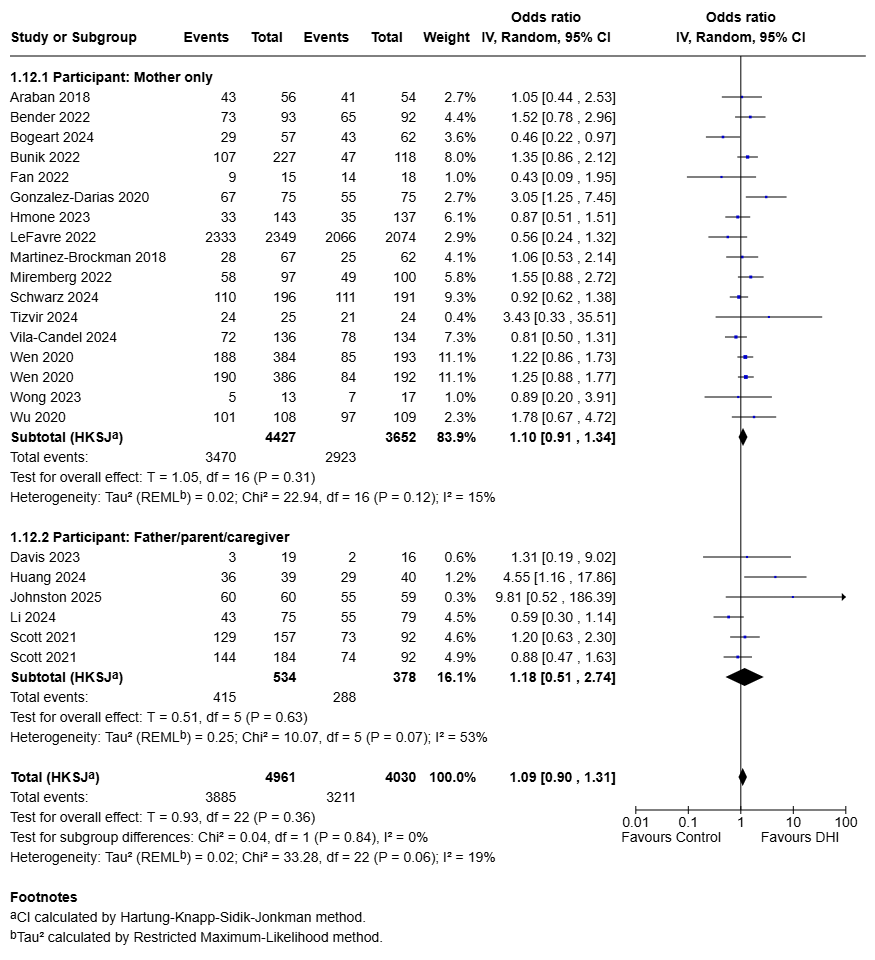


**Figure S5. Meta-analysis: Effect of digital health intervention target population on any breastfeeding.**

Trials stratified by target population: mothers only versus fathers/parents/caregivers. Subgroup analysis of 21 randomized controlled trials conducted across diverse geographic settings evaluating digital health interventions (DHIs) compared with control conditions on any breastfeeding. DHIs showed no significant effect on any breastfeeding across all subgroups, and subgroup differences were not statistically significant (p=0.84). Moderate heterogeneity was present both subgroups (Mothers only; I²=15%; Fathers/parents/caregivers; I²=53%).

*Abbreviations: DHI: digital health intervention; I²: heterogeneity statistic.*


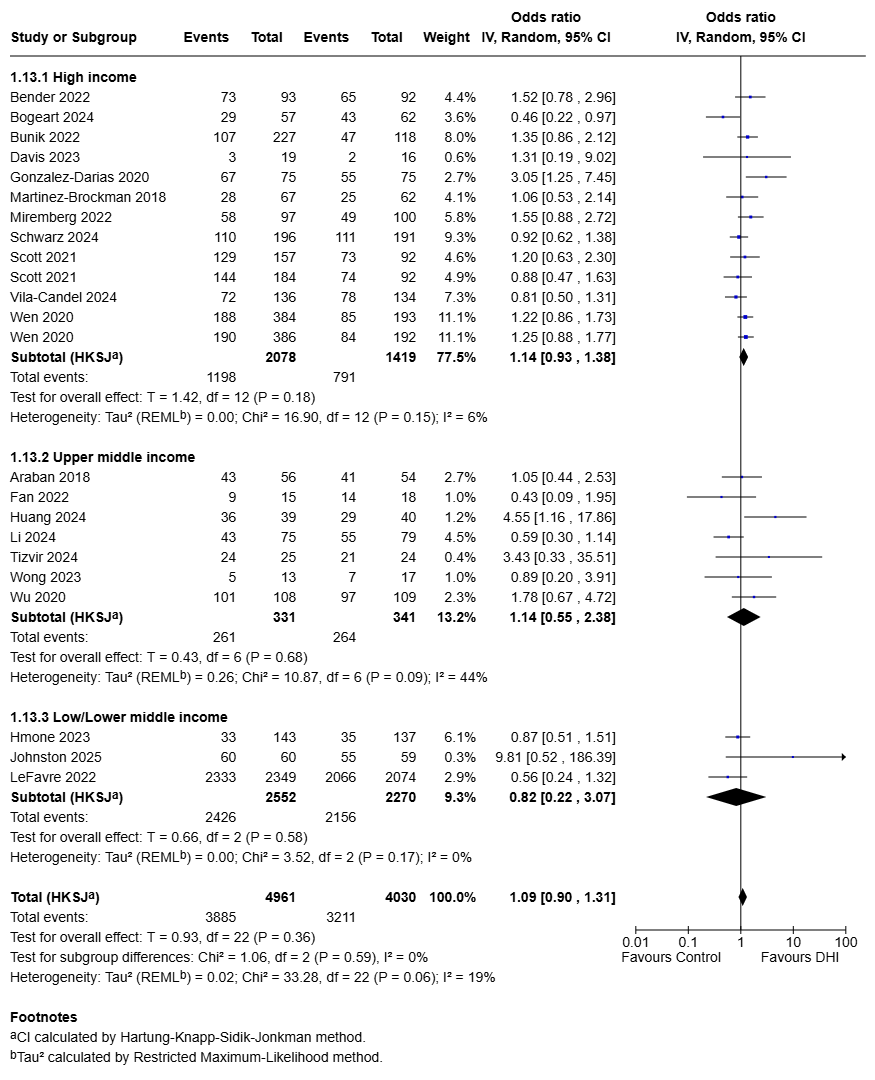


**Figure S6. Meta-analysis: Effect of country income on any breastfeeding.**

Trials were grouped by country income: high, upper-middle, and low/lower-middle income. Subgroup analysis of 21 randomized controlled trials conducted across diverse geographic settings evaluating digital health interventions (DHIs) targeting mothers, fathers, and other caregivers compared with control conditions on any breastfeeding. DHIs showed no significant effect on any breastfeeding across all subgroups, and subgroup differences were not statistically significant (p=0.59). Heterogeneity was lowest in low/lower middle income (I²=0%), followed by high-income countries (I²=6%) and moderate heterogeneity in upper-middle (I²=44%).

*Abbreviations: DHI: digital health intervention; I²: heterogeneity statistic.*
