# Supplementary material for: The Effectiveness of Parent-Targeted Digital Health Interventions on Breastfeeding Practices: Systematic Review and Meta-Analysis of Randomized Controlled Trials
Source: J Med Internet Res. 2026 Jul 2;28:e89214. doi: 10.2196/89214 (PMC13326728; doi:10.2196/89214)
Supplement: Multimedia Appendix 5 [file jmir-v28-e89214-s005.docx]

**Evidence t GRADE assessment: Funnel Plots (S1-S2)**


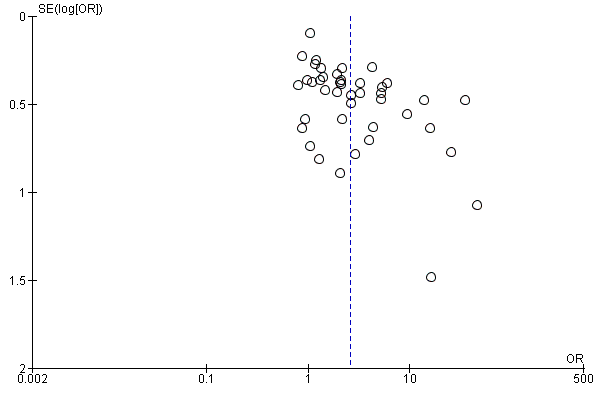


**Figure S1. Funnel Plot for EBF trials.** Given that most of the smaller trials sit on the right-hand side of the plot, this could indicate possible small study effects. Further data points in the lower left-hand quadrant are missing, suggesting possible publication bias.


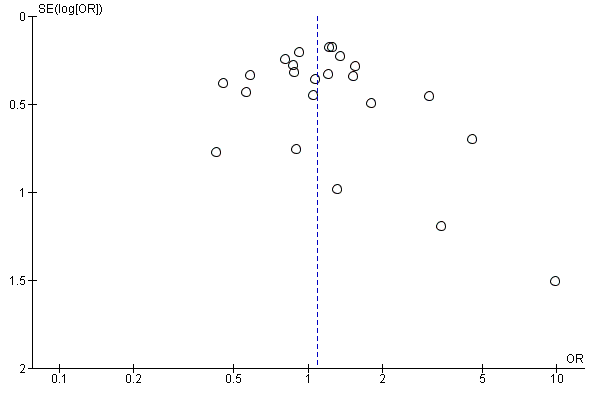


**Figure S2. Funnel plot for any breastfeeding trials.** Given that most of the smaller trials sit on the right-hand side of the plot, this could indicate possible small study effects. Further data points in the lower left-hand quadrant are missing, suggesting possible publication bias


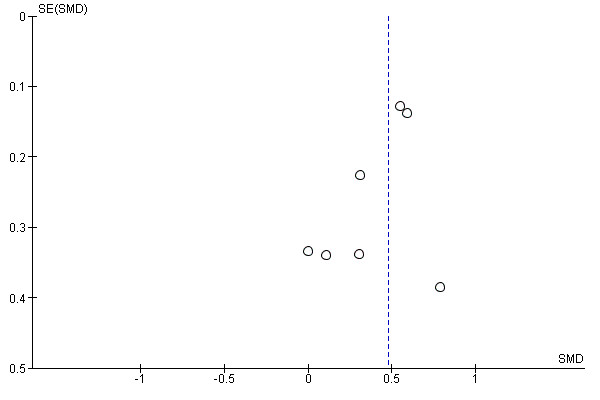


**Figure S3. Funnel plot for breastfeeding duration trials.** Given small trials sit on both sides of the plot, no clear signs of small study effects are indicated. Data points broadly resemble a pyramid, thus not indicating publication bias.
